# Supplementary material for: Temporal trends in relative survival following percutaneous coronary intervention
Source: BMJ Open. 2019 Feb 9;9(2):e024627. doi: 10.1136/bmjopen-2018-024627 (PMC6398900; doi:10.1136/bmjopen-2018-024627)

Supplementary Materials

Table A1 Procedure selection

	Procedures excluded		Procedures remaining	
	**N**	**Cumul. N**	**N**	**%**
Interventional procedures in England and Wales NHS, 2007 – 2014	-	-	575203	100.0
Exclude missing age and age<18 and age>100	439	439	574764	99.9
Exclude missing sex	984	1423	573780	99.8
Exclude missing indication and rescue/bail-out PCI	3887	5310	569893	99.1
Exclude missing mortality	20588	25898	549305	95.5

Table A2a Baseline patient characteristics for Elective procedures									
		**Female**				**Male**			
		**2007-2008**	**2009-2010**	**2011-2012**	**2013-2014**	**2007-2008**	**2009-2010**	**2011-2012**	**2013-2014**
**Number of procedures**		13847	13392	12603	12093	40246	40284	39715	39228
**Mean age, years (SD)**		67.1 (10.0)	67.8 (10.3)	68.3 (10.5)	68.5 (10.6)	63.7 (10.2)	64.2 (10.5)	64.6 (10.6)	64.9 (10.8)
**Variable, N (%)**									
Age	<50	796 (5.7)	738 (5.5)	636 (5.0)	624 (5.2)	3723 (9.3)	3702 (9.2)	3451 (8.7)	3325 (8.5)
	50s	2229 (16.1)	2034 (15.2)	1940 (15.4)	1854 (15.3)	9940 (24.7)	9289 (23.1)	9021 (22.7)	8961 (22.8)
	60s	4641 (33.5)	4193 (31.3)	3871 (30.7)	3563 (29.5)	14270 (35.5)	14097 (35.0)	13734 (34.6)	13249 (33.8)
	70s	4942 (35.7)	4820 (36.0)	4364 (34.6)	4235 (35.0)	10197 (25.3)	10486 (26.0)	10354 (26.1)	10191 (26.0)
	80+	1239 (8.9)	1607 (12.0)	1792 (14.2)	1817 (15.0)	2116 (5.3)	2710 (6.7)	3155 (7.9)	3502 (8.9)
Ethnicity	Asian	482 (5.1)	628 (6.0)	614 (6.2)	614 (6.4)	1777 (6.6)	2127 (6.8)	2329 (7.4)	2256 (7.3)
	Black	80 (0.9)	90 (0.9)	108 (1.1)	100 (1.0)	153 (0.6)	201 (0.6)	190 (0.6)	228 (0.7)
	White	7881 (84.2)	9216 (87.3)	8463 (85.2)	8045 (83.7)	22368 (82.5)	27231 (86.5)	26526 (84.3)	25584 (82.7)
	Other	918 (9.8)	619 (5.9)	748 (7.5)	851 (8.9)	2817 (10.4)	1908 (6.1)	2431 (7.7)	2863 (9.3)
Smoking	Never	5744 (49.5)	6037 (50.2)	6028 (53.1)	5722 (52.6)	11344 (33.5)	12089 (33.6)	12872 (36.2)	12941 (36.6)
	Ex-smoker	4368 (37.7)	4477 (37.2)	3944 (34.8)	3933 (36.1)	17927 (53.0)	19089 (53.0)	18131 (51.0)	18051 (51.1)
	Current	1487 (12.8)	1517 (12.6)	1376 (12.1)	1231 (11.3)	4547 (13.4)	4828 (13.4)	4538 (12.8)	4360 (12.3)
Dialysis		101 (0.7)	82 (0.6)	85 (0.7)	119 (1.0)	299 (0.7)	294 (0.7)	289 (0.7)	403 (1.0)
Diabetes		2736 (20.8)	2839 (21.7)	2809 (23.3)	2739 (23.5)	7466 (19.5)	8258 (21.1)	8361 (22.1)	8847 (23.5)
Previous PCI		2933 (22.7)	3600 (27.6)	3760 (30.6)	4043 (34.1)	10412 (27.8)	13404 (34.2)	14590 (37.7)	15981 (41.5)
Previous CABG		1336 (15.7)	850 (8.7)	810 (8.4)	766 (8.2)	5169 (20.5)	4116 (14.1)	4216 (13.6)	4161 (13.6)
Previous MI		3174 (27.1)	3640 (30.0)	3324 (28.4)	3267 (28.5)	12216 (35.9)	13370 (36.9)	13270 (36.2)	13861 (37.3)
Previous CVA		463 (3.5)	558 (4.3)	531 (4.4)	461 (4.0)	1149 (3.0)	1445 (3.7)	1403 (3.7)	1377 (3.7)
High cholesterol		8050 (61.6)	8780 (68.0)	7889 (65.4)	7464 (64.9)	22880 (60.3)	26255 (67.5)	24512 (64.8)	24058 (64.6)
Hypertension		7759 (59.4)	8633 (66.8)	8190 (67.9)	7803 (67.8)	19934 (52.6)	23528 (60.5)	23209 (61.4)	23498 (63.1)
Peripheral Vascular Disease		589 (4.5)	670 (5.2)	677 (5.6)	583 (5.1)	1739 (4.6)	2208 (5.7)	2103 (5.6)	1947 (5.2)
Valvular Heart Disease		163 (1.2)	236 (1.8)	324 (2.7)	330 (2.9)	318 (0.8)	534 (1.4)	660 (1.7)	776 (2.1)
LVEF	Good (>50%)	6106 (84.2)	6684 (83.3)	6158 (84.9)	6043 (84.3)	16388 (78.8)	18962 (78.2)	18424 (79.7)	18687 (79.4)
	Fair (30-50%)	871 (12.0)	1109 (13.8)	938 (12.9)	942 (13.1)	3338 (16.1)	4268 (17.6)	3851 (16.7)	3985 (16.9)
	Poor (<30%)	277 (3.8)	228 (2.8)	161 (2.2)	182 (2.5)	1069 (5.1)	1007 (4.2)	838 (3.6)	866 (3.7)
Chronic total occlusions		1396 (10.9)	1348 (11.2)	1203 (10.5)	1168 (10.5)	5159 (13.8)	5438 (14.8)	5239 (14.4)	5266 (14.4)
Access	Femoral only	9531 (74.2)	8080 (61.7)	6070 (48.9)	4348 (36.5)	26587 (71.0)	22678 (57.4)	16738 (42.8)	11676 (30.2)
	Radial only	3146 (24.5)	4724 (36.1)	5959 (48.0)	7073 (59.3)	10475 (28.0)	15995 (40.5)	21129 (54.0)	25454 (65.7)
	Multiple	118 (0.9)	252 (1.9)	321 (2.6)	456 (3.8)	298 (0.8)	650 (1.6)	1093 (2.8)	1456 (3.8)
	Other	48 (0.4)	48 (0.4)	55 (0.4)	46 (0.4)	80 (0.2)	154 (0.4)	142 (0.4)	137 (0.4)
LMS vessel PCI		345 (2.5)	439 (3.3)	462 (3.7)	597 (4.9)	1186 (2.9)	1371 (3.4)	1588 (4.0)	2164 (5.5)
Multivessel PCI		2080 (15.0)	2053 (15.3)	1832 (14.5)	1763 (14.6)	6966 (17.3)	6821 (16.9)	6751 (17.0)	6360 (16.2)

Table A2b Baseline patient characteristics for UA/NSTEMI procedures

		Female				Male			
		**2007-2008**	**2009-2010**	**2011-2012**	**2013-2014**	**2007-2008**	**2009-2010**	**2011-2012**	**2013-2014**
**Number of procedures**		12756	13858	15139	15594	33797	37049	39379	40749
**Mean age, years (SD)**		67.3 (11.5)	68.4 (11.9)	68.9 (12.2)	69.1 (12.2)	62.3 (11.8)	63.2 (11.9)	64.0 (12.2)	64.5 (12.3)
**Variable, N (%)**									
Age	<50	1072 (8.4)	1054 (7.6)	1193 (7.9)	1163 (7.5)	5161 (15.3)	5181 (14.0)	5069 (12.9)	4941 (12.1)
	50s	2009 (15.7)	2149 (15.5)	2199 (14.5)	2282 (14.6)	8787 (26.0)	9093 (24.5)	9418 (23.9)	9644 (23.7)
	60s	3665 (28.7)	3546 (25.6)	3767 (24.9)	3931 (25.2)	9925 (29.4)	10792 (29.1)	11207 (28.5)	11524 (28.3)
	70s	4228 (33.1)	4546 (32.8)	4764 (31.5)	4825 (30.9)	7452 (22.0)	8602 (23.2)	9195 (23.4)	9555 (23.4)
	80+	1782 (14.0)	2563 (18.5)	3216 (21.2)	3393 (21.8)	2472 (7.3)	3381 (9.1)	4490 (11.4)	5085 (12.5)
Ethnicity	Asian	510 (5.7)	565 (5.1)	617 (5.1)	792 (6.5)	1513 (6.4)	1848 (6.3)	2055 (6.6)	2317 (7.3)
	Black	65 (0.7)	102 (0.9)	136 (1.1)	139 (1.1)	163 (0.7)	240 (0.8)	242 (0.8)	280 (0.9)
	White	7764 (86.1)	9829 (88.2)	10607 (87.4)	10345 (84.9)	20211 (85.2)	25772 (87.3)	26894 (85.8)	26859 (84.3)
	Other	676 (7.5)	645 (5.8)	778 (6.4)	910 (7.5)	1847 (7.8)	1647 (5.6)	2158 (6.9)	2388 (7.5)
Smoking	Never	4403 (41.0)	5405 (43.9)	6332 (46.1)	6709 (47.0)	7547 (26.2)	9068 (27.2)	10987 (30.6)	11925 (31.7)
	Ex-smoker	3563 (33.2)	3855 (31.3)	4240 (30.9)	4459 (31.2)	12572 (43.7)	14408 (43.2)	15068 (41.9)	16149 (42.9)
	Current	2773 (25.8)	3058 (24.8)	3161 (23.0)	3116 (21.8)	8653 (30.1)	9860 (29.6)	9865 (27.5)	9603 (25.5)
Dialysis		140 (1.1)	139 (1.0)	165 (1.1)	215 (1.4)	365 (1.1)	364 (1.0)	452 (1.1)	523 (1.3)
Diabetes		2358 (19.5)	2858 (21.3)	3438 (23.6)	3777 (25.1)	5305 (16.6)	6561 (18.2)	7868 (20.8)	9027 (22.9)
Previous PCI		1741 (14.7)	2180 (16.2)	2555 (17.3)	3008 (19.7)	5000 (15.9)	6356 (17.7)	8101 (21.1)	8959 (22.4)
Previous CABG		891 (11.3)	748 (7.5)	778 (6.7)	816 (6.9)	3161 (14.8)	2781 (10.4)	3450 (11.3)	3461 (11.1)
Previous MI		3080 (28.9)	3298 (26.8)	3639 (25.8)	3833 (26.1)	8909 (31.6)	9730 (29.4)	11017 (30.1)	11336 (29.6)
Previous CVA		507 (4.2)	750 (5.6)	827 (5.7)	822 (5.5)	1074 (3.3)	1616 (4.5)	1777 (4.7)	1916 (4.9)
High cholesterol		7164 (58.9)	8344 (61.9)	8586 (58.9)	8454 (56.7)	18157 (56.5)	21576 (60.0)	21564 (56.9)	21501 (55.1)
Hypertension		6982 (57.4)	8447 (62.7)	9129 (62.7)	9540 (64.0)	15573 (48.5)	19115 (53.2)	20669 (54.5)	21892 (56.1)
Peripheral Vascular Disease		599 (4.9)	775 (5.8)	858 (5.9)	895 (6.0)	1486 (4.6)	2027 (5.6)	2232 (5.9)	2472 (6.3)
Valvular Heart Disease		118 (1.0)	207 (1.5)	301 (2.1)	335 (2.2)	223 (0.7)	328 (0.9)	542 (1.4)	684 (1.8)
LVEF	Good (>50%)	4523 (69.1)	5000 (67.4)	5340 (69.1)	5569 (68.0)	11530 (66.7)	12756 (64.8)	13090 (67.0)	13985 (65.9)
	Fair (30-50%)	1638 (25.0)	1982 (26.7)	1907 (24.7)	2102 (25.7)	4557 (26.4)	5540 (28.2)	5047 (25.8)	5570 (26.3)
	Poor (<30%)	382 (5.8)	440 (5.9)	478 (6.2)	520 (6.3)	1190 (6.9)	1378 (7.0)	1408 (7.2)	1652 (7.8)
Chronic total occlusions		647 (5.5)	578 (4.6)	623 (4.5)	572 (3.9)	1886 (6.1)	1836 (5.4)	1867 (5.2)	2007 (5.3)
Shock		135 (1.1)	175 (1.3)	205 (1.4)	217 (1.4)	319 (1.0)	417 (1.1)	475 (1.2)	605 (1.5)
Ventilated		95 (0.9)	104 (0.9)	168 (1.2)	163 (1.1)	218 (0.8)	327 (1.0)	449 (1.2)	530 (1.4)
IABP		177 (1.6)	192 (1.5)	211 (1.4)	160 (1.1)	398 (1.3)	443 (1.3)	471 (1.2)	395 (1.0)
Cardio-pulmonary support		4 (0.0)	9 (0.1)	10 (0.1)	7 (0.0)	11 (0.0)	21 (0.1)	17 (0.0)	19 (0.0)
Inotropes		47 (0.4)	71 (0.6)	77 (0.5)	77 (0.5)	113 (0.4)	149 (0.4)	163 (0.4)	231 (0.6)
Access	Femoral only	8724 (72.1)	7538 (55.4)	6202 (41.5)	4681 (30.4)	21963 (68.5)	18419 (50.6)	13905 (35.8)	10146 (25.2)
	Radial only	3209 (26.5)	5740 (42.2)	8236 (55.1)	10106 (65.7)	9759 (30.4)	17346 (47.7)	24030 (61.9)	28948 (72.0)
	Multiple	134 (1.1)	302 (2.2)	456 (3.1)	546 (3.6)	294 (0.9)	563 (1.5)	801 (2.1)	1019 (2.5)
	Other	39 (0.3)	34 (0.2)	48 (0.3)	46 (0.3)	57 (0.2)	74 (0.2)	90 (0.2)	86 (0.2)
LMS vessel PCI		434 (3.4)	594 (4.3)	749 (4.9)	807 (5.2)	971 (2.9)	1192 (3.2)	1607 (4.1)	2187 (5.4)
Multivessel PCI		2110 (16.5)	2159 (15.6)	2307 (15.2)	2394 (15.4)	5825 (17.2)	5951 (16.1)	6248 (15.9)	6772 (16.6)

Table A2c Baseline patient characteristics for STEMI procedures

		Female				Male			
		**2007-2008**	**2009-2010**	**2011-2012**	**2013-2014**	**2007-2008**	**2009-2010**	**2011-2012**	**2013-2014**
**Number of procedures**		4043	7608	10480	10908	12921	22306	30244	31066
**Mean age, years (SD)**		67.1 (12.7)	68.4 (12.9)	68.7 (13.2)	68.6 (13.3)	60.5 (12.3)	61.2 (12.4)	61.6 (12.6)	61.8 (12.4)
**Variable, N (%)**									
Age	<50	422 (10.4)	699 (9.2)	958 (9.1)	1035 (9.5)	2586 (20.0)	4178 (18.7)	5429 (18.0)	5350 (17.2)
	50s	678 (16.8)	1207 (15.9)	1653 (15.8)	1754 (16.1)	3545 (27.4)	6091 (27.3)	8176 (27.0)	8532 (27.5)
	60s	1042 (25.8)	1866 (24.5)	2539 (24.2)	2629 (24.1)	3564 (27.6)	6079 (27.3)	8360 (27.6)	8736 (28.1)
	70s	1202 (29.7)	2179 (28.6)	2898 (27.7)	2879 (26.4)	2364 (18.3)	4157 (18.6)	5509 (18.2)	5610 (18.1)
	80+	699 (17.3)	1657 (21.8)	2432 (23.2)	2611 (23.9)	862 (6.7)	1801 (8.1)	2770 (9.2)	2838 (9.1)
Ethnicity	Asian	164 (5.4)	269 (4.3)	397 (4.4)	389 (4.3)	802 (8.3)	1296 (7.2)	1845 (7.1)	1801 (7.0)
	Black	27 (0.9)	41 (0.7)	67 (0.7)	74 (0.8)	102 (1.1)	144 (0.8)	193 (0.7)	226 (0.9)
	White	2674 (88.0)	5705 (91.6)	8150 (90.8)	8221 (90.8)	8110 (84.4)	15982 (88.2)	22654 (87.5)	22384 (87.1)
	Other	173 (5.7)	212 (3.4)	363 (4.0)	368 (4.1)	591 (6.2)	696 (3.8)	1185 (4.6)	1279 (5.0)
Smoking	Never	1359 (39.7)	2603 (38.6)	3991 (43.1)	4167 (43.2)	2910 (26.1)	5470 (27.4)	8538 (31.4)	8956 (32.0)
	Ex-smoker	790 (23.1)	1542 (22.9)	1904 (20.6)	2155 (22.3)	3530 (31.7)	5900 (29.6)	7607 (28.0)	8085 (28.8)
	Current	1278 (37.3)	2593 (38.5)	3357 (36.3)	3325 (34.5)	4690 (42.1)	8575 (43.0)	11042 (40.6)	10989 (39.2)
Dialysis		32 (0.8)	40 (0.5)	38 (0.4)	46 (0.4)	79 (0.6)	85 (0.4)	125 (0.4)	127 (0.4)
Diabetes		639 (16.7)	1091 (14.8)	1557 (15.6)	1744 (16.9)	1609 (13.1)	2681 (12.4)	3877 (13.5)	4320 (14.6)
Previous PCI		314 (8.3)	551 (7.4)	751 (7.4)	798 (7.5)	1287 (10.7)	2233 (10.3)	2938 (10.0)	3401 (11.2)
Previous CABG		195 (8.7)	154 (2.9)	256 (3.3)	269 (3.4)	707 (9.8)	727 (4.7)	959 (4.2)	1033 (4.6)
Previous MI		584 (16.3)	840 (12.1)	1037 (10.7)	1074 (10.6)	2150 (18.8)	2976 (14.7)	3761 (13.4)	4021 (13.9)
Previous CVA		157 (4.1)	362 (5.0)	461 (4.6)	487 (4.7)	375 (3.1)	699 (3.3)	984 (3.4)	975 (3.3)
High cholesterol		1695 (44.5)	3142 (43.0)	3997 (40.0)	3987 (38.9)	5091 (41.8)	9063 (42.3)	11286 (39.2)	11200 (38.3)
Hypertension		1838 (48.3)	3503 (47.9)	4776 (47.8)	5013 (48.9)	4649 (38.2)	8427 (39.4)	11215 (39.0)	11724 (40.1)
Peripheral Vascular Disease		140 (3.7)	253 (3.5)	374 (3.7)	395 (3.9)	420 (3.4)	657 (3.1)	953 (3.3)	952 (3.3)
Valvular Heart Disease		27 (0.7)	47 (0.6)	131 (1.3)	107 (1.0)	44 (0.4)	80 (0.4)	198 (0.7)	172 (0.6)
LVEF	Good (>50%)	648 (47.3)	1136 (47.9)	1669 (53.1)	1572 (47.9)	2183 (49.7)	3452 (48.0)	4926 (53.9)	4749 (49.1)
	Fair (30-50%)	556 (40.6)	957 (40.4)	1183 (37.7)	1338 (40.7)	1560 (35.5)	2883 (40.0)	3380 (37.0)	3842 (39.8)
	Poor (<30%)	166 (12.1)	277 (11.7)	290 (9.2)	375 (11.4)	646 (14.7)	864 (12.0)	830 (9.1)	1073 (11.1)
Chronic total occlusions		172 (4.6)	269 (3.9)	218 (2.3)	239 (2.4)	662 (5.5)	855 (4.2)	705 (2.6)	769 (2.7)
Shock		325 (8.2)	526 (7.0)	851 (8.2)	1055 (9.8)	811 (6.4)	1364 (6.2)	1973 (6.6)	2482 (8.1)
Ventilated		113 (3.3)	198 (3.0)	371 (4.0)	458 (4.7)	318 (2.9)	622 (3.2)	1201 (4.5)	1477 (5.3)
IABP		272 (7.3)	391 (5.7)	478 (4.8)	381 (3.6)	718 (6.0)	1117 (5.5)	1334 (4.7)	1090 (3.6)
Cardio-pulmonary support		10 (0.3)	23 (0.3)	44 (0.4)	28 (0.3)	30 (0.3)	57 (0.3)	99 (0.3)	82 (0.3)
Inotropes		123 (3.3)	178 (2.6)	268 (2.7)	323 (3.1)	272 (2.3)	428 (2.1)	579 (2.0)	798 (2.7)
Access	Femoral only	2942 (75.9)	4441 (59.5)	4573 (44.3)	3406 (31.7)	8928 (71.9)	11990 (54.6)	11216 (37.7)	7580 (24.8)
	Radial only	868 (22.4)	2776 (37.2)	5385 (52.1)	6881 (63.9)	3314 (26.7)	9483 (43.2)	17852 (60.0)	22168 (72.4)
	Multiple	60 (1.5)	237 (3.2)	367 (3.6)	449 (4.2)	147 (1.2)	439 (2.0)	658 (2.2)	806 (2.6)
	Other	6 (0.2)	11 (0.1)	9 (0.1)	24 (0.2)	22 (0.2)	32 (0.1)	45 (0.2)	44 (0.1)
LMS vessel PCI		81 (2.0)	145 (1.9)	219 (2.1)	330 (3.0)	249 (1.9)	423 (1.9)	638 (2.1)	1097 (3.5)
Multivessel PCI		327 (8.1)	458 (6.0)	576 (5.5)	619 (5.7)	1066 (8.3)	1432 (6.4)	1831 (6.1)	2031 (6.5)

Table A3 Crude and relative survival estimates with 95% confidence limits

Year	30-days				365-days			
	Crude survival	Crude survival 95% CI	Relative survival	Relative survival 95% CI	Crude survival	Crude survival 95% CI	Relative survival	Relative survival 95% CI
**Female, Elective**								
**2007-2008**	0.996	(0.994, 0.997)	0.997	(0.996, 0.998)	0.977	(0.974, 0.979)	0.995	(0.992, 0.997)
**2009-2010**	0.996	(0.995, 0.997)	0.998	(0.997, 0.999)	0.979	(0.977, 0.981)	0.998	(0.996, 1.001)
**2011-2012**	0.995	(0.994, 0.996)	0.997	(0.995, 0.998)	0.973	(0.970, 0.976)	0.993	(0.990, 0.996)
**2013-2014**	0.995	(0.994, 0.997)	0.997	(0.996, 0.998)	0.975	(0.972, 0.978)	0.995	(0.992, 0.998)
**Female, UA/NSTEMI**								
**2007-2008**	0.983	(0.980, 0.985)	0.985	(0.982, 0.987)	0.946	(0.942, 0.950)	0.967	(0.963, 0.971)
**2009-2010**	0.980	(0.978, 0.983)	0.982	(0.980, 0.985)	0.939	(0.935, 0.943)	0.962	(0.957, 0.966)
**2011-2012**	0.977	(0.974, 0.979)	0.979	(0.977, 0.981)	0.937	(0.933, 0.941)	0.960	(0.956, 0.964)
**2013-2014**	0.978	(0.976, 0.980)	0.980	(0.978, 0.982)	0.937	(0.933, 0.941)	0.961	(0.957, 0.965)
**Female, STEMI**								
**2007-2008**	0.920	(0.912, 0.928)	0.922	(0.913, 0.930)	0.875	(0.864, 0.884)	0.895	(0.884, 0.905)
**2009-2010**	0.919	(0.913, 0.925)	0.921	(0.915, 0.927)	0.869	(0.861, 0.876)	0.891	(0.883, 0.899)
**2011-2012**	0.914	(0.908, 0.919)	0.916	(0.910, 0.921)	0.865	(0.858, 0.871)	0.888	(0.881, 0.895)
**2013-2014**	0.911	(0.905, 0.916)	0.913	(0.907, 0.918)	0.864	(0.857, 0.870)	0.886	(0.879, 0.893)
**Male, Elective**								
**2007-2008**	0.997	(0.997, 0.998)	0.999	(0.998, 0.999)	0.980	(0.978, 0.981)	1.000	(0.998, 1.001)
**2009-2010**	0.997	(0.996, 0.998)	0.999	(0.998, 0.999)	0.979	(0.977, 0.980)	0.999	(0.998, 1.001)
**2011-2012**	0.997	(0.996, 0.997)	0.999	(0.998, 0.999)	0.978	(0.977, 0.980)	0.999	(0.997, 1.000)
**2013-2014**	0.997	(0.996, 0.997)	0.998	(0.998, 0.999)	0.977	(0.975, 0.978)	0.998	(0.996, 0.999)
**Male, UA/NSTEMI**								
**2007-2008**	0.985	(0.984, 0.987)	0.987	(0.986, 0.988)	0.958	(0.956, 0.960)	0.978	(0.976, 0.980)
**2009-2010**	0.985	(0.984, 0.986)	0.987	(0.986, 0.988)	0.955	(0.953, 0.957)	0.976	(0.974, 0.978)
**2011-2012**	0.984	(0.982, 0.985)	0.986	(0.984, 0.987)	0.949	(0.947, 0.952)	0.971	(0.969, 0.973)
**2013-2014**	0.982	(0.981, 0.983)	0.984	(0.982, 0.985)	0.944	(0.941, 0.946)	0.966	(0.963, 0.968)
**Male, STEMI**								
**2007-2008**	0.947	(0.943, 0.951)	0.949	(0.945, 0.953)	0.920	(0.915, 0.924)	0.936	(0.931, 0.941)
**2009-2010**	0.951	(0.948, 0.954)	0.953	(0.950, 0.955)	0.922	(0.918, 0.925)	0.939	(0.935, 0.942)
**2011-2012**	0.947	(0.944, 0.949)	0.948	(0.945, 0.951)	0.916	(0.913, 0.919)	0.933	(0.930, 0.936)
**2013-2014**	0.942	(0.939, 0.944)	0.943	(0.941, 0.946)	0.913	(0.910, 0.916)	0.930	(0.926, 0.933)

Figure A1 Observed survival rates by patient age, indication, and year of procedure, with 95% confidence limits. P-values from the log-rank test.

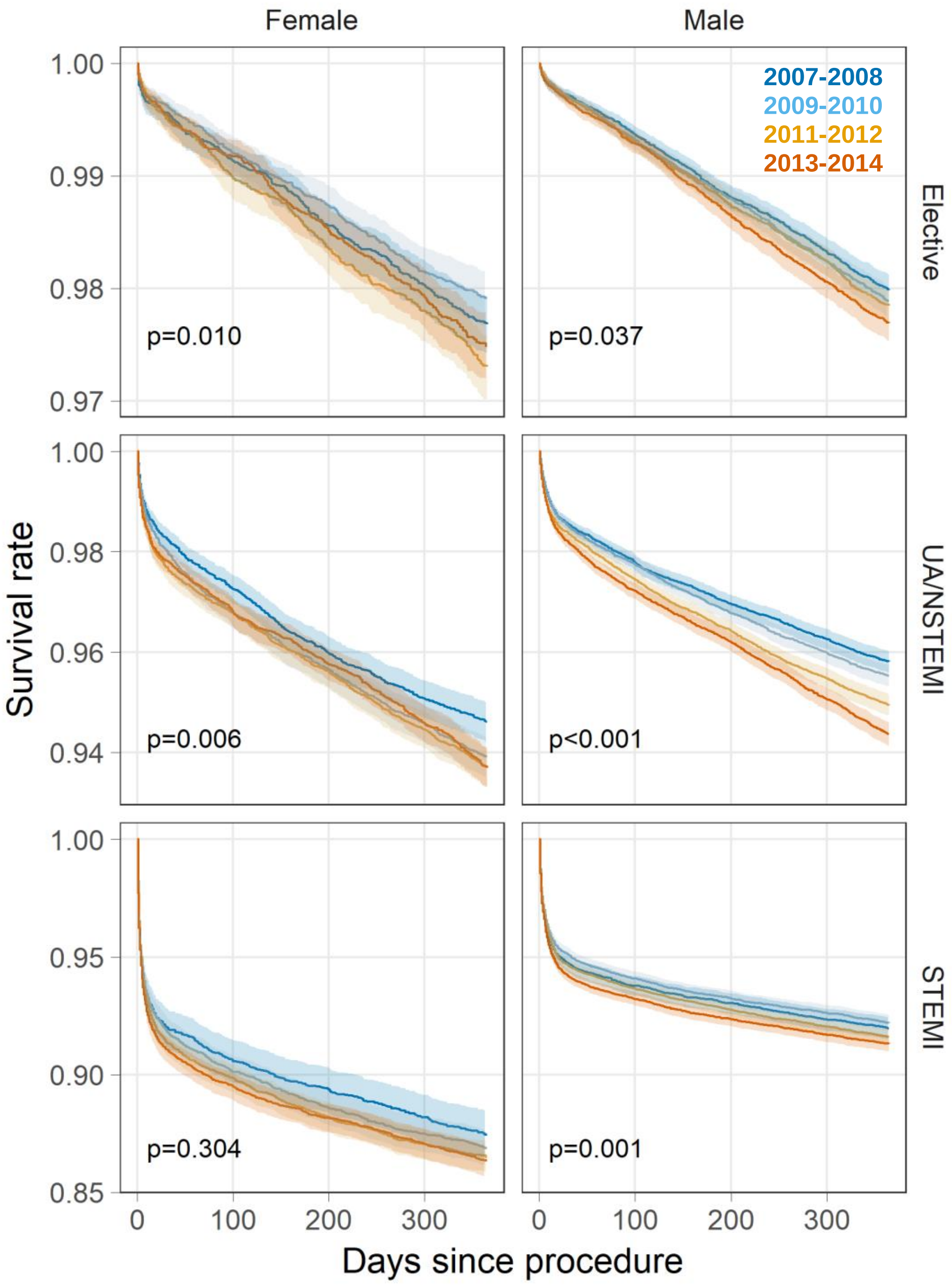

Figure A2 Relative survival estimates by patient age, indication, and year of procedure, with 95% confidence limits. P-values from a log-rank-type test for relative survival curves.

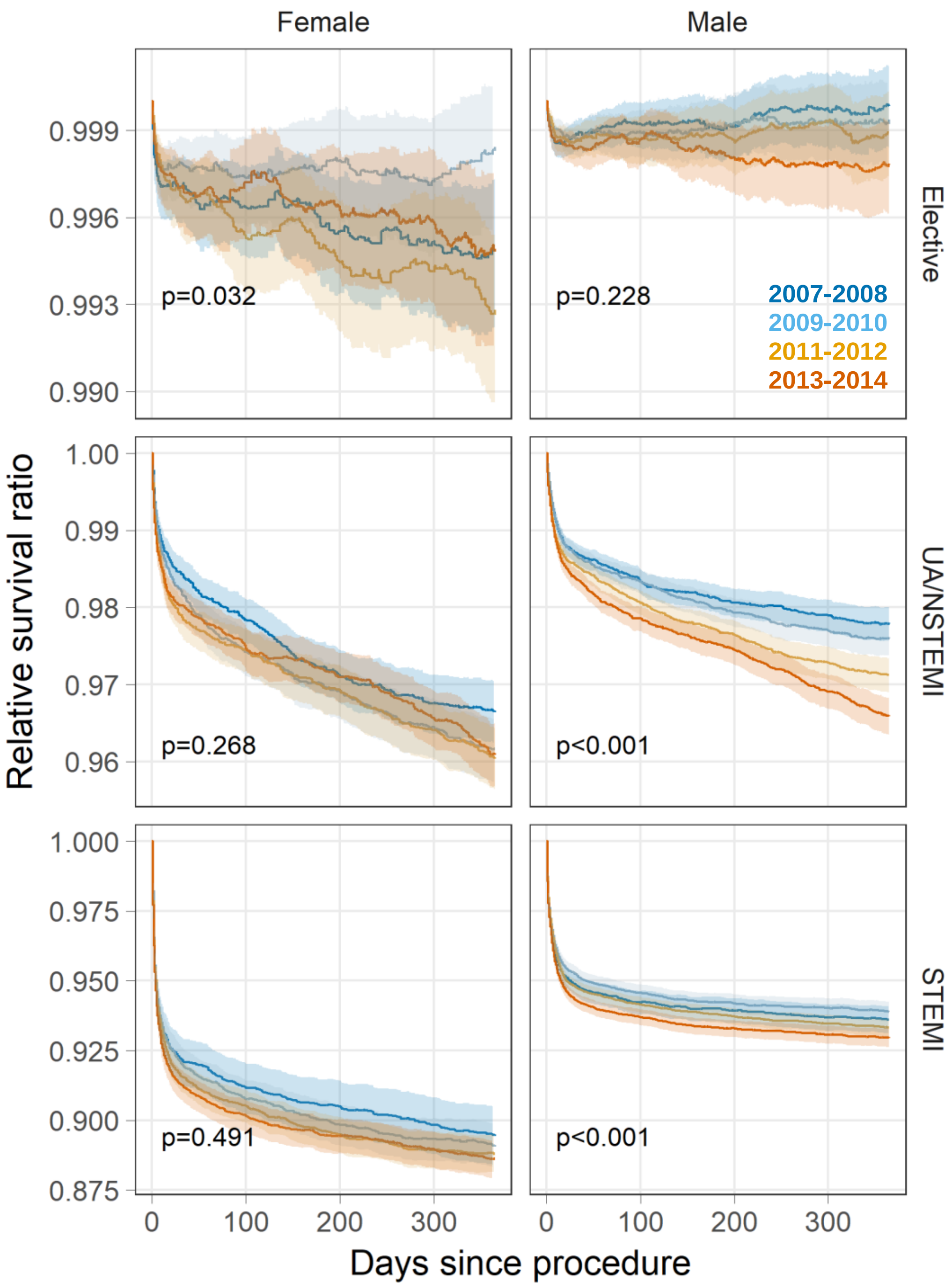

Figure A3 Observed-to-expected one-year mortality ratio (log-scale) by patient age, with 95% confidence limits.

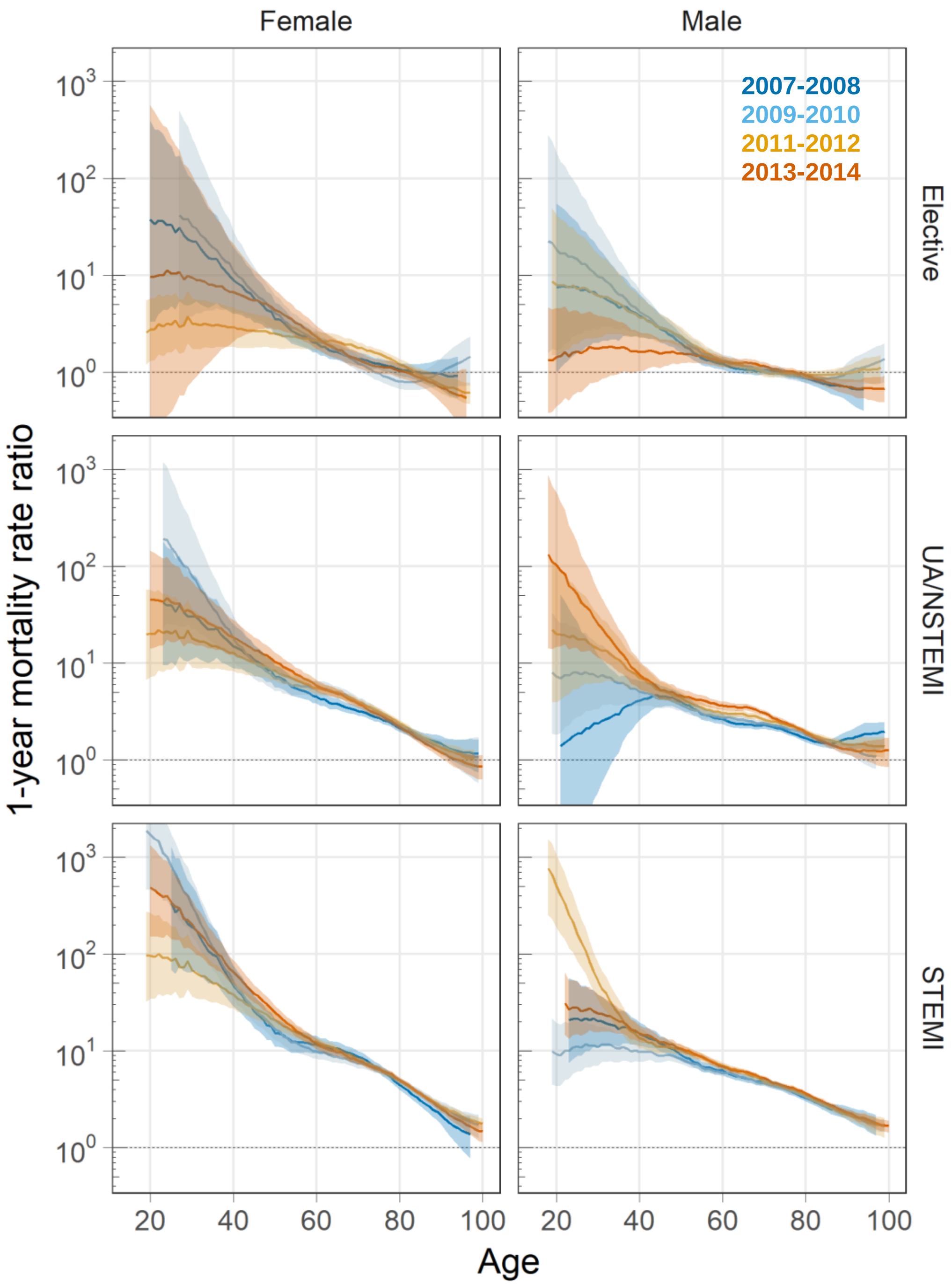

Binomial General Additive Models with 7-knot splines are used for smoothing the observed one-year mortality, then divided by the expected mortality rate. 95% confidence limits are removed for clarity.

Figure A4 Cumulative excess hazard rates by patient age, indication, and year of procedure, with 95% confidence limits.

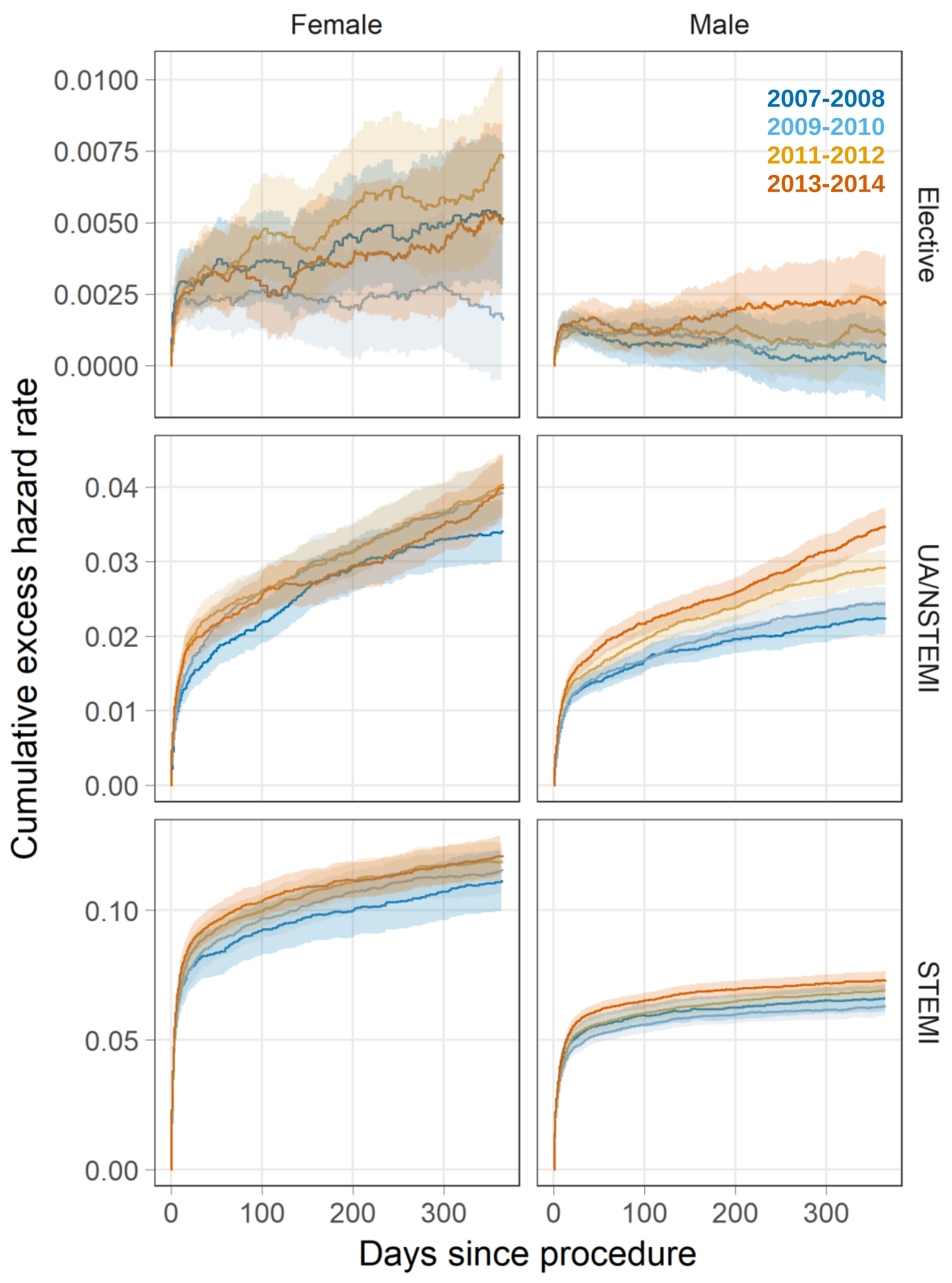

Supplement: Supplementary data [file bmjopen-2018-024627supp001.pdf]
