# Supplementary material for: Temporal trends in relative survival following percutaneous coronary intervention
Source: BMJ Open. 2019 Feb 9;9(2):e024627. doi: 10.1136/bmjopen-2018-024627 (PMC6398900; doi:10.1136/bmjopen-2018-024627)
Supplement: Author's manuscript [file bmjopen-2018-024627.draft_revisions.pdf]

BMJ Open is committed to open peer review. As part of this commitment we make the peer review history of every article we publish publicly available.

When an article is published we post the peer reviewers' comments and the authors' responses online. We also post the versions of the paper that were used during peer review. These are the versions that the peer review comments apply to.

The versions of the paper that follow are the versions that were submitted during the peer review process. They are not the versions of record or the final published versions. They should not be cited or distributed as the published version of this manuscript.

BMJ Open is an open access journal and the full, final, typeset and author-corrected version of record of the manuscript is available on our site with no access controls, subscription charges or pay-per-view fees (http://bmjopen.bmj.com).

If you have any questions on BMJ Open's open peer review process please email info.bmjopen@bmj.com

BMJ Open

Temporal trends in Relative Survival Following Percutaneous Coronary Intervention

Journal:	*BMJ Open*
Manuscript ID	bmjopen-2018-024627
Article Type:	Research
Date Submitted by the Author:	11-Jun-2018
Complete List of Authors:	Hulme, William; University of Manchester, Institute of Population Health<br>Sperrin, Matthew; University of Manchester Institute of Population Health,<br>Martin, Glen; University of Manchester Institute of Population Health, Farr Institute<br>Curzen, Nick; University Hospital Southampton NHS F Trust, Wessex Cardiac Unit<br>Ludman, Peter; Queen Elizabeth Hospital<br>Kontopantelis, Evangelos; University of Manchester, Faculty of Biology, Medicine and Health<br>Mamas, Mamas; University of Keele and Academic Department of Cardiology, Institute of Primary Care and Health Sciences
Keywords:	Coronary intervention < CARDIOLOGY, Quality in health care < HEALTH SERVICES ADMINISTRATION & MANAGEMENT, STATISTICS & RESEARCH METHODS

SCHOLARONE™
Manuscripts

For peer review only

Temporal trends in Relative Survival Following Percutaneous Coronary Intervention

Brief title: Relative survival after PCI

William J Hulme [1], Matthew Sperrin [1], Glen Martin [1], Nick Curzen [2], Peter Ludman [3], Evangelos Kontopantelis[1], Mamas A Mamas[4]; On behalf of the British Cardiovascular Intervention Society and the National Institute of Cardiovascular Outcomes Research.

1. Farr Institute, Faculty of Biology, Medicine and Health, University of Manchester, Manchester Academic Health Science Centre, Manchester, United Kingdom

2. University Hospital Southampton and Faculty of Medicine, University of Southampton, UK

3. Queen Elizabeth Hospital, Birmingham, UK.

4. Keele Cardiovascular Research Group, Centre for Prognosis Research, Institute of Primary Care and Health Sciences, University of Keele and Academic Department of Cardiology, Royal Stoke Hospital, Stoke-on-Trent, UK

Corresponding author:

William Hulme
Vaughan House
Portmouth Street
M13 9GB
Manchester, United Kingdom
Email: william.hulme@manchester.ac.uk

Word Count: 2927

For peer review only

ABSTRACT

Objective Percutaneous Coronary Intervention (PCI) has seen substantial shifts in patient selection in recent years that have increased baseline patient mortality risk. It is unclear to what extent observed changes in mortality are attributable to background mortality risk or the indication and selection for PCI itself. PCI-attributable mortality can be estimated using Relative Survival, which adjusts observed mortality by that seen in a matched control population. We report relative survival ratios and compare these across different time periods.

Methods National Health Service PCI activity in England and Wales from 2007 to 2014 is considered using data from the British Cardiovascular Intervention Society PCI Registry. Background mortality is as reported in Office for National Statistics life tables. Relative survival ratios up to one-year are estimated, matching on patient age, sex, and procedure date. Estimates are stratified by indication for PCI, sex, and procedure date.

Results 549,305 procedures were studied after exclusions for missing age, sex, indication, and mortality status. Comparing from 2007-08 to 2013-14, differences in crude survival at one-year were consistently lower in later years across all strata. For relative survival, these differences remained but were smaller, suggesting poorer survival in later years is partly due to demographic characteristics. Relative survival was higher in older patients.

Conclusions Changes in patient demographics account for some but not all of the crude survival changes seen during the study period. Relative survival is an under-used methodology in interventional settings like PCI and should be considered wherever survival is compared between populations with different demographic characteristics, such as between countries or time periods.

For peer review only

Strengths and limitations of this study

- Using national registry data, this study is the first to compare patient survival after Percutaneous Coronary Intervention with matched population survival across an entire healthcare system.
- It provides a template for similar investigations in other interventional settings where relative survival is under-used.
- Demographic matching in addition to age, sex, and year of procedure was not possible, though life-tables stratified by other characteristics may be available from the Office for National Statistics in future.

For peer review only

INTRODUCTION

The worldwide use of Percutaneous Coronary Intervention (PCI) for coronary revascularisation has expanded in recent years[1–6] though differences in this change have been observed internationally.[7,8] In the UK, the number of procedures increased from around 78,000 in 2007 to 97,000 in 2016, with the proportion of acute coronary syndrome (ACS) cases increasing from 53.6% to 64.3%[9] in the same period. In the USA, data from the CathPCI registry indicate an increase in the proportion of PCIs for ACS from 57.0% to 64.3% from 2011 to 2014.[3,10]

Correspondingly, patient case-mix has also changed such that PCI is now more likely to be performed in older, more co-morbid patients at higher risk of early mortality. For instance, in the UK between 2007 and 2015, mean patient age increased from 63.6 to 65.1 years and the proportion of patients with diabetes rose from 17.5% to 22.0%.[9] This is due, in part, to more permissive patient selection criteria and a move to more emergent indications where there is less scope for case selection and increased access,[11,12] though changes in the characteristics of the general population may partially explain this transition. Indeed, improvements in secondary prevention for cardiovascular disease and wider societal changes in general may be a significant driver of changes to baseline patient risk given a persistent, albeit slowing, decline in age-standardised cardiovascular mortality in both the UK[13] and the USA.[14] In addition, the evidence-base concerning optimal intervention techniques and treatment strategies has evolved, prompting changes to procedural practice towards the treatment of more complex disease.[15–17]

Mortality rates following PCI will be affected by these trends, though it is unclear to what extent the any changes are related to the baseline mortality risk of selected patients or procedural practice. Consequently, there is a need to report mortality rates from national PCI registries within the context of the changing PCI patient demographic, relative to corresponding changes in the background population.

Relative Survival is a statistical technique that compares the survival of a disease or treatment group with the survival of the general population matched on one or more characteristics, such as age and sex, for example as presented in national life-expectancy tables.[18,19] The excess mortality – that is, the mortality after adjusting for the expected mortality estimated from the control population – associated with that group can then be quantified. Relative Survival methods are typically used to study survival differences following disease diagnosis or to compare long-term treatment strategies where excess mortality develops slowly, for example in cancer.[20,21] More recently it is being

For peer review only

considered in cardiovascular settings,[22–24] including PCI,[25–29] though these have focused on long-term survival and did not consider changes over time.

We aimed to compare mortality rates following PCI with age- and sex-matched mortality rates found nationally, and to examine if these comparisons differ between early or contemporary cohorts.

METHODS

PCI procedure data

The British Cardiovascular Intervention Society (BCIS) audits all PCI activity in the United Kingdom, with data collection managed by the National Institute for Cardiovascular Outcomes Research (NICOR). Information on each procedure is captured locally by hospitals and uploaded to a central server. Mortality tracking is available via the Office for National Statistics (ONS) for procedures in England and Wales for patients with a valid NHS number. Mortality tracking was available up to May 2015.

Procedures in England and Wales between 2007 and 2014 for patients aged between 18 and 100 years old were extracted. Procedures with missing age, sex, indication, and mortality status were excluded.

ONS life-table data

Expected mortality rates for England and Wales are available from ONS national life-tables stratified by age in one-year bands, sex, and calendar year.[30]

Statistical analysis

Each procedure was matched to expected mortality life-table data based on the patients' age, sex, and the date of the procedure. Survival models were stratified by procedure date (2007-08, 2009-10, 2011-12, 2013-14), and by patient sex and indication (elective, UA/NSTEMI, STEMI) as these are known determinants of survival and there are distinct survival differences between these groups. Two-year time periods were chosen to ensure temporal trends are sufficiently captured while maintaining clarity of exposition. Analyses consider mortality up to one year post-procedure with mortality censored thereafter, as long-term estimates are confounded by the increasing influence of competing contributors to mortality and time-varying risk factors.[31]

For peer review only

Crude survival rates are plotted, with survival differences between periods compared using the log-rank test.[32,33] Relative survival models are built that adjust for expected survival using the Ederer II method.[18,19] Relative survival estimates are plotted, and differences between periods compared using the log-rank test after a transformation of survival time described by Stare, Henderson and Pohar.[34] The observed (PCI) and expected (life-table) one-year mortality rates are also compared by considering their ratios across different ages. This expresses the relationship of relative survival and age without explicitly modelling the hazard function, which is challenging in this setting since the hazard falls extremely steeply in the early stages of follow up. This quantity is plotted, using General Additive Models with a binomial link to smooth observed mortality.

The relative survival estimate, calculable at each day post-procedure, is the ratio of the observed survival proportion of those who had PCI to the expected survival proportion based on a matched population. This quantity can interpreted as the post-PCI survival rate if patients were only exposed to PCI-related mortality risk and not other background risks. PCI-related mortality risk should be understood as the mortality risk of being a person who had PCI, not simply the risk of the procedure itself. If the relative survival estimate levels-off after a given time then, on average, there is no additional PCI-related mortality risk for those who survived up to that time. If the relative survival estimate increases then, on average, mortality was less than expected had the patients not needed PCI. In most interventional settings this quantity is typically less than one, indicating that PCI is associated with a greater mortality risk than no PCI.

All analyses were performed using R version 3.4.4.[35] The tidyverse data manipulation and visualization suite[36] was used throughout. The relsurv package[37–39] was used for relative survival modelling. The complete R script is available on GitHub,[40] with synthetic BCIS data provided to replicate analysis steps without disclosing proprietary BCIS data. Access to study materials was only required for the first author.

Patient involvement

This is a registry-based study with all data collected prior to the design phase and outcomes necessarily restricted to mortality only. Therefore, patients were not involved in the design of this study.

For peer review only

RESULTS

In total, there were 575,203 procedures recorded in the BCIS-NICOR registry between 2007 and 2014 in England and Wales, of which 549,305 (95.5%) were available for analysis after exclusions due to missing data (see Supplementary Table A1). Follow-up is complete for every procedure up to one-year (i.e., no censoring occurred before one year), except for procedures from June to December 2014, where available follow-up time was less than one year.

Table 1 presents procedure numbers by year, sex, and indication, and Supplementary Tables A2a, A2b, and A2c present baseline patient characteristics and mortality rates for elective, UA/NSTEMI, and STEMI procedures, respectively. Female patients made up a quarter (25.9%) of procedures. Mean patient age increased from 63.8 years old in 2007-08 to 65.1 years old in 2013-14, with similar increases observed within indications (64.5 to 65.8 years for elective procedures; 63.6 to 65.7 years for UA/NSTEMI; 62.1 to 63.5 years for STEMI). PCI for ACS (UA/NSTEMI/STEMI) accounted for 61.5% of procedures though this proportion increased from 54.0% to 65.7%.

Figure 1 presents the observed survival rates without adjustment for expected survival. Across all indications mortality risk is highest immediately following the procedures as this is where the survival curve gradient is steepest. The log-rank test indicates some differences in crude survival rates between periods and, typically, more recent years are associated with lower survival. Survival differences by indication and sex are also evident, with survival consistently lower in women. Comparing crude survival from 2007-08 to 2013-14 at one-year: elective procedures, 97.7% to 97.5% for women and 98.0% versus 97.7% for men; UA/STEMI, 94.6% versus 93.7% for women and 95.8% versus 94.4% for men; STEMI, 87.5% versus 86.4% for women and 92.0% versus 91.3% for men (see supplementary Table 2).

Figure 2 shows the relative survival estimate. For elective procedures relative survival levels-off quickly, indicating that procedural mortality risk is present immediately following the procedure but that on average, risk returns to the matched population baseline. For ACS procedures, relative survival continues to decline up to one-year post-procedure, indicating long-term excess risk in these patients compared with the matched population. The log-rank test for relative survival differences reveals smaller differences than for crude survival, though there remains strong evidence of survival differences for men following PCI for UA/STEMI or STEMI. Comparing relative survival from 2007-08 to 2013-14 at one-year: elective procedures, 99.5% to 99.5% for women and 100% versus 99.8% for men; UA/STEMI, 96.7% versus 96.1% for women and 97.8% versus 96.6% for men;

For peer review only

STEMI, 89.5% versus 88.6% for women and 93.6% versus 93.0% for men (see supplementary Table A3).

Figure 3 shows the ratio between observed and expected one-year mortality rates by patient age. For younger patients, observed mortality is considerably higher than expected mortality, but this gap reduces with patient age. For instance, observed mortality at one year following elective PCI is 2-5 times greater than expected at age 50 but these are equal by age 80. For UA/NSTEMI, this ratio is between 5-10 at age 50, though is less than 3 by age 80. For STEMI, this ratio decreases from around 9-15 to around 5 from age 50 to 80. There are no clear trends over time.

Confidence intervals at the 95% level for Figures 1, 2, and 3 are provided in supplementary materials (Supplementary Figures A1, A2, A3), as are plots of the cumulative excess hazard rate (Supplementary Table A4), which may be interpreted as the total additional exposure to mortality risk at any point in time for PCI patients compared with the general population.

Table 1 Number of procedures by period, sex, indication

	2007-2008		2009-2010		2011-2012		2013-2014		2007-2014	
	N	%	N	%	N	%	N	%	N	%
**Female**										
Elective	13847	*45.2*	13392	*38.4*	12603	*33.0*	12093	*31.3*	51935	*36.5*
UA/NSTEMI	12756	*41.6*	13858	*39.8*	15139	*39.6*	15594	*40.4*	57347	*40.3*
STEMI	4043	*13.2*	7608	*21.8*	10480	*27.4*	10908	*28.3*	33039	*23.2*
Total	30646		34858		38222		38595		142321	
**Male**										
Elective	40246	*46.3*	40284	*40.4*	39715	*36.3*	39228	*35.3*	159473	*39.2*
UA/NSTEMI	33797	*38.9*	37049	*37.2*	39379	*36.0*	40749	*36.7*	150974	*37.1*
STEMI	12921	*14.9*	22306	*22.4*	30244	*27.7*	31066	*28.0*	96537	*23.7*
Total	86964		99639		109338		111043		406984	
**Male and Female**										
Elective	54093	*46.0*	53676	*39.9*	52318	*35.5*	51321	*34.3*	211408	*38.5*
UA/NSTEMI	46505	*39.6*	50745	*37.8*	54314	*36.9*	56152	*37.7*	208321	*37.9*
STEMI	16964	*14.4*	29914	*22.2*	40724	*27.6*	41974	*28.1*	129576	*23.6*
Total	117610		134497		147560		149638		549305	

For peer review only

For peer review only

DISCUSSION

We present results for 549,305 PCI procedures over an 8-year period showing observed, expected, and relative survival estimates over time. We demonstrate that, after adjustment for patient age, sex, and procedure date using national life-table mortality rates, differences in patient survival over time are present in men following PCI for ACS, though these differences are small (relative survival at one-year: UA/NSTEMI 97.8% in 2007-2008 vs 96.6% in 2013-2014; STEMI 93.6% in 2007-2008 vs 93.0% in 2013-2014). Small, non-significant relative survival differences were found within other strata. However, the potential counteracting effects of selection factors (that may have increased mortality risk) and of improvements in processes of care (that may have reduced mortality risk) cannot be ruled out.

For elective cases, there is evidence that relative survival increases after the initial post-procedural risk. This suggests that, conditional on surviving the initial risk shock, mortality is on average lower than in the matched population. This is likely driven by patient selection factors, particularly for more elderly patients, where there may be selection bias such that PCI is undertaken in only the healthiest patients. Another factor may be that these patients are highly likely to be supported with secondary prevention treatments for coronary artery disease, whereas in the general population there may be many people with undetected or untreated CAD.

Mortality increases with age at a rate that is lower in the PCI population than in an equivalent matched population. In other words, though mortality is naturally higher as age increases, older patients treated by PCI have a mortality rate more similar to the rate in the general population than younger patients. This is true across all indications. In general then, relative survival is higher for older patients compared with younger patients, though in absolute terms their prognosis is still poorer.

This may partly be determined by clinician selection practices, such that younger patients with CAD are more likely to be referred for PCI than older patients for whom PCI is not optimal. For instance, for elective procedures, any 80-year-old considered well enough to undergo elective PCI is likely to be otherwise healthy and without the typical contraindications for PCI such as intolerance of anti-platelet therapies or other significant co-morbidities and frailties. Conversely, a 40-year-old requiring elective PCI clearly has a higher baseline risk for mortality than the average 40-year-old. However, epidemiological selection processes are also relevant. Irrespective of the decision to treat CAD with PCI, younger PCI patients are sicker than older PCI patients relative to a matched population, given

For peer review only

that competing mortality risks loom larger in an older population where death from other causes such as cancer or respiratory illnesses is more likely. Finally, considering urgent or emergency procedures, simply being part of the PCI registry suggests a degree of patient resilience having experienced an acute event but survived long enough to be treated. Due to increasing frailty, older patients should expect inferior odds than younger patients of receiving PCI before dying, so those older patients that do survive to PCI will typically be less frail than others of a similar age. The distinct trend for higher relative survival in older patients is thus clear evidence of these selection processes at play, and the importance of accounting for these imbalances when analysing registry data.

Across all indications, both absolute and relative survival is lower in women than in men. This is a well-known phenomenon in cardiovascular settings variously attributed to differences in age, co-morbidities, time to treatment, secondary prevention.[41–44] This work demonstrates unequivocally that differences in age and background mortality rates do not sufficiently account for this disparity.

In general, both crude and relative survival estimates are lower in recent years. Assuming a consistent data collection methodology over time, realistically this can either be attributed to an average reduction in procedural efficacy or an increase in higher risk procedures that is not solely explained by age- and sex-adjusted background mortality. In the context of rapid changes to PCI patient case-mix and practice in recent years,[45] including more permissive patient selection criteria and increasing use of PCI in favour of alternative revascularisation strategies, the small decline in relative survival observed from 2007 to 2014 is unsurprising. Further adjustment for patient case-mix will demonstrate if and where actual survival gains have been made, or indeed if survival in certain groups of patients has not improved over time.

Relative survival offers distinct advantages over crude survival rates.[19] The relative mortality estimate (one minus relative survival) may be interpreted as an approximation of the excess mortality attributable jointly to the indication for intervention and the intervention itself. This allows PCI-specific mortality to be measured without needing cause of death information which may often be inconsistently recorded or unavailable completely. Relative survival also neatly accounts for mortality that may be only indirectly related to PCI, for instance non-cardiac mortality secondary to post-operative bleeding.

As the underlying population may vary considerably across different countries, intervention registries or time periods, methods that appropriately adjust for background population mortality

For peer review only

provide more suitable survival estimates for comparison in time and space than crude survival alone, provided the comparisons are valid.[46,47] However, despite the popularity of relative survival methodology to compare survival in diseased versus non-diseased populations, in particular cancer, these methods have only recently seen some use in interventional settings like PCI, and relative survival is not routinely reported by PCI registries. This study concisely demonstrates the feasibility and appropriateness of the relative survival approach in such settings and provides a useful template for producing population-adjusted PCI survival estimates in the future.

Limitations

Patients are matched to national mortality estimates by year, age, and sex only. Despite stratification of models by these factors, substantial case-heterogeneity will remain that is not modelled. No account is taken for other potentially unbalanced factors such as ethnicity, social-economic status, and comorbidities. Accounting for these factors is not possible with the simple relative survival approach used here as national life-tables stratifying by these factors are not readily available. However, using regression modelling in this setting is challenging as the mortality risk following PCI is raised acutely and the hazard rate changes rapidly; modelling the hazard must be done with extreme care as many assumptions of standard regression techniques are unlikely to hold.

Patients will be present more than once in the PCI cohorts if they have been re-admitted for subsequent PCI within the study period. The proportion of procedures in patients already present in the cohort is 13.5% though this is reduced to 7.3% when considering indications separately.

Life-table data is based on national mortality statistics and therefore is not strictly independent of the PCI registry, since all patients will contribute to population and mortality statistics. This is an unavoidable complication in the absence of stratification by CAD or PCI status that biases results towards no relative survival difference. However, given the analysis cohort constitutes less than one percent of the population of England and Wales, this bias can be considered negligible.

As the log-rank tests stratify groups independently it will ignore the inherent correlation between adjacent or near-adjacent years. It does not provide point-estimates of differences between strata as all differences through time contribute to the test statistic. It is therefore most useful when used alongside a graphical representation of survival differences.

For peer review only

Conclusion

We demonstrate that the decline in indication-stratified crude survival rate over time is less apparent after adjusting for expected population mortality matched on age, sex, and procedure year, though patient demographics do not explain all of the difference. Adjusting for additional patient risk factors is necessary to identify other drivers of mortality change.

For peer review only

References

1 Ludman PF. BCIS audit report for 2015 Activity. 2016.https://www.bcis.org.uk/wp-content/uploads/2017/10/BCIS-Audit-2015-data-for-web-with-presentation-ACI-2017-19-10-2017.pdf

2 Desai NR, Bradley SM, Parzynski CS, *et al.* Appropriate Use Criteria for Coronary Revascularization and Trends in Utilization, Patient Selection, and Appropriateness of Percutaneous Coronary Intervention. *JAMA* 2015;**314**:2045. doi:10.1001/jama.2015.13764

3 Masoudi FA, Ponirakis A, de Lemos JA, *et al.* Trends in U.S. Cardiovascular Care: 2016 Report From 4 ACC National Cardiovascular Data Registries. Journal of the American College of Cardiology. 2017;**69**:1427–50. doi:10.1016/j.jacc.2016.12.005

4 Jiménez-Quevedo P, Serrador A, Pérez de Prado A, *et al.* Spanish Cardiac Catheterization and Coronary Intervention Registry. 25th Official Report of the Spanish Society of Cardiology Working Group on Cardiac Catheterization and Interventional Cardiology (1990-2015). *Revista espanola de cardiologia (English ed)* 2016;**69**:1180–9. doi:10.1016/j.rec.2016.08.016

5 Pereira H, Campante Teles R, Costa M, *et al.* Evolução da intervenção coronária percutânea entre 2004□2013. Atividade em Portugal segundo o Registo Nacional de Cardiologia de Intervenção. *Revista Portuguesa de Cardiologia* 2015;**34**:673–81. doi:10.1016/j.repc.2015.06.005

6 Ohlmeier C, Czwikla J, Enders D, *et al.* Perkutane koronare Interventionen. *Bundesgesundheitsblatt - Gesundheitsforschung - Gesundheitsschutz* 2016;**59**:783–8. doi:10.1007/s00103-016-2352-5

7 Laut KG, Gale CP, Pedersen AB, *et al.* Persistent geographical disparities in the use of primary percutaneous coronary intervention in 120 European regions: exploring the variation. *EuroIntervention : journal of EuroPCR in collaboration with the Working Group on Interventional Cardiology of the European Society of Cardiology* 2013;**9**:469–76. doi:10.4244/EIJV9I4A76

8 Smith FG, Brogan RA, Alabas O, *et al.* Comparative care and outcomes for acute coronary syndromes in Central and Eastern European Transitional countries: A review of the literature. *European Heart Journal: Acute Cardiovascular Care* 2015;**4**:537–54.

For peer review only

doi:10.1177/2048872614551545

9 National Institute of Cardiovascular Outcomes Research. Annual reports: findings from the National Audit of Percutaneous Coronary Interventions. 2016.http://www.ucl.ac.uk/nicor/audits/adultpercutaneous/reports (accessed 1 Jan 2017).

10 Masoudi FA, Ponirakis A, Yeh RW, *et al.* Cardiovascular care facts: A report from the national cardiovascular data registry: 2011. *Journal of the American College of Cardiology* 2013;**62**:1931–47. doi:10.1016/j.jacc.2013.05.099

11 National Institute for Clinincal Outcomes Research, Herrett E, Smeeth L, *et al.* Myocardial Ischaemia National Audit Project. Annual Public Report April 2014 - March 2015. *Heart (British Cardiac Society)* 2015;**96**:1264–7. doi:10.1136/hrt.2009.192328

12 Hall M, Laut K, Dondo TB, *et al.* Patient and hospital determinants of primary percutaneous coronary intervention in England, 2003–2013. *Heart* 2016;**102**:313–9. doi:10.1136/heartjnl-2015-308616

13 Bhatnagar P, Wickramasinghe K, Wilkins E, *et al.* Trends in the epidemiology of cardiovascular disease in the UK. *Heart* 2016;**102**:1945–52. doi:10.1136/heartjnl-2016-309573

14 Benjamin EJ, Blaha MJ, Chiuve SE, *et al.* Heart Disease and Stroke Statistics'2017 Update: A Report from the American Heart Association. Circulation. 2017;**135**:e146–603. doi:10.1161/CIR.0000000000000485

15 Banning AP, Baumbach A, Blackman D, *et al.* Percutaneous coronary intervention in the UK: recommendations for good practice 2015. *Heart* 2015;**101**:1–13. doi:10.1136/heartjnl-2015-307821

16 Harold JG, Bass TA, Bashore TM, *et al.* ACCF/AHA/SCAI 2013 Update of the Clinical Competence Statement on Coronary Artery Interventional Procedures. *Catheterization and Cardiovascular Interventions* 2013;**82**:E69–111. doi:10.1002/ccd.24985

17 Authors/Task Force members, Windecker S, Kolh P, *et al.* 2014 ESC/EACTS Guidelines on myocardial revascularization. *European Heart Journal* 2014;**35**:2541–619. doi:10.1093/eurheartj/ehu278

For peer review only

18 Ederer F, Axtell LM, Cutler SJ. The relative survival rate: a statistical methodology. *National Cancer Institute monograph* 1961;**6**:101–21.http://www.ncbi.nlm.nih.gov/pubmed/13889176 (accessed 16 Oct 2017).

19 Lambert PC, Dickman PW, Rutherford MJ. Comparison of different approaches to estimating age standardized net survival. *BMC Medical Research Methodology* 2015;**15**:64. doi:10.1186/s12874-015-0057-3

20 Quaresma M, Coleman MP, Rachet B. 40-year trends in an index of survival for all cancers combined and survival adjusted for age and sex for each cancer in England and Wales, 1971–2011: a population-based study. *The Lancet* 2015;**385**:1206–18. doi:10.1016/S0140-6736(14)61396-9

21 Sarfati D, Blakely T, Pearce N. Measuring cancer survival in populations: Relative survival vs cancer-specific survival. *International Journal of Epidemiology* 2010;**39**:598–610. doi:10.1093/ije/dyp392

22 Nelson CP, Lambert PC, Squire IB, *et al.* Relative survival: what can cardiovascular disease learn from cancer? *European Heart Journal* 2008;**29**:941–7. doi:10.1093/eurheartj/ehn079

23 Martin GP, Sperrin M, Hulme W, *et al.* Relative Survival After Transcatheter Aortic Valve Implantation: How Do Patients Undergoing Transcatheter Aortic Valve Implantation Fare Relative to the General Population? *Journal of the American Heart Association* 2017;**6**:e007229. doi:10.1161/JAHA.117.007229

24 Hall M, Alabas OA, Dondo TB, *et al.* Use of relative survival to evaluate non-ST-elevation myocardial infarction quality of care and clinical outcomes. *European Heart Journal - Quality of Care and Clinical Outcomes* 2015;**1**:85–91. doi:10.1093/ehjqcco/qcv011

25 Velders MA, James SK, Libungan B, *et al.* Prognosis of elderly patients with ST-elevation myocardial infarction treated with primary percutaneous coronary intervention in 2001 to 2011: A report from the Swedish Coronary Angiography and Angioplasty Registry (SCAAR) registry. *American Heart Journal* 2014;**167**:666–73. doi:10.1016/j.ahj.2014.01.013

26 Alabas OA, Brogan RA, Hall M, *et al.* Determinants of excess mortality following unprotected left main stem percutaneous coronary intervention. *Heart* 2016;**102**:1287–95. doi:10.1136/heartjnl-2015-308739

For peer review only

27 Roth C, Gangl C, Dalos D, *et al.* Outcome after elective percutaneous coronary intervention depends on age in patients with stable coronary artery disease - An Analysis of relative survival in a multicenter cohort and an OCT substudy. *PLoS ONE* 2016;**11**. doi:10.1371/journal.pone.0154025

28 Brogan RA, Alabas O, Almudarra S, *et al.* Relative survival and excess mortality following primary percutaneous coronary intervention for ST-elevation myocardial infarction. *European heart journal Acute cardiovascular care* 2017;:2048872617710790. doi:10.1177/2048872617710790

29 Baart SJ, Van Domburg RT, Janssen-Heijnen MLG, *et al.* Impact of Relative Conditional Survival Estimates on Patient Prognosis after Percutaneous Coronary Intervention. *Circulation: Cardiovascular Quality and Outcomes* 2017;**10**. doi:10.1161/CIRCOUTCOMES.116.003344

30 Office for National Statistics. National Life Tables, England & Wales, 1980-82 to 2014-16. 2017.https://www.ons.gov.uk/peoplepopulationandcommunity/birthsdeathsandmarriages/lifeexpectancies/datasets/nationallifetablesenglandandwalesreferencetables (accessed 10 Oct 2017).

31 Dekker FW, de Mutsert R, van Dijk PC, *et al.* Survival analysis: time-dependent effects and time-varying risk factors. *Kidney International* 2008;**74**:994–7. doi:10.1038/ki.2008.328

32 Mantel N. Evaluation of survival data and two new rank order statistics arising in its consideration. *Cancer chemotherapy reports* 1966;**50**:163–70.http://www.ncbi.nlm.nih.gov/pubmed/5910392 (accessed 16 Oct 2017).

33 Peto R, Peto J. Asymptotically Efficient Rank Invariant Test Procedures. *Journal of the Royal Statistical Society Series A (General)* 1972;**135**:185. doi:10.2307/2344317

34 Stare J, Henderson R, Pohar M. An individual measure of relative survival. *Journal of the Royal Statistical Society: Series C (Applied Statistics)* 2005;**54**:115–26. doi:10.1111/j.1467-9876.2005.00473.x

35 R Development Core Team. R: a language and environment for statistical computing. [2.12.2]. 2011.http://www.r-project.org/.

For peer review only

36 Wickham H. Easily Install and Load 'Tidyverse' Packages [R package tidyverse version 1.1.1]. https://cran.r-project.org/web/packages/tidyverse/index.html (accessed 21 Mar 2017).

37 Perme MP. relsurv: Relative Survival. 2017.https://cran.r-project.org/package=relsurv

38 Pohar M, Stare J. Making relative survival analysis relatively easy. *Computers in Biology and Medicine* 2007;**37**:1741–9. doi:10.1016/j.compbiomed.2007.04.010

39 Pohar M, Stare J. Relative survival analysis in R. *Computer Methods and Programs in Biomedicine* 2006;**81**:272–8. doi:10.1016/j.cmpb.2006.01.004

40 Hulme W. BCIS-PCI-relative-survival GitHub repository. https://github.com/wjchulme/BCIS-PCI-relative-survival (accessed 4 May 2018).

41 Heer T, Hochadel M, Schmidt K, *et al.* Sex Differences in Percutaneous Coronary Intervention-Insights From the Coronary Angiography and PCI Registry of the German Society of Cardiology. *Journal of the American Heart Association* 2017;**6**:e004972. doi:10.1161/JAHA.116.004972

42 Raphael CE, Singh M, Bell M, *et al.* Sex Differences in Long-Term Cause-Specific Mortality After Percutaneous Coronary Intervention: Temporal Trends and Mechanisms. *Circulation Cardiovascular interventions* 2018;**11**:e006062. doi:10.1161/CIRCINTERVENTIONS.117.006062

43 Kunadian V, Qiu W, Lagerqvist B, *et al.* Gender Differences in Outcomes and Predictors of All-Cause Mortality After Percutaneous Coronary Intervention (Data from United Kingdom and Sweden). *American Journal of Cardiology* 2017;**119**:210–6. doi:10.1016/j.amjcard.2016.09.052

44 Birkemeyer RG, Schneider H, Rillig A, *et al.* Do gender differences in primary PCI mortality represent a different use of guideline adherent therapy? *European Heart Journal* 2013;**34**:P2227–P2227. doi:10.1093/eurheartj/eht308.P2227

45 Ludman P. BCIS Audit Report for 2014 Activity. 2015.https://www.bcis.org.uk/wp-content/uploads/2017/01/BCIS-audit-2014.pdf

46 Pokhrel A, Hakulinen T. How to interpret the relative survival ratios of cancer patients. *European Journal of Cancer* 2008;**44**:2661–7. doi:10.1016/j.ejca.2008.08.016

For peer review only

47 Rutherford MJ, Dickman PW, Lambert PC. Comparison of methods for calculating relative survival in population-based studies. *Cancer Epidemiology* 2012;**36**:16–21. doi:10.1016/j.canep.2011.05.010

For peer review only

Author Statement WH and MM conceived the study. WH, GM, MS, and EK participated in the design of the study. WH performed the statistical analysis and drafted the manuscript. All authors interpreted the results and made important contributions and revisions to the work. All authors read and approved the final manuscript.

Data sharing Analysis scripts and a synthetic test dataset are available on GitHub: https://zenodo.org/badge/latestdoi/131696652.

Funding This work was supported by MRC grant number MR/K006665/1.

Disclosures None.

For peer review only

Figure legends

Figure 1 Observed survival rates by patient indication, and year of procedure. P-values are from the log-rank test.

Figure 2 Relative survival estimates by patient indication, and year of procedure. P-values are from a log-rank-type test for relative survival curves.

Figure 3 Observed-to-expected one-year mortality ratio (log-scale) by patient age. Binomial General Additive Models with 7-knot splines are used for smoothing the observed one-year mortality, then divided by the expected mortality rate. 95% confidence limits are removed for clarity.

For peer review only

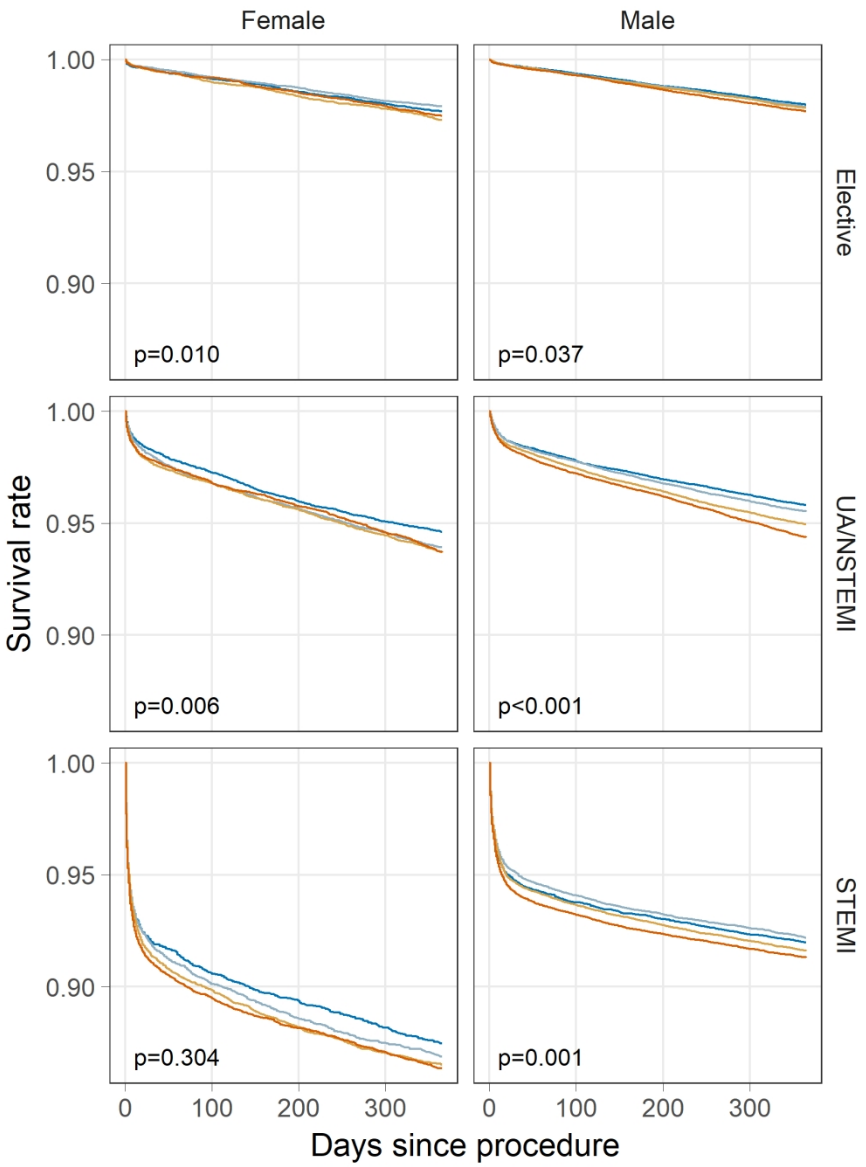

Observed survival rates by patient indication, and year of procedure. P-values are from the log-rank test.

149x199mm (300 x 300 DPI)

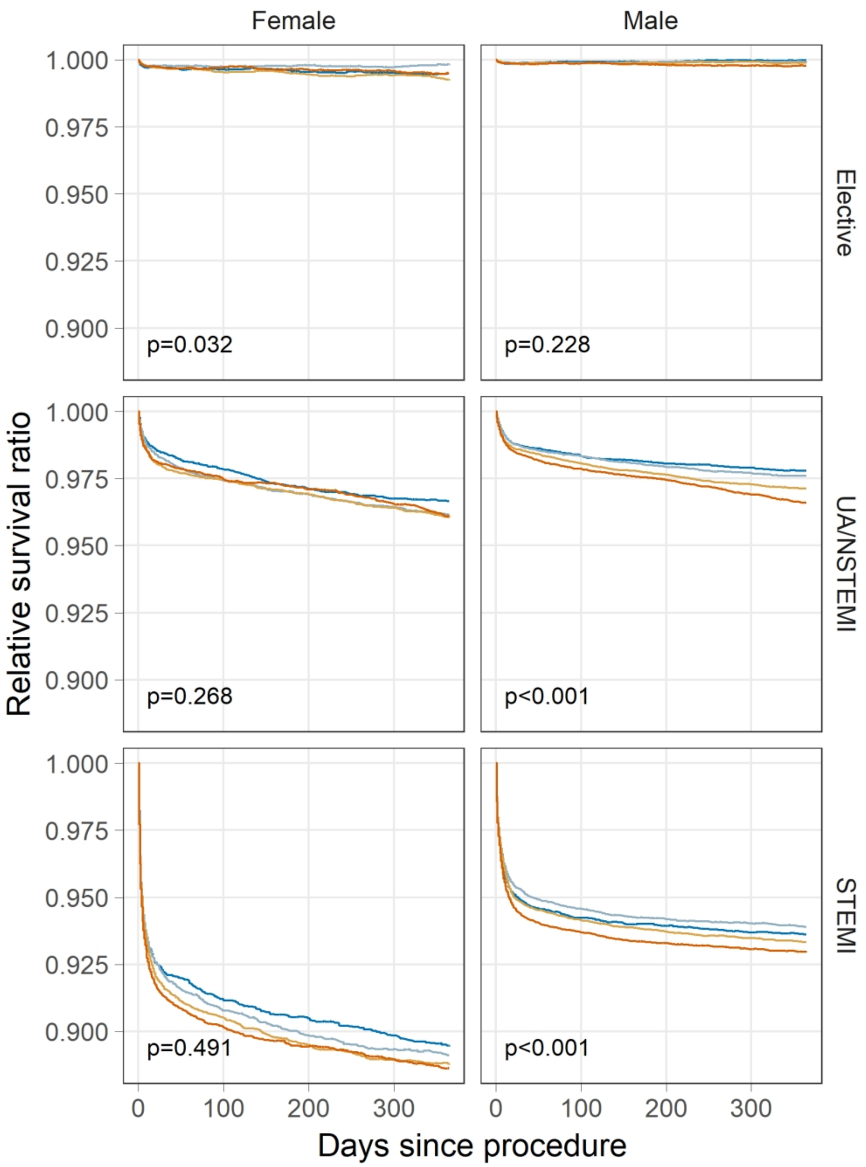

Relative survival estimates by patient indication, and year of procedure. P-values are from a log-rank-type test for relative survival curves.

149x199mm (300 x 300 DPI)

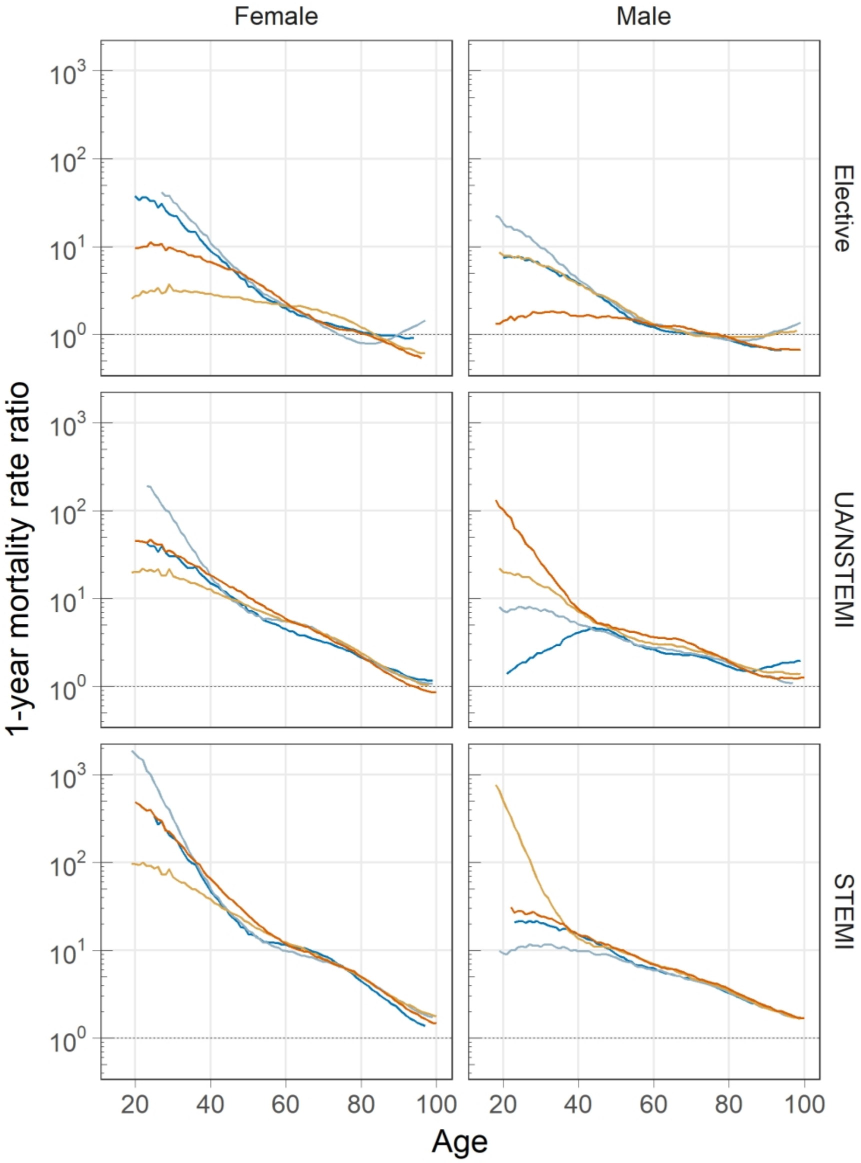

Observed-to-expected one-year mortality ratio (log-scale) by patient age.

149x199mm (300 x 300 DPI)

Supplementary Materials

Table A1 Procedure selection

	Procedures excluded		Procedures remaining	
	**N**	**Cumul. N**	**N**	**%**
Interventional procedures in England and Wales NHS, 2007 – 2014	-	-	575203	100.0
Exclude missing age and age<18 and age>100	439	439	574764	99.9
Exclude missing sex	984	1423	573780	99.8
Exclude missing indication and rescue/bail-out PCI	3887	5310	569893	99.1
Exclude missing mortality	20588	25898	549305	95.5

For peer review only

Table A2a Baseline patient characteristics for Elective procedures

		Female				Male			
		2007-2008	2009-2010	2011-2012	2013-2014	2007-2008	2009-2010	2011-2012	2013-2014
**Number of procedures**		13847	13392	12603	12093	40246	40284	39715	39228
**Mean age, years (SD)**		67.1 (10.0)	67.8 (10.3)	68.3 (10.5)	68.5 (10.6)	63.7 (10.2)	64.2 (10.5)	64.6 (10.6)	64.9 (10.8)
**Variable, N (%)**									
Age	<50	796 (5.7)	738 (5.5)	636 (5.0)	624 (5.2)	3723 (9.3)	3702 (9.2)	3451 (8.7)	3325 (8.5)
	50s	2229 (16.1)	2034 (15.2)	1940 (15.4)	1854 (15.3)	9940 (24.7)	9289 (23.1)	9021 (22.7)	8961 (22.8)
	60s	4641 (33.5)	4193 (31.3)	3871 (30.7)	3563 (29.5)	14270 (35.5)	14097 (35.0)	13734 (34.6)	13249 (33.8)
	70s	4942 (35.7)	4820 (36.0)	4364 (34.6)	4235 (35.0)	10197 (25.3)	10486 (26.0)	10354 (26.1)	10191 (26.0)
	80+	1239 (8.9)	1607 (12.0)	1792 (14.2)	1817 (15.0)	2116 (5.3)	2710 (6.7)	3155 (7.9)	3502 (8.9)
Ethnicity	Asian	482 (5.1)	628 (6.0)	614 (6.2)	614 (6.4)	1777 (6.6)	2127 (6.8)	2329 (7.4)	2256 (7.3)
	Black	80 (0.9)	90 (0.9)	108 (1.1)	100 (1.0)	153 (0.6)	201 (0.6)	190 (0.6)	228 (0.7)
	White	7881 (84.2)	9216 (87.3)	8463 (85.2)	8045 (83.7)	22368 (82.5)	27231 (86.5)	26526 (84.3)	25584 (82.7)
	Other	918 (9.8)	619 (5.9)	748 (7.5)	851 (8.9)	2817 (10.4)	1908 (6.1)	2431 (7.7)	2863 (9.3)
Smoking	Never	5744 (49.5)	6037 (50.2)	6028 (53.1)	5722 (52.6)	11344 (33.5)	12089 (33.6)	12872 (36.2)	12941 (36.6)
	Ex-smoker	4368 (37.7)	4477 (37.2)	3944 (34.8)	3933 (36.1)	17927 (53.0)	19089 (53.0)	18131 (51.0)	18051 (51.1)
	Current	1487 (12.8)	1517 (12.6)	1376 (12.1)	1231 (11.3)	4547 (13.4)	4828 (13.4)	4538 (12.8)	4360 (12.3)
Dialysis		101 (0.7)	82 (0.6)	85 (0.7)	119 (1.0)	299 (0.7)	294 (0.7)	289 (0.7)	403 (1.0)
Diabetes		2736 (20.8)	2839 (21.7)	2809 (23.3)	2739 (23.5)	7466 (19.5)	8258 (21.1)	8361 (22.1)	8847 (23.5)
Previous PCI		2933 (22.7)	3600 (27.6)	3760 (30.6)	4043 (34.1)	10412 (27.8)	13404 (34.2)	14590 (37.7)	15981 (41.5)
Previous CABG		1336 (15.7)	850 (8.7)	810 (8.4)	766 (8.2)	5169 (20.5)	4116 (14.1)	4216 (13.6)	4161 (13.6)
Previous MI		3174 (27.1)	3640 (30.0)	3324 (28.4)	3267 (28.5)	12216 (35.9)	13370 (36.9)	13270 (36.2)	13861 (37.3)
Previous CVA		463 (3.5)	558 (4.3)	531 (4.4)	461 (4.0)	1149 (3.0)	1445 (3.7)	1403 (3.7)	1377 (3.7)
High cholesterol		8050 (61.6)	8780 (68.0)	7889 (65.4)	7464 (64.9)	22880 (60.3)	26255 (67.5)	24512 (64.8)	24058 (64.6)
Hypertension		7759 (59.4)	8633 (66.8)	8190 (67.9)	7803 (67.8)	19934 (52.6)	23528 (60.5)	23209 (61.4)	23498 (63.1)
Peripheral Vascular Disease		589 (4.5)	670 (5.2)	677 (5.6)	583 (5.1)	1739 (4.6)	2208 (5.7)	2103 (5.6)	1947 (5.2)
Valvular Heart Disease		163 (1.2)	236 (1.8)	324 (2.7)	330 (2.9)	318 (0.8)	534 (1.4)	660 (1.7)	776 (2.1)
LVEF	Good (>50%)	6106 (84.2)	6684 (83.3)	6158 (84.9)	6043 (84.3)	16388 (78.8)	18962 (78.2)	18424 (79.7)	18687 (79.4)
	Fair (30-50%)	871 (12.0)	1109 (13.8)	938 (12.9)	942 (13.1)	3338 (16.1)	4268 (17.6)	3851 (16.7)	3985 (16.9)
	Poor (<30%)	277 (3.8)	228 (2.8)	161 (2.2)	182 (2.5)	1069 (5.1)	1007 (4.2)	838 (3.6)	866 (3.7)
Chronic total occlusions		1396 (10.9)	1348 (11.2)	1203 (10.5)	1168 (10.5)	5159 (13.8)	5438 (14.8)	5239 (14.4)	5266 (14.4)
Access	Femoral only	9531 (74.2)	8080 (61.7)	6070 (48.9)	4348 (36.5)	26587 (71.0)	22678 (57.4)	16738 (42.8)	11676 (30.2)
	Radial only	3146 (24.5)	4724 (36.1)	5959 (48.0)	7073 (59.3)	10475 (28.0)	15995 (40.5)	21129 (54.0)	25454 (65.7)
	Multiple	118 (0.9)	252 (1.9)	321 (2.6)	456 (3.8)	298 (0.8)	650 (1.6)	1093 (2.8)	1456 (3.8)
	Other	48 (0.4)	48 (0.4)	55 (0.4)	46 (0.4)	80 (0.2)	154 (0.4)	142 (0.4)	137 (0.4)
LMS vessel PCI		345 (2.5)	439 (3.3)	462 (3.7)	597 (4.9)	1186 (2.9)	1371 (3.4)	1588 (4.0)	2164 (5.5)
Multivessel PCI		2080 (15.0)	2053 (15.3)	1832 (14.5)	1763 (14.6)	6966 (17.3)	6821 (16.9)	6751 (17.0)	6360 (16.2)

For peer review only

Table A2b Baseline patient characteristics for UA/NSTEMI procedures

		Female				Male			
		**2007-2008**	**2009-2010**	**2011-2012**	**2013-2014**	**2007-2008**	**2009-2010**	**2011-2012**	**2013-2014**
**Number of procedures**		12756	13858	15139	15594	33797	37049	39379	40749
**Mean age, years (SD)**		67.3 (11.5)	68.4 (11.9)	68.9 (12.2)	69.1 (12.2)	62.3 (11.8)	63.2 (11.9)	64.0 (12.2)	64.5 (12.3)
**Variable, N (%)**									
Age	<50	1072 (8.4)	1054 (7.6)	1193 (7.9)	1163 (7.5)	5161 (15.3)	5181 (14.0)	5069 (12.9)	4941 (12.1)
	50s	2009 (15.7)	2149 (15.5)	2199 (14.5)	2282 (14.6)	8787 (26.0)	9093 (24.5)	9418 (23.9)	9644 (23.7)
	60s	3665 (28.7)	3546 (25.6)	3767 (24.9)	3931 (25.2)	9925 (29.4)	10792 (29.1)	11207 (28.5)	11524 (28.3)
	70s	4228 (33.1)	4546 (32.8)	4764 (31.5)	4825 (30.9)	7452 (22.0)	8602 (23.2)	9195 (23.4)	9555 (23.4)
	80+	1782 (14.0)	2563 (18.5)	3216 (21.2)	3393 (21.8)	2472 (7.3)	3381 (9.1)	4490 (11.4)	5085 (12.5)
Ethnicity	Asian	510 (5.7)	565 (5.1)	617 (5.1)	792 (6.5)	1513 (6.4)	1848 (6.3)	2055 (6.6)	2317 (7.3)
	Black	65 (0.7)	102 (0.9)	136 (1.1)	139 (1.1)	163 (0.7)	240 (0.8)	242 (0.8)	280 (0.9)
	White	7764 (86.1)	9829 (88.2)	10607 (87.4)	10345 (84.9)	20211 (85.2)	25772 (87.3)	26894 (85.8)	26859 (84.3)
	Other	676 (7.5)	645 (5.8)	778 (6.4)	910 (7.5)	1847 (7.8)	1647 (5.6)	2158 (6.9)	2388 (7.5)
Smoking	Never	4403 (41.0)	5405 (43.9)	6332 (46.1)	6709 (47.0)	7547 (26.2)	9068 (27.2)	10987 (30.6)	11925 (31.7)
	Ex-smoker	3563 (33.2)	3855 (31.3)	4240 (30.9)	4459 (31.2)	12572 (43.7)	14408 (43.2)	15068 (41.9)	16149 (42.9)
	Current	2773 (25.8)	3058 (24.8)	3161 (23.0)	3116 (21.8)	8653 (30.1)	9860 (29.6)	9865 (27.5)	9603 (25.5)
Dialysis		140 (1.1)	139 (1.0)	165 (1.1)	215 (1.4)	365 (1.1)	364 (1.0)	452 (1.1)	523 (1.3)
Diabetes		2358 (19.5)	2858 (21.3)	3438 (23.6)	3777 (25.1)	5305 (16.6)	6561 (18.2)	7868 (20.8)	9027 (22.9)
Previous PCI		1741 (14.7)	2180 (16.2)	2555 (17.3)	3008 (19.7)	5000 (15.9)	6356 (17.7)	8101 (21.1)	8959 (22.4)
Previous CABG		891 (11.3)	748 (7.5)	778 (6.7)	816 (6.9)	3161 (14.8)	2781 (10.4)	3450 (11.3)	3461 (11.1)
Previous MI		3080 (28.9)	3298 (26.8)	3639 (25.8)	3833 (26.1)	8909 (31.6)	9730 (29.4)	11017 (30.1)	11336 (29.6)
Previous CVA		507 (4.2)	750 (5.6)	827 (5.7)	822 (5.5)	1074 (3.3)	1616 (4.5)	1777 (4.7)	1916 (4.9)
High cholesterol		7164 (58.9)	8344 (61.9)	8586 (58.9)	8454 (56.7)	18157 (56.5)	21576 (60.0)	21564 (56.9)	21501 (55.1)
Hypertension		6982 (57.4)	8447 (62.7)	9129 (62.7)	9540 (64.0)	15573 (48.5)	19115 (53.2)	20669 (54.5)	21892 (56.1)
Peripheral Vascular Disease		599 (4.9)	775 (5.8)	858 (5.9)	895 (6.0)	1486 (4.6)	2027 (5.6)	2232 (5.9)	2472 (6.3)
Valvular Heart Disease		118 (1.0)	207 (1.5)	301 (2.1)	335 (2.2)	223 (0.7)	328 (0.9)	542 (1.4)	684 (1.8)
LVEF	Good (>50%)	4523 (69.1)	5000 (67.4)	5340 (69.1)	5569 (68.0)	11530 (66.7)	12756 (64.8)	13090 (67.0)	13985 (65.9)
	Fair (30-50%)	1638 (25.0)	1982 (26.7)	1907 (24.7)	2102 (25.7)	4557 (26.4)	5540 (28.2)	5047 (25.8)	5570 (26.3)
	Poor (<30%)	382 (5.8)	440 (5.9)	478 (6.2)	520 (6.3)	1190 (6.9)	1378 (7.0)	1408 (7.2)	1652 (7.8)
Chronic total occlusions		647 (5.5)	578 (4.6)	623 (4.5)	572 (3.9)	1886 (6.1)	1836 (5.4)	1867 (5.2)	2007 (5.3)
Shock		135 (1.1)	175 (1.3)	205 (1.4)	217 (1.4)	319 (1.0)	417 (1.1)	475 (1.2)	605 (1.5)
Ventilated		95 (0.9)	104 (0.9)	168 (1.2)	163 (1.1)	218 (0.8)	327 (1.0)	449 (1.2)	530 (1.4)
IABP		177 (1.6)	192 (1.5)	211 (1.4)	160 (1.1)	398 (1.3)	443 (1.3)	471 (1.2)	395 (1.0)
Cardio-pulmonary support		4 (0.0)	9 (0.1)	10 (0.1)	7 (0.0)	11 (0.0)	21 (0.1)	17 (0.0)	19 (0.0)
Inotropes		47 (0.4)	71 (0.6)	77 (0.5)	77 (0.5)	113 (0.4)	149 (0.4)	163 (0.4)	231 (0.6)
Access	Femoral only	8724 (72.1)	7538 (55.4)	6202 (41.5)	4681 (30.4)	21963 (68.5)	18419 (50.6)	13905 (35.8)	10146 (25.2)
	Radial only	3209 (26.5)	5740 (42.2)	8236 (55.1)	10106 (65.7)	9759 (30.4)	17346 (47.7)	24030 (61.9)	28948 (72.0)
	Multiple	134 (1.1)	302 (2.2)	456 (3.1)	546 (3.6)	294 (0.9)	563 (1.5)	801 (2.1)	1019 (2.5)
	Other	39 (0.3)	34 (0.2)	48 (0.3)	46 (0.3)	57 (0.2)	74 (0.2)	90 (0.2)	86 (0.2)
LMS vessel PCI		434 (3.4)	594 (4.3)	749 (4.9)	807 (5.2)	971 (2.9)	1192 (3.2)	1607 (4.1)	2187 (5.4)
Multivessel PCI		2110 (16.5)	2159 (15.6)	2307 (15.2)	2394 (15.4)	5825 (17.2)	5951 (16.1)	6248 (15.9)	6772 (16.6)

For peer review only

Table A2c Baseline patient characteristics for STEMI procedures

		Female				Male			
		**2007-2008**	**2009-2010**	**2011-2012**	**2013-2014**	**2007-2008**	**2009-2010**	**2011-2012**	**2013-2014**
**Number of procedures**		4043	7608	10480	10908	12921	22306	30244	31066
**Mean age, years (SD)**		67.1 (12.7)	68.4 (12.9)	68.7 (13.2)	68.6 (13.3)	60.5 (12.3)	61.2 (12.4)	61.6 (12.6)	61.8 (12.4)
**Variable, N (%)**									
Age	<50	422 (10.4)	699 (9.2)	958 (9.1)	1035 (9.5)	2586 (20.0)	4178 (18.7)	5429 (18.0)	5350 (17.2)
	50s	678 (16.8)	1207 (15.9)	1653 (15.8)	1754 (16.1)	3545 (27.4)	6091 (27.3)	8176 (27.0)	8532 (27.5)
	60s	1042 (25.8)	1866 (24.5)	2539 (24.2)	2629 (24.1)	3564 (27.6)	6079 (27.3)	8360 (27.6)	8736 (28.1)
	70s	1202 (29.7)	2179 (28.6)	2898 (27.7)	2879 (26.4)	2364 (18.3)	4157 (18.6)	5509 (18.2)	5610 (18.1)
	80+	699 (17.3)	1657 (21.8)	2432 (23.2)	2611 (23.9)	862 (6.7)	1801 (8.1)	2770 (9.2)	2838 (9.1)
Ethnicity	Asian	164 (5.4)	269 (4.3)	397 (4.4)	389 (4.3)	802 (8.3)	1296 (7.2)	1845 (7.1)	1801 (7.0)
	Black	27 (0.9)	41 (0.7)	67 (0.7)	74 (0.8)	102 (1.1)	144 (0.8)	193 (0.7)	226 (0.9)
	White	2674 (88.0)	5705 (91.6)	8150 (90.8)	8221 (90.8)	8110 (84.4)	15982 (88.2)	22654 (87.5)	22384 (87.1)
	Other	173 (5.7)	212 (3.4)	363 (4.0)	368 (4.1)	591 (6.2)	696 (3.8)	1185 (4.6)	1279 (5.0)
Smoking	Never	1359 (39.7)	2603 (38.6)	3991 (43.1)	4167 (43.2)	2910 (26.1)	5470 (27.4)	8538 (31.4)	8956 (32.0)
	Ex-smoker	790 (23.1)	1542 (22.9)	1904 (20.6)	2155 (22.3)	3530 (31.7)	5900 (29.6)	7607 (28.0)	8085 (28.8)
	Current	1278 (37.3)	2593 (38.5)	3357 (36.3)	3325 (34.5)	4690 (42.1)	8575 (43.0)	11042 (40.6)	10989 (39.2)
Dialysis		32 (0.8)	40 (0.5)	38 (0.4)	46 (0.4)	79 (0.6)	85 (0.4)	125 (0.4)	127 (0.4)
Diabetes		639 (16.7)	1091 (14.8)	1557 (15.6)	1744 (16.9)	1609 (13.1)	2681 (12.4)	3877 (13.5)	4320 (14.6)
Previous PCI		314 (8.3)	551 (7.4)	751 (7.4)	798 (7.5)	1287 (10.7)	2233 (10.3)	2938 (10.0)	3401 (11.2)
Previous CABG		195 (8.7)	154 (2.9)	256 (3.3)	269 (3.4)	707 (9.8)	727 (4.7)	959 (4.2)	1033 (4.6)
Previous MI		584 (16.3)	840 (12.1)	1037 (10.7)	1074 (10.6)	2150 (18.8)	2976 (14.7)	3761 (13.4)	4021 (13.9)
Previous CVA		157 (4.1)	362 (5.0)	461 (4.6)	487 (4.7)	375 (3.1)	699 (3.3)	984 (3.4)	975 (3.3)
High cholesterol		1695 (44.5)	3142 (43.0)	3997 (40.0)	3987 (38.9)	5091 (41.8)	9063 (42.3)	11286 (39.2)	11200 (38.3)
Hypertension		1838 (48.3)	3503 (47.9)	4776 (47.8)	5013 (48.9)	4649 (38.2)	8427 (39.4)	11215 (39.0)	11724 (40.1)
Peripheral Vascular Disease		140 (3.7)	253 (3.5)	374 (3.7)	395 (3.9)	420 (3.4)	657 (3.1)	953 (3.3)	952 (3.3)
Valvular Heart Disease		27 (0.7)	47 (0.6)	131 (1.3)	107 (1.0)	44 (0.4)	80 (0.4)	198 (0.7)	172 (0.6)
LVEF	Good (>50%)	648 (47.3)	1136 (47.9)	1669 (53.1)	1572 (47.9)	2183 (49.7)	3452 (48.0)	4926 (53.9)	4749 (49.1)
	Fair (30-50%)	556 (40.6)	957 (40.4)	1183 (37.7)	1338 (40.7)	1560 (35.5)	2883 (40.0)	3380 (37.0)	3842 (39.8)
	Poor (<30%)	166 (12.1)	277 (11.7)	290 (9.2)	375 (11.4)	646 (14.7)	864 (12.0)	830 (9.1)	1073 (11.1)
Chronic total occlusions		172 (4.6)	269 (3.9)	218 (2.3)	239 (2.4)	662 (5.5)	855 (4.2)	705 (2.6)	769 (2.7)
Shock		325 (8.2)	526 (7.0)	851 (8.2)	1055 (9.8)	811 (6.4)	1364 (6.2)	1973 (6.6)	2482 (8.1)
Ventilated		113 (3.3)	198 (3.0)	371 (4.0)	458 (4.7)	318 (2.9)	622 (3.2)	1201 (4.5)	1477 (5.3)
IABP		272 (7.3)	391 (5.7)	478 (4.8)	381 (3.6)	718 (6.0)	1117 (5.5)	1334 (4.7)	1090 (3.6)
Cardio-pulmonary support		10 (0.3)	23 (0.3)	44 (0.4)	28 (0.3)	30 (0.3)	57 (0.3)	99 (0.3)	82 (0.3)
Inotropes		123 (3.3)	178 (2.6)	268 (2.7)	323 (3.1)	272 (2.3)	428 (2.1)	579 (2.0)	798 (2.7)
Access	Femoral only	2942 (75.9)	4441 (59.5)	4573 (44.3)	3406 (31.7)	8928 (71.9)	11990 (54.6)	11216 (37.7)	7580 (24.8)
	Radial only	868 (22.4)	2776 (37.2)	5385 (52.1)	6881 (63.9)	3314 (26.7)	9483 (43.2)	17852 (60.0)	22168 (72.4)
	Multiple	60 (1.5)	237 (3.2)	367 (3.6)	449 (4.2)	147 (1.2)	439 (2.0)	658 (2.2)	806 (2.6)
	Other	6 (0.2)	11 (0.1)	9 (0.1)	24 (0.2)	22 (0.2)	32 (0.1)	45 (0.2)	44 (0.1)
LMS vessel PCI		81 (2.0)	145 (1.9)	219 (2.1)	330 (3.0)	249 (1.9)	423 (1.9)	638 (2.1)	1097 (3.5)
Multivessel PCI		327 (8.1)	458 (6.0)	576 (5.5)	619 (5.7)	1066 (8.3)	1432 (6.4)	1831 (6.1)	2031 (6.5)

For peer review only

Table A3 Crude and relative survival estimates with 95% confidence limits

Year	30-days				365-days			
	Crude survival	Crude survival 95% CI	Relative survival	Relative survival 95% CI	Crude survival	Crude survival 95% CI	Relative survival	Relative survival 95% CI
**Female, Elective**								
**2007-2008**	0.996	(0.994, 0.997)	0.997	(0.996, 0.998)	0.977	(0.974, 0.979)	0.995	(0.992, 0.997)
**2009-2010**	0.996	(0.995, 0.997)	0.998	(0.997, 0.999)	0.979	(0.977, 0.981)	0.998	(0.996, 1.001)
**2011-2012**	0.995	(0.994, 0.996)	0.997	(0.995, 0.998)	0.973	(0.970, 0.976)	0.993	(0.990, 0.996)
**2013-2014**	0.995	(0.994, 0.997)	0.997	(0.996, 0.998)	0.975	(0.972, 0.978)	0.995	(0.992, 0.998)
**Female, UA/NSTEMI**								
**2007-2008**	0.983	(0.980, 0.985)	0.985	(0.982, 0.987)	0.946	(0.942, 0.950)	0.967	(0.963, 0.971)
**2009-2010**	0.980	(0.978, 0.983)	0.982	(0.980, 0.985)	0.939	(0.935, 0.943)	0.962	(0.957, 0.966)
**2011-2012**	0.977	(0.974, 0.979)	0.979	(0.977, 0.981)	0.937	(0.933, 0.941)	0.960	(0.956, 0.964)
**2013-2014**	0.978	(0.976, 0.980)	0.980	(0.978, 0.982)	0.937	(0.933, 0.941)	0.961	(0.957, 0.965)
**Female, STEMI**								
**2007-2008**	0.920	(0.912, 0.928)	0.922	(0.913, 0.930)	0.875	(0.864, 0.884)	0.895	(0.884, 0.905)
**2009-2010**	0.919	(0.913, 0.925)	0.921	(0.915, 0.927)	0.869	(0.861, 0.876)	0.891	(0.883, 0.899)
**2011-2012**	0.914	(0.908, 0.919)	0.916	(0.910, 0.921)	0.865	(0.858, 0.871)	0.888	(0.881, 0.895)
**2013-2014**	0.911	(0.905, 0.916)	0.913	(0.907, 0.918)	0.864	(0.857, 0.870)	0.886	(0.879, 0.893)
**Male, Elective**								
**2007-2008**	0.997	(0.997, 0.998)	0.999	(0.998, 0.999)	0.980	(0.978, 0.981)	1.000	(0.998, 1.001)
**2009-2010**	0.997	(0.996, 0.998)	0.999	(0.998, 0.999)	0.979	(0.977, 0.980)	0.999	(0.998, 1.001)
**2011-2012**	0.997	(0.996, 0.997)	0.999	(0.998, 0.999)	0.978	(0.977, 0.980)	0.999	(0.997, 1.000)
**2013-2014**	0.997	(0.996, 0.997)	0.998	(0.998, 0.999)	0.977	(0.975, 0.978)	0.998	(0.996, 0.999)
**Male, UA/NSTEMI**								
**2007-2008**	0.985	(0.984, 0.987)	0.987	(0.986, 0.988)	0.958	(0.956, 0.960)	0.978	(0.976, 0.980)
**2009-2010**	0.985	(0.984, 0.986)	0.987	(0.986, 0.988)	0.955	(0.953, 0.957)	0.976	(0.974, 0.978)
**2011-2012**	0.984	(0.982, 0.985)	0.986	(0.984, 0.987)	0.949	(0.947, 0.952)	0.971	(0.969, 0.973)
**2013-2014**	0.982	(0.981, 0.983)	0.984	(0.982, 0.985)	0.944	(0.941, 0.946)	0.966	(0.963, 0.968)
**Male, STEMI**								
**2007-2008**	0.947	(0.943, 0.951)	0.949	(0.945, 0.953)	0.920	(0.915, 0.924)	0.936	(0.931, 0.941)
**2009-2010**	0.951	(0.948, 0.954)	0.953	(0.950, 0.955)	0.922	(0.918, 0.925)	0.939	(0.935, 0.942)
**2011-2012**	0.947	(0.944, 0.949)	0.948	(0.945, 0.951)	0.916	(0.913, 0.919)	0.933	(0.930, 0.936)
**2013-2014**	0.942	(0.939, 0.944)	0.943	(0.941, 0.946)	0.913	(0.910, 0.916)	0.930	(0.926, 0.933)

For peer review only

Figure A1 Observed survival rates by patient age, indication, and year of procedure, with 95% confidence limits. P-values from the log-rank test.

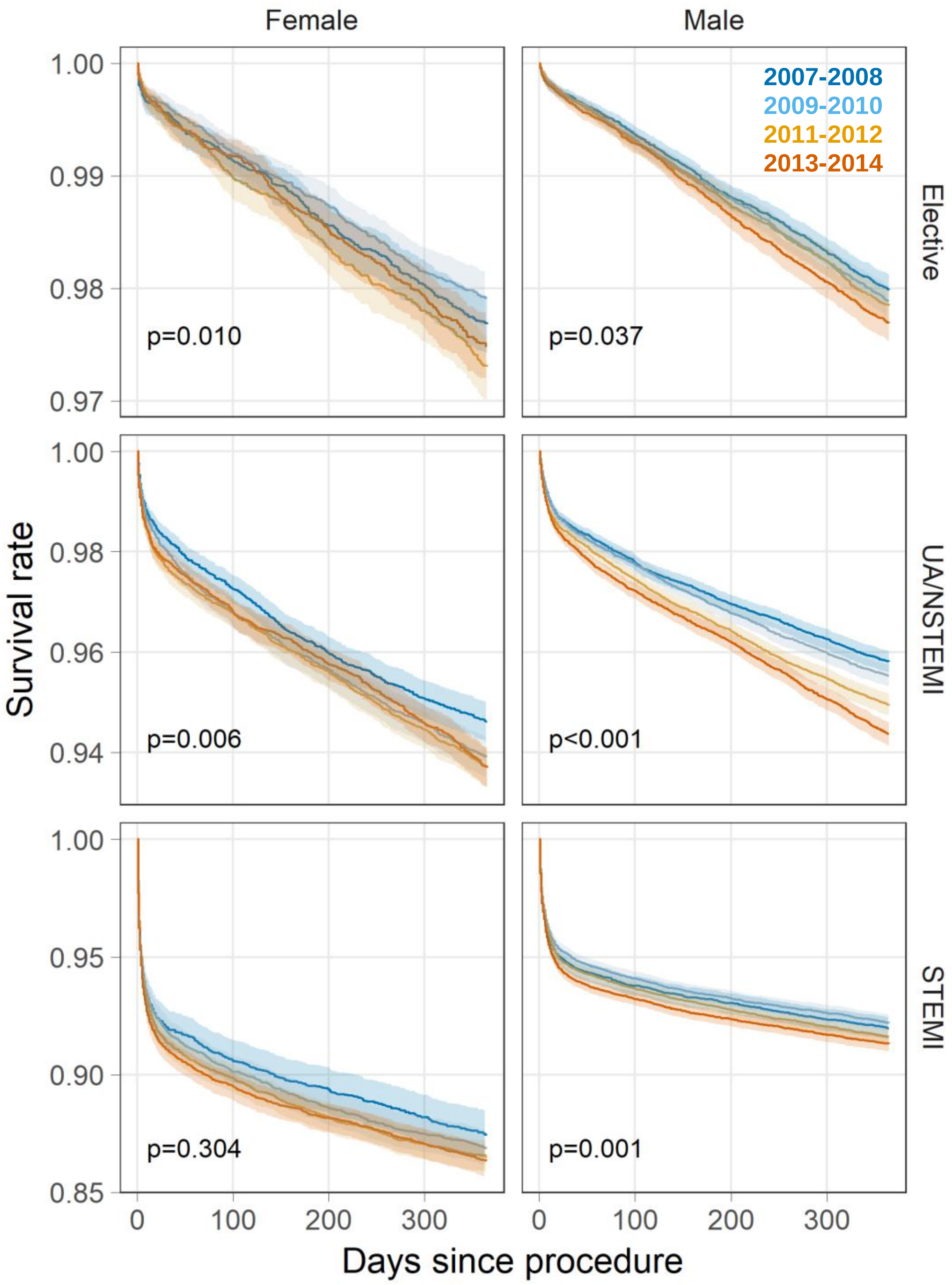

Figure A2 Relative survival estimates by patient age, indication, and year of procedure, with 95% confidence limits. P-values from a log-rank-type test for relative survival curves.

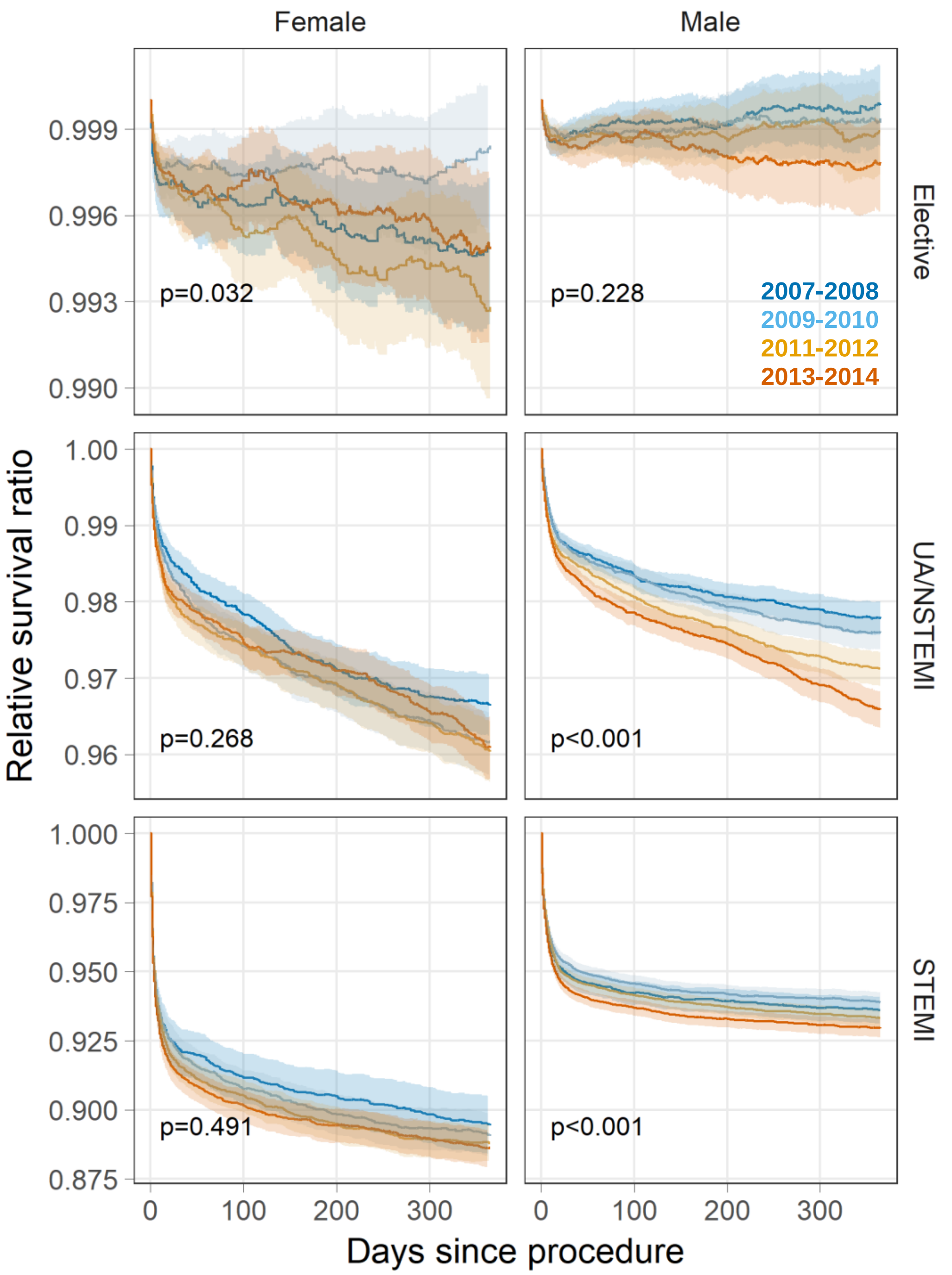

Figure A3 Observed-to-expected one-year mortality ratio (log-scale) by patient age, with 95% confidence limits.

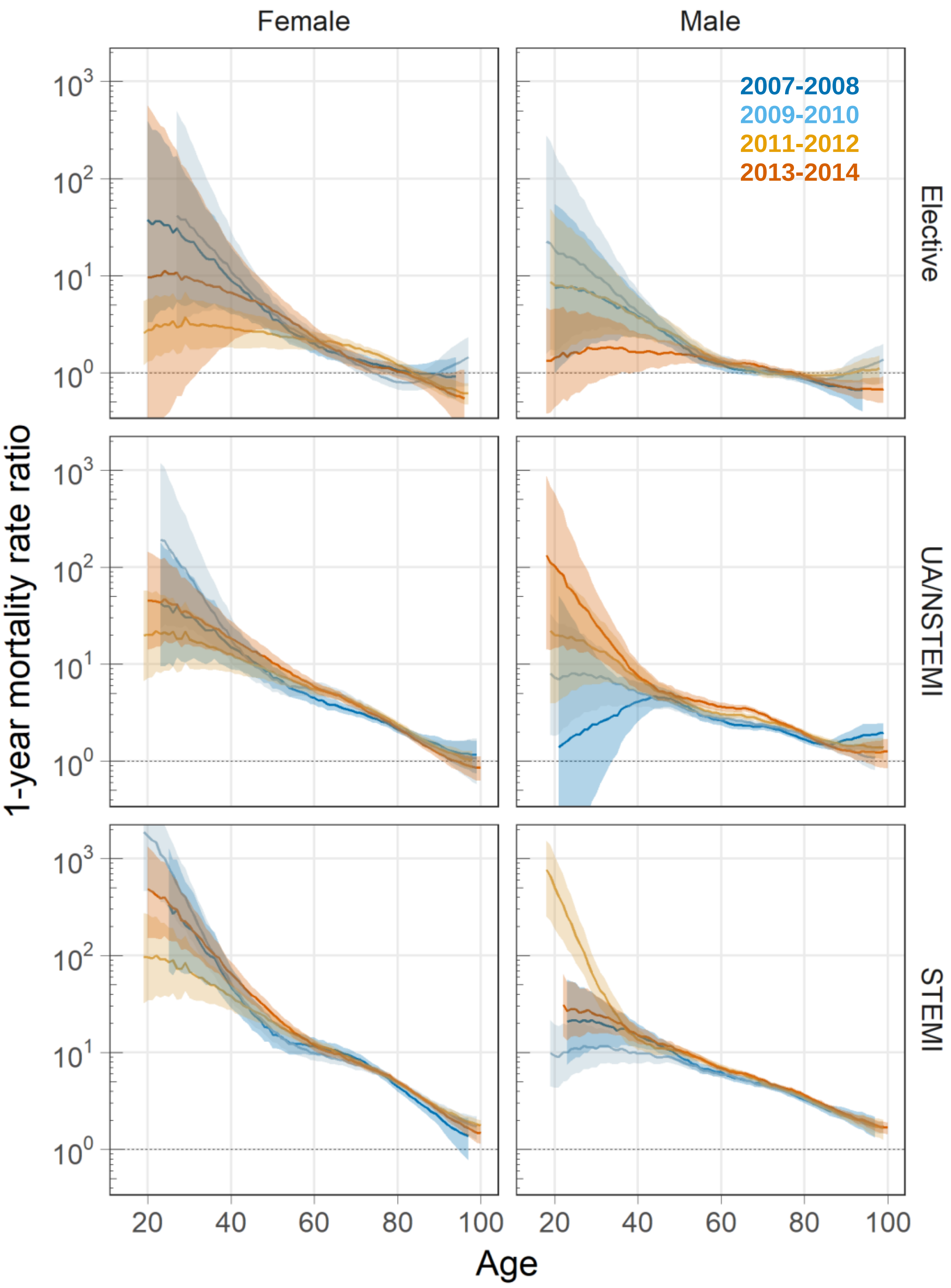

Binomial General Additive Models with 7-knot splines are used for smoothing the observed one-year mortality, then divided by the expected mortality rate. 95% confidence limits are removed for clarity.

Figure A4 Cumulative excess hazard rates by patient age, indication, and year of procedure, with 95% confidence limits.

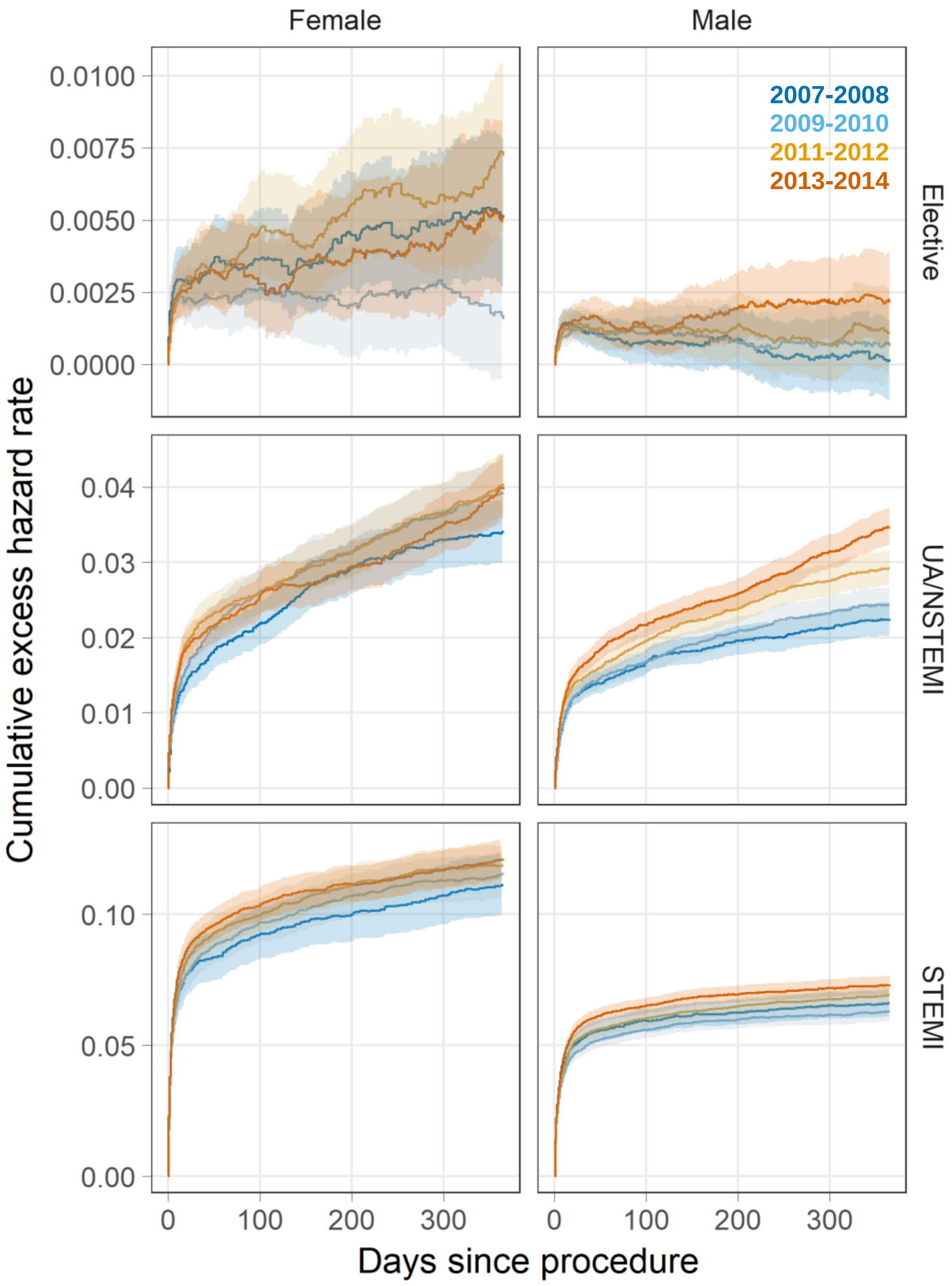

The RECORD statement – checklist of items, extended from the STROBE statement, that should be reported in observational studies using routinely collected health data.

	Item No.	STROBE items	Location in manuscript where items are reported	RECORD items	Location in manuscript where items are reported
**Title and abstract**					
	1	(a) Indicate the study's design with a commonly used term in the title or the abstract (b) Provide in the abstract an informative and balanced summary of what was done and what was found	Title page	RECORD 1.1: The type of data used should be specified in the title or abstract. When possible, the name of the databases used should be included.<br><br>RECORD 1.2: If applicable, the geographic region and timeframe within which the study took place should be reported in the title or abstract.<br><br>RECORD 1.3: If linkage between databases was conducted for the study, this should be clearly stated in the title or abstract.	1.1 Abstract: data<br><br>1.2 Abstract: setting<br><br>1.3 No individual patient record linkage within this study
**Introduction**					
Background rationale	2	Explain the scientific background and rationale for the investigation being reported	Page 3 Intro: para 1-3		
Objectives	3	State specific objectives, including any prespecified hypotheses	Page 3 Intro: para 4		
**Methods**					
Study Design	4	Present key elements of study design early in the paper	Page 4-5. Methods: statistical analysis		
Setting	5	Describe the setting, locations, and relevant dates, including periods of recruitment, exposure, follow-up, and data collection	Page 4-5. Methods		
Participants	6	*(a) Cohort study* - Give the eligibility criteria, and the	(a) Page 4. Methods: PCI procedure data	RECORD 6.1: The methods of study population selection (such as codes or	6.1 Page 4. Methods: PCI

For peer review only

		sources and methods of selection of participants. Describe methods of follow-up<br>*Case-control study* - Give the eligibility criteria, and the sources and methods of case ascertainment and control selection. Give the rationale for the choice of cases and controls<br>*Cross-sectional study* - Give the eligibility criteria, and the sources and methods of selection of participants<br><br>*(b) Cohort study* - For matched studies, give matching criteria and number of exposed and unexposed<br>*Case-control study* - For matched studies, give matching criteria and the number of controls per case	(b) Page 4. Methods: ONS life-table data	algorithms used to identify subjects) should be listed in detail. If this is not possible, an explanation should be provided.<br><br>RECORD 6.2: Any validation studies of the codes or algorithms used to select the population should be referenced. If validation was conducted for this study and not published elsewhere, detailed methods and results should be provided.<br><br>RECORD 6.3: If the study involved linkage of databases, consider use of a flow diagram or other graphical display to demonstrate the data linkage process, including the number of individuals with linked data at each stage.	procedure data<br><br>6.2 N/A No validation – exclusions only due to missing data<br><br>6.3 N/A No individual patient record linkage
Variables	7	Clearly define all outcomes, exposures, predictors, potential confounders, and effect modifiers. Give diagnostic criteria, if applicable.	Page 4. Methods. Mortality only outcome of interest	RECORD 7.1: A complete list of codes and algorithms used to classify exposures, outcomes, confounders, and effect modifiers should be provided. If these cannot be reported, an explanation should be provided.	7.1 N/A
Data sources/ measurement	8	For each variable of interest, give sources of data and details of methods of assessment (measurement).<br>Describe comparability of assessment methods if there is more than one group	Page 4. Methods. Only variables are age, sex, indication, procedure year.		
Bias	9	Describe any efforts to address potential sources of bias			
Study size	10	Explain how the study size was	N/A all available		

		arrived at	procedures included		
Quantitative variables	11	Explain how quantitative variables were handled in the analyses. If applicable, describe which groupings were chosen, and why	Page 4-5. Methods: statistical analysis		
Statistical methods	12	(a) Describe all statistical methods, including those used to control for confounding<br>(b) Describe any methods used to examine subgroups and interactions<br>(c) Explain how missing data were addressed<br>(d) *Cohort study* - If applicable, explain how loss to follow-up was addressed<br>*Case-control study* - If applicable, explain how matching of cases and controls was addressed<br>*Cross-sectional study* - If applicable, describe analytical methods taking account of sampling strategy<br>(e) Describe any sensitivity analyses	Page 4-5. Methods: statistical analysis		
Data access and cleaning methods		..	Page 5. Methods final para.	RECORD 12.1: Authors should describe the extent to which the investigators had access to the database population used to create the study population.<br><br>RECORD 12.2: Authors should provide information on the data cleaning methods used in the study.	Page 5. Methods: statistical analysis final para (+ GitHub page)
Linkage		..		RECORD 12.3: State whether the study included person-level, institutional-	N/A

For peer review only - http://bmjopen.bmj.com/site/about/guidelines.xhtml

				level, or other data linkage across two or more databases. The methods of linkage and methods of linkage quality evaluation should be provided.	
**Results**					
Participants	13	(a) Report the numbers of individuals at each stage of the study (*e.g.*, numbers potentially eligible, examined for eligibility, confirmed eligible, included in the study, completing follow-up, and analysed)<br>(b) Give reasons for non-participation at each stage.<br>(c) Consider use of a flow diagram	Page 7. Table 1<br><br>Supplementary Table A1	RECORD 13.1: Describe in detail the selection of the persons included in the study (*i.e.,* study population selection) including filtering based on data quality, data availability and linkage. The selection of included persons can be described in the text and/or by means of the study flow diagram.	Page 4. Methods: PCI procedure data<br><br>Supplementary Table A1
Descriptive data	14	(a) Give characteristics of study participants (*e.g.*, demographic, clinical, social) and information on exposures and potential confounders<br>(b) Indicate the number of participants with missing data for each variable of interest<br>(c) *Cohort study* - summarise follow-up time (*e.g.*, average and total amount)	(a, b) Page 8-10 Tables 2a, 2b, 2c<br><br>(c) Page 5. Results para 1		
Outcome data	15	*Cohort study* - Report numbers of outcome events or summary measures over time<br>*Case-control study* - Report numbers in each exposure category, or summary measures of exposure<br>*Cross-sectional study* - Report numbers of outcome events or summary measures	Figure 1<br><br>Supplementary Table A2		
Main results	16	(a) Give unadjusted estimates	(a) Figure 1		

		and, if applicable, confounder-adjusted estimates and their precision (e.g., 95% confidence interval). Make clear which confounders were adjusted for and why they were included<br>(b) Report category boundaries when continuous variables were categorized<br>(c) If relevant, consider translating estimates of relative risk into absolute risk for a meaningful time period	Tables 2a, 2b, 2c<br>Table A2<br><br>(b) N/A<br>(c) N/A		
Other analyses	17	Report other analyses done—e.g., analyses of subgroups and interactions, and sensitivity analyses	N/A		
**Discussion**					
Key results	18	Summarise key results with reference to study objectives	Page 11. Discussion para 2, 4		
Limitations	19	Discuss limitations of the study, taking into account sources of potential bias or imprecision. Discuss both direction and magnitude of any potential bias	Page 13. Discussion: limitations	RECORD 19.1: Discuss the implications of using data that were not created or collected to answer the specific research question(s). Include discussion of misclassification bias, unmeasured confounding, missing data, and changing eligibility over time, as they pertain to the study being reported.	
Interpretation	20	Give a cautious overall interpretation of results considering objectives, limitations, multiplicity of analyses, results from similar studies, and other relevant evidence	Page 11-12. Discussion para 2-8		
Generalisability	21	Discuss the generalisability (external validity) of the study results	Page 12. Discussion para 9		

For peer review only

Other Information					
Funding	22	Give the source of funding and the role of the funders for the present study and, if applicable, for the original study on which the present article is based	Provided in journal submission		
Accessibility of protocol, raw data, and programming code		..		RECORD 22.1: Authors should provide information on how to access any supplemental information such as the study protocol, raw data, or programming code.	GitHub page provided

*Reference: Benchimol EI, Smeeth L, Guttmann A, Harron K, Moher D, Petersen I, Sørensen HT, von Elm E, Langan SM, the RECORD Working Committee. The REporting of studies Conducted using Observational Routinely-collected health Data (RECORD) Statement. *PLoS Medicine* 2015; in press.

*Checklist is protected under Creative Commons Attribution (CC BY) license.

For peer review only

Temporal trends in Relative Survival Following Percutaneous Coronary Intervention

Journal:	*BMJ Open*
Manuscript ID	bmjopen-2018-024627.R1
Article Type:	Research
Date Submitted by the Author:	16-Nov-2018

Complete List of Authors:	Hulme, William; University of Manchester, Institute of Population Health<br>Sperrin, Matthew; University of Manchester Institute of Population Health,<br>Martin, Glen; University of Manchester Institute of Population Health, Farr Institute<br>Curzen, Nick; University Hospital Southampton NHS F Trust, Wessex Cardiac Unit<br>Ludman, Peter; Queen Elizabeth Hospital<br>Kontopantelis, Evangelos; University of Manchester, Faculty of Biology, Medicine and Health<br>Mamas, Mamas; University of Keele and Academic Department of Cardiology, Institute of Primary Care and Health Sciences

**Primary Subject Heading**:	Cardiovascular medicine
Secondary Subject Heading:	Health services research
Keywords:	Coronary intervention < CARDIOLOGY, Quality in health care < HEALTH SERVICES ADMINISTRATION & MANAGEMENT, STATISTICS & RESEARCH METHODS

SCHOLARONE™ Manuscripts

Temporal trends in Relative Survival Following Percutaneous Coronary Intervention

Brief title: Relative survival after PCI

William J Hulme [1], Matthew Sperrin [1], Glen Martin [1], Nick Curzen [2], Peter Ludman [3], Evangelos Kontopantelis[1], Mamas A Mamas[4]; On behalf of the British Cardiovascular Intervention Society and the National Institute of Cardiovascular Outcomes Research.

1. Farr Institute, Faculty of Biology, Medicine and Health, University of Manchester, Manchester Academic Health Science Centre, Manchester, United Kingdom

2. University Hospital Southampton and Faculty of Medicine, University of Southampton, UK

3. Queen Elizabeth Hospital, Birmingham, UK.

4. Keele Cardiovascular Research Group, Centre for Prognosis Research, Institute of Primary Care and Health Sciences, University of Keele and Academic Department of Cardiology, Royal Stoke Hospital, Stoke-on-Trent, UK

Corresponding author:

William Hulme
Vaughan House
Portmouth Street
M13 9GB
Manchester, United Kingdom
Email: william.hulme@manchester.ac.uk

Word Count: 2927

For peer review only

ABSTRACT

Objective Percutaneous Coronary Intervention (PCI) has seen substantial shifts in patient selection in recent years that have increased baseline patient mortality risk. It is unclear to what extent observed changes in mortality are attributable to background mortality risk or the indication and selection for PCI itself. PCI-attributable mortality can be estimated using Relative Survival, which adjusts observed mortality by that seen in a matched control population. We report relative survival ratios and compare these across different time periods.

Methods National Health Service PCI activity in England and Wales from 2007 to 2014 is considered using data from the British Cardiovascular Intervention Society PCI Registry. Background mortality is as reported in Office for National Statistics life tables. Relative survival ratios up to one-year are estimated, matching on patient age, sex, and procedure date. Estimates are stratified by indication for PCI, sex, and procedure date.

Results 549,305 procedures were studied after exclusions for missing age, sex, indication, and mortality status. Comparing from 2007-08 to 2013-14, differences in crude survival at one-year were consistently lower in later years across all strata. For relative survival, these differences remained but were smaller, suggesting poorer survival in later years is partly due to demographic characteristics. Relative survival was higher in older patients.

Conclusions Changes in patient demographics account for some but not all of the crude survival changes seen during the study period. Relative survival is an under-used methodology in interventional settings like PCI and should be considered wherever survival is compared between populations with different demographic characteristics, such as between countries or time periods.

For peer review only

Strengths and limitations of this study

- Using national registry data, this study is the first to compare patient survival after Percutaneous Coronary Intervention with matched population survival across an entire healthcare system.
- It provides a template for similar investigations in other interventional settings where relative survival is under-used.
- Demographic matching in addition to age, sex, and year of procedure was not possible, though life-tables stratified by other characteristics may be available from the Office for National Statistics in future.

For peer review only

INTRODUCTION

The worldwide use of Percutaneous Coronary Intervention (PCI) for coronary revascularisation has expanded in recent years[1–6] though differences in this change have been observed internationally.[7,8] In the UK, the number of procedures increased from around 78,000 in 2007 to 97,000 in 2016, with the proportion of acute coronary syndrome (ACS) cases increasing from 53.6% to 64.3%[9] in the same period. In the USA, data from the CathPCI registry indicate an increase in the proportion of PCIs for ACS from 57.0% to 64.3% from 2011 to 2014.[3,10]

Correspondingly, patient case-mix has also changed such that PCI is now more likely to be performed in older, more co-morbid patients at higher risk of early mortality. For instance, in the UK between 2007 and 2015, mean patient age increased from 63.6 to 65.1 years and the proportion of patients with diabetes rose from 17.5% to 22.0%.[9] This is due, in part, to more permissive patient selection criteria and a move to more emergent indications where there is less scope for case selection and increased access,[11,12] though changes in the characteristics of the general population may partially explain this transition. Indeed, improvements in secondary prevention for cardiovascular disease and wider societal changes in general may be a significant driver of changes to baseline patient risk given a persistent, albeit slowing, decline in age-standardised cardiovascular mortality in both the UK[13] and the USA.[14] In addition, the evidence-base concerning optimal intervention techniques and treatment strategies has evolved, prompting changes to procedural practice towards the treatment of more complex disease.[15–17]

Mortality rates following PCI will be affected by these trends, though it is unclear to what extent the any changes are related to the baseline mortality risk of selected patients or procedural practice. Consequently, there is a need to report mortality rates from national PCI registries within the context of the changing PCI patient demographic, relative to corresponding changes in the background population.

Relative Survival is a statistical technique that compares the survival of a disease or treatment group with the survival of the general population matched on one or more characteristics, such as age and sex, for example as presented in national life-expectancy tables.[18,19] The excess mortality – that is, the mortality after adjusting for the expected mortality estimated from the control population – associated with that group can then be quantified. Relative Survival methods are typically used to study survival differences following disease diagnosis or to compare long-term treatment strategies where excess mortality develops slowly, for example in cancer.[20,21] More recently it is being considered

For peer review only

in cardiovascular settings,[22–24] including PCI,[25–29] though these have focused on long-term survival and did not consider changes over time.

We aimed to compare mortality rates following PCI with age- and sex-matched mortality rates found nationally, and to examine if these comparisons differ between early or contemporary cohorts.

METHODS

PCI procedure data

The British Cardiovascular Intervention Society (BCIS) audits all PCI activity in the United Kingdom, with data collection managed by the National Institute for Cardiovascular Outcomes Research (NICOR). Information on each procedure is captured locally by hospitals and uploaded to a central server. Mortality tracking is available via the Office for National Statistics (ONS) for procedures in England and Wales for patients with a valid NHS number. Mortality tracking was available up to May 2015.

Procedures in England and Wales between 2007 and 2014 for patients aged between 18 and 100 years old were extracted. Procedures with missing age, sex, indication, and mortality status were excluded.

ONS life-table data

Expected mortality rates for England and Wales are available from ONS national life-tables stratified by age in one-year bands, sex, and calendar year.[30]

Statistical analysis

Each procedure was matched to expected mortality life-table data based on the patients' age, sex, and the date of the procedure. Survival models were stratified by procedure date (2007-08, 2009-10, 2011-12, 2013-14), and by patient sex and indication (elective, UA/NSTEMI, STEMI) as these are known determinants of survival and there are distinct survival differences between these groups. Two-year time periods were chosen to ensure temporal trends are sufficiently captured while maintaining clarity of exposition. Analyses consider mortality up to one year post-procedure with mortality censored thereafter, as long-term estimates are confounded by the increasing influence of competing contributors to mortality and time-varying risk factors.[31]

For peer review only

Crude survival rates are plotted, with survival differences between periods compared using the log-rank test.[32,33] Relative survival models are built that adjust for expected survival using the Ederer II method.[18,19] Relative survival estimates are plotted, and differences between periods compared using the log-rank test after a transformation of survival time described by Stare, Henderson and Pohar.[34] The observed (PCI) and expected (life-table) one-year mortality rates are also compared by considering their ratios across different ages. This expresses the relationship of relative survival and age without explicitly modelling the hazard function, which is challenging in this setting since the hazard falls extremely steeply in the early stages of follow up. This quantity is plotted, using General Additive Models with a binomial link to smooth observed mortality.

The relative survival estimate, calculable at each day post-procedure, is the ratio of the observed survival proportion of those who had PCI to the expected survival proportion based on a matched population. This quantity can interpreted as the post-PCI survival rate if patients were only exposed to PCI-related mortality risk and not other background risks. PCI-related mortality risk should be understood as the mortality risk of being a person who had PCI, not simply the risk of the procedure itself. If the relative survival estimate levels-off after a given time then, on average, there is no additional PCI-related mortality risk for those who survived up to that time. If the relative survival estimate increases then, on average, mortality was less than expected had the patients not needed PCI. In most interventional settings this quantity is typically less than one, indicating that PCI is associated with a greater mortality risk than no PCI.

All analyses were performed using R version 3.4.4.[35] The tidyverse data manipulation and visualization suite[36] was used throughout. The relsurv package[37–39] was used for relative survival modelling. The complete R script is available on GitHub,[40] with synthetic BCIS data provided to replicate analysis steps without disclosing proprietary BCIS data. Access to study materials was only required for the first author.

Patient involvement

This is a registry-based study with all data collected prior to the design phase and outcomes necessarily restricted to mortality only. Therefore, patients were not involved in the design of this study.

For peer review only

RESULTS

In total, there were 575,203 procedures recorded in the BCIS-NICOR registry between 2007 and 2014 in England and Wales, of which 549,305 (95.5%) were available for analysis after exclusions due to missing data (see Supplementary Table A1). Follow-up is complete for every procedure up to one-year (i.e., no censoring occurred before one year), except for procedures from June to December 2014, where available follow-up time was less than one year.

Table 1 presents procedure numbers by year, sex, and indication, and Supplementary Tables A2a, A2b, and A2c present baseline patient characteristics and mortality rates for elective, UA/NSTEMI, and STEMI procedures, respectively. Female patients made up a quarter (25.9%) of procedures. Mean patient age increased from 63.8 years old in 2007-08 to 65.1 years old in 2013-14, with similar increases observed within indications (64.5 to 65.8 years for elective procedures; 63.6 to 65.7 years for UA/NSTEMI; 62.1 to 63.5 years for STEMI). PCI for ACS (UA/NSTEMI/STEMI) accounted for 61.5% of procedures though this proportion increased from 54.0% to 65.7%.

Figure 1 presents the observed survival rates without adjustment for expected survival. Across all indications mortality risk is highest immediately following the procedures as this is where the survival curve gradient is steepest. The log-rank test indicates some differences in crude survival rates between periods and, typically, more recent years are associated with lower survival. Survival differences by indication and sex are also evident, with survival consistently lower in women. Comparing crude survival from 2007-08 to 2013-14 at one-year: elective procedures, 97.7% to 97.5% for women and 98.0% versus 97.7% for men; UA/STEMI, 94.6% versus 93.7% for women and 95.8% versus 94.4% for men; STEMI, 87.5% versus 86.4% for women and 92.0% versus 91.3% for men (see supplementary Table A2).

Figure 2 shows the relative survival estimate. For elective procedures relative survival levels-off quickly, indicating that procedural mortality risk is present immediately following the procedure but that on average, risk returns to the matched population baseline. For ACS procedures, relative survival continues to decline up to one-year post-procedure, indicating long-term excess risk in these patients compared with the matched population. The log-rank test for relative survival differences reveals smaller differences than for crude survival, though there remains strong evidence of survival differences for men following PCI for UA/STEMI or STEMI. Comparing relative survival from 2007-08 to 2013-14 at one-year: elective procedures, 99.5% to 99.5% for women and 100% versus 99.8% for men; UA/STEMI, 96.7% versus 96.1% for women and 97.8% versus 96.6% for men; STEMI, 89.5% versus 88.6% for women and 93.6% versus 93.0% for men (see supplementary Table A3).

For peer review only

Figure 3 shows the ratio between observed and expected one-year mortality rates by patient age. For younger patients, observed mortality is considerably higher than expected mortality, but this gap reduces with patient age. For instance, observed mortality at one year following elective PCI is 2-5 times greater than expected at age 50 but these are equal by age 80. For UA/NSTEMI, this ratio is between 5-10 at age 50, though is less than 3 by age 80. For STEMI, this ratio decreases from around 9-15 to around 5 from age 50 to 80. There are no clear trends over time.

Confidence intervals at the 95% level for Figures 1, 2, and 3 are provided in supplementary materials (Supplementary Figures A1, A2, A3), as are plots of the cumulative excess hazard rate (Supplementary Figure A4), which may be interpreted as the total additional exposure to mortality risk at any point in time for PCI patients compared with the general population.

Table 1 Number of procedures by period, sex, indication

	2007-2008		2009-2010		2011-2012		2013-2014		2007-2014	
	N	%	N	%	N	%	N	%	N	%
**Female**										
Elective	13847	*45.2*	13392	*38.4*	12603	*33.0*	12093	*31.3*	51935	*36.5*
UA/NSTEMI	12756	*41.6*	13858	*39.8*	15139	*39.6*	15594	*40.4*	57347	*40.3*
STEMI	4043	*13.2*	7608	*21.8*	10480	*27.4*	10908	*28.3*	33039	*23.2*
Total	30646		34858		38222		38595		142321	
**Male**										
Elective	40246	*46.3*	40284	*40.4*	39715	*36.3*	39228	*35.3*	159473	*39.2*
UA/NSTEMI	33797	*38.9*	37049	*37.2*	39379	*36.0*	40749	*36.7*	150974	*37.1*
STEMI	12921	*14.9*	22306	*22.4*	30244	*27.7*	31066	*28.0*	96537	*23.7*
Total	86964		99639		109338		111043		406984	
**Male and Female**										
Elective	54093	*46.0*	53676	*39.9*	52318	*35.5*	51321	*34.3*	211408	*38.5*
UA/NSTEMI	46505	*39.6*	50745	*37.8*	54314	*36.9*	56152	*37.7*	208321	*37.9*
STEMI	16964	*14.4*	29914	*22.2*	40724	*27.6*	41974	*28.1*	129576	*23.6*
Total	117610		134497		147560		149638		549305	

For peer review only

DISCUSSION

We present results for 549,305 PCI procedures over an 8-year period showing observed, expected, and relative survival estimates over time. We demonstrate that, after adjustment for patient age, sex, and procedure date using national life-table mortality rates, differences in patient survival over time are present in men following PCI for ACS, though these differences are small (relative survival at one-year: UA/NSTEMI 97.8% in 2007-2008 vs 96.6% in 2013-2014; STEMI 93.6% in 2007-2008 vs 93.0% in 2013-2014). Small, non-significant relative survival differences were found within other strata. However, the potential counteracting effects of selection factors (that may have increased mortality risk) and of improvements in processes of care (that may have reduced mortality risk) cannot be ruled out.

For elective cases, there is evidence that relative survival increases after the initial post-procedural risk. This suggests that, conditional on surviving the initial risk shock, mortality is on average lower than in the matched population. This is likely driven by patient selection factors, particularly for more elderly patients, where there may be selection bias such that PCI is undertaken in only the healthiest patients. Another factor may be that these patients are highly likely to be supported with secondary prevention treatments for coronary artery disease, whereas in the general population there may be many people with undetected or untreated CAD.

Mortality increases with age at a rate that is lower in the PCI population than in an equivalent matched population. In other words, though mortality is naturally higher as age increases, older patients treated by PCI have a mortality rate more similar to the rate in the general population than younger patients. This is true across all indications. In general then, relative survival is higher for older patients compared with younger patients, though in absolute terms their prognosis is still poorer.

This may partly be determined by clinician selection practices, such that younger patients with CAD are more likely to be referred for PCI than older patients for whom PCI is not optimal. For instance, any 80-year-old considered well enough to undergo elective PCI is likely to have fewer co-morbidities and frailties compared with other 80-year-olds on average. Equally, epidemiological selection suggests that a 40-year-old requiring elective PCI will undoubtedly have a higher baseline risk for mortality than the average 40-year-old since the prevalence of cardiovascular disease is low at this age. For urgent or emergency procedures, simply being part of the PCI registry suggests a degree of patient resilience having experienced an acute, life-threatening event yet survived long enough to be treated.. At the onset of an ACS, as older patients are more likely than younger patients to die before receiving

For peer review only

PCI or any other treatment, those older patients that do receive PCI will typically be less frail than others of a similar age. The distinct trend for higher relative survival in older patients is thus clear evidence of these selection processes at play, and demonstrates the importance of accounting for these imbalances when analysing registry data.

Across all indications, both absolute and relative survival is lower in women than in men. This is a well-known phenomenon in cardiovascular settings variously attributed to differences in age, co-morbidities, time to treatment, secondary prevention.[41–44] This work demonstrates unequivocally that differences in age and background mortality rates do not sufficiently account for this disparity.

In general, both crude and relative survival estimates are lower in recent years. Assuming a consistent data collection methodology over time, realistically this can either be attributed to an average reduction in procedural efficacy or an increase in higher risk procedures that is not solely explained by age- and sex-adjusted background mortality. In the context of rapid changes to PCI patient case-mix and practice in recent years,[45] including more permissive patient selection criteria and increasing use of PCI in favour of alternative revascularisation strategies, the small decline in relative survival observed from 2007 to 2014 is unsurprising. Further adjustment for patient case-mix will demonstrate if and where actual survival gains have been made, or indeed if survival in certain groups of patients has not improved over time.

Relative survival offers distinct advantages over crude survival rates.[19] The relative mortality estimate (one minus relative survival) may be interpreted as an approximation of the excess mortality attributable jointly to the indication for intervention and the intervention itself. This allows PCI-specific mortality to be measured without needing cause of death information which may often be inconsistently recorded or unavailable completely. Relative survival also conveniently accounts for mortality that may be only indirectly related to PCI, for instance non-cardiac mortality secondary to post-operative bleeding.

As the underlying population may vary considerably across different countries, intervention registries or time periods, methods that appropriately adjust for background population mortality provide more suitable survival estimates for comparison in time and space than crude survival alone, provided the comparisons are valid.[46,47] However, despite the popularity of relative survival methodology to compare survival in diseased versus non-diseased populations, in particular cancer, these methods have only recently seen some use in interventional settings like PCI, and relative survival is not routinely reported by PCI registries. This study concisely demonstrates the feasibility and appropriateness of the

For peer review only

relative survival approach in such settings and provides a useful template for producing population-adjusted PCI survival estimates in the future.

Limitations

Patients are matched to national mortality estimates by year, age, and sex only. Despite stratification of models by these factors, substantial case-heterogeneity will remain that is not modelled. No account is taken for other potentially unbalanced factors such as ethnicity, social-economic status, and comorbidities. Accounting for these factors is not possible with the simple relative survival approach used here as national life-tables stratifying by these factors are not readily available. However, using regression modelling in this setting is challenging as the mortality risk following PCI is raised acutely and the hazard rate changes rapidly; modelling the hazard must be done with extreme care as many assumptions of standard regression techniques are unlikely to hold.

Patients will be present more than once in the PCI cohorts if they have been re-admitted for subsequent PCI within the study period. The proportion of procedures in patients already present in the cohort is 13.5% though this is reduced to 7.3% when considering indications separately.

Life-table data is based on national mortality statistics and therefore is not strictly independent of the PCI registry, since all patients will contribute to population and mortality statistics. This is an unavoidable complication in the absence of stratification by CAD or PCI status that biases results towards no relative survival difference. However, given the analysis cohort constitutes less than one percent of the population of England and Wales, this bias can be considered negligible.

As the log-rank tests stratify groups independently it will ignore the inherent correlation between adjacent or near-adjacent years. It does not provide point-estimates of differences between strata as all differences through time contribute to the test statistic. It is therefore most useful when used alongside a graphical representation of survival differences.

Conclusion

We demonstrate that the decline in indication-stratified crude survival rate over time is less apparent after adjusting for expected population mortality matched on age, sex, and procedure year, though patient demographics do not explain all of the difference. Adjusting for additional patient risk factors is necessary to identify other drivers of mortality change.

For peer review only

References

1 Ludman PF. BCIS audit report for 2015 Activity. 2016.https://www.bcis.org.uk/wp-content/uploads/2017/10/BCIS-Audit-2015-data-for-web-with-presentation-ACI-2017-19-10-2017.pdf

2 Desai NR, Bradley SM, Parzynski CS, *et al.* Appropriate Use Criteria for Coronary Revascularization and Trends in Utilization, Patient Selection, and Appropriateness of Percutaneous Coronary Intervention. *JAMA* 2015;**314**:2045. doi:10.1001/jama.2015.13764

3 Masoudi FA, Ponirakis A, de Lemos JA, *et al.* Trends in U.S. Cardiovascular Care: 2016 Report From 4 ACC National Cardiovascular Data Registries. Journal of the American College of Cardiology. 2017;**69**:1427–50. doi:10.1016/j.jacc.2016.12.005

4 Jiménez-Quevedo P, Serrador A, Pérez de Prado A, *et al.* Spanish Cardiac Catheterization and Coronary Intervention Registry. 25th Official Report of the Spanish Society of Cardiology Working Group on Cardiac Catheterization and Interventional Cardiology (1990-2015). *Revista espanola de cardiologia (English ed)* 2016;**69**:1180–9. doi:10.1016/j.rec.2016.08.016

5 Pereira H, Campante Teles R, Costa M, *et al.* Evolução da intervenção coronária percutânea entre 2004-2013. Atividade em Portugal segundo o Registo Nacional de Cardiologia de Intervenção. *Revista Portuguesa de Cardiologia* 2015;**34**:673–81. doi:10.1016/j.repc.2015.06.005

6 Ohlmeier C, Czwikla J, Enders D, *et al.* Perkutane koronare Interventionen. *Bundesgesundheitsblatt - Gesundheitsforschung - Gesundheitsschutz* 2016;**59**:783–8. doi:10.1007/s00103-016-2352-5

7 Laut KG, Gale CP, Pedersen AB, *et al.* Persistent geographical disparities in the use of primary percutaneous coronary intervention in 120 European regions: exploring the variation. *EuroIntervention : journal of EuroPCR in collaboration with the Working Group on Interventional Cardiology of the European Society of Cardiology* 2013;**9**:469–76. doi:10.4244/EIJV9I4A76

8 Smith FG, Brogan RA, Alabas O, *et al.* Comparative care and outcomes for acute coronary syndromes in Central and Eastern European Transitional countries: A review of the literature. *European Heart Journal: Acute Cardiovascular Care* 2015;**4**:537–54.

For peer review only

doi:10.1177/2048872614551545

9 National Institute of Cardiovascular Outcomes Research. Annual reports: findings from the National Audit of Percutaneous Coronary Interventions. 2016.http://www.ucl.ac.uk/nicor/audits/adultpercutaneous/reports (accessed 1 Jan 2017).

10 Masoudi FA, Ponirakis A, Yeh RW, *et al.* Cardiovascular care facts: A report from the national cardiovascular data registry: 2011. *Journal of the American College of Cardiology* 2013;**62**:1931–47. doi:10.1016/j.jacc.2013.05.099

11 National Institute for Clinincal Outcomes Research, Herrett E, Smeeth L, *et al.* Myocardial Ischaemia National Audit Project. Annual Public Report April 2014 - March 2015. *Heart (British Cardiac Society)* 2015;**96**:1264–7. doi:10.1136/hrt.2009.192328

12 Hall M, Laut K, Dondo TB, *et al.* Patient and hospital determinants of primary percutaneous coronary intervention in England, 2003–2013. *Heart* 2016;**102**:313–9. doi:10.1136/heartjnl-2015-308616

13 Bhatnagar P, Wickramasinghe K, Wilkins E, *et al.* Trends in the epidemiology of cardiovascular disease in the UK. *Heart* 2016;**102**:1945–52. doi:10.1136/heartjnl-2016-309573

14 Benjamin EJ, Blaha MJ, Chiuve SE, *et al.* Heart Disease and Stroke Statistics’2017 Update: A Report from the American Heart Association. Circulation. 2017;**135**:e146–603. doi:10.1161/CIR.0000000000000485

15 Banning AP, Baumbach A, Blackman D, *et al.* Percutaneous coronary intervention in the UK: recommendations for good practice 2015. *Heart* 2015;**101**:1–13. doi:10.1136/heartjnl-2015-307821

16 Harold JG, Bass TA, Bashore TM, *et al.* ACCF/AHA/SCAI 2013 Update of the Clinical Competence Statement on Coronary Artery Interventional Procedures. *Catheterization and Cardiovascular Interventions* 2013;**82**:E69–111. doi:10.1002/ccd.24985

17 Authors/Task Force members, Windecker S, Kolh P, *et al.* 2014 ESC/EACTS Guidelines on myocardial revascularization. *European Heart Journal* 2014;**35**:2541–619. doi:10.1093/eurheartj/ehu278

For peer review only

18 Ederer F, Axtell LM, Cutler SJ. The relative survival rate: a statistical methodology. *National Cancer Institute monograph* 1961;**6**:101–21.http://www.ncbi.nlm.nih.gov/pubmed/13889176 (accessed 16 Oct 2017).

19 Lambert PC, Dickman PW, Rutherford MJ. Comparison of different approaches to estimating age standardized net survival. *BMC Medical Research Methodology* 2015;**15**:64. doi:10.1186/s12874-015-0057-3

20 Quaresma M, Coleman MP, Rachet B. 40-year trends in an index of survival for all cancers combined and survival adjusted for age and sex for each cancer in England and Wales, 1971–2011: a population-based study. *The Lancet* 2015;**385**:1206–18. doi:10.1016/S0140-6736(14)61396-9

21 Sarfati D, Blakely T, Pearce N. Measuring cancer survival in populations: Relative survival vs cancer-specific survival. *International Journal of Epidemiology* 2010;**39**:598–610. doi:10.1093/ije/dyp392

22 Nelson CP, Lambert PC, Squire IB, *et al.* Relative survival: what can cardiovascular disease learn from cancer? *European Heart Journal* 2008;**29**:941–7. doi:10.1093/eurheartj/ehn079

23 Martin GP, Sperrin M, Hulme W, *et al.* Relative Survival After Transcatheter Aortic Valve Implantation: How Do Patients Undergoing Transcatheter Aortic Valve Implantation Fare Relative to the General Population? *Journal of the American Heart Association* 2017;**6**:e007229. doi:10.1161/JAHA.117.007229

24 Hall M, Alabas OA, Dondo TB, *et al.* Use of relative survival to evaluate non-ST-elevation myocardial infarction quality of care and clinical outcomes. *European Heart Journal - Quality of Care and Clinical Outcomes* 2015;**1**:85–91. doi:10.1093/ehjqcco/qcv011

25 Velders MA, James SK, Libungan B, *et al.* Prognosis of elderly patients with ST-elevation myocardial infarction treated with primary percutaneous coronary intervention in 2001 to 2011: A report from the Swedish Coronary Angiography and Angioplasty Registry (SCAAR) registry. *American Heart Journal* 2014;**167**:666–73. doi:10.1016/j.ahj.2014.01.013

26 Alabas OA, Brogan RA, Hall M, *et al.* Determinants of excess mortality following unprotected left main stem percutaneous coronary intervention. *Heart* 2016;**102**:1287–95. doi:10.1136/heartjnl-2015-308739

For peer review only

27 Roth C, Gangl C, Dalos D, *et al.* Outcome after elective percutaneous coronary intervention depends on age in patients with stable coronary artery disease - An Analysis of relative survival in a multicenter cohort and an OCT substudy. *PLoS ONE* 2016;**11**. doi:10.1371/journal.pone.0154025

28 Brogan RA, Alabas O, Almudarra S, *et al.* Relative survival and excess mortality following primary percutaneous coronary intervention for ST-elevation myocardial infarction. *European heart journal Acute cardiovascular care* 2017;:2048872617710790. doi:10.1177/2048872617710790

29 Baart SJ, Van Domburg RT, Janssen-Heijnen MLG, *et al.* Impact of Relative Conditional Survival Estimates on Patient Prognosis after Percutaneous Coronary Intervention. *Circulation: Cardiovascular Quality and Outcomes* 2017;**10**. doi:10.1161/CIRCOUTCOMES.116.003344

30 Office for National Statistics. National Life Tables, England & Wales, 1980-82 to 2014-16. 2017.https://www.ons.gov.uk/peoplepopulationandcommunity/birthsdeathsandmarriages/lifeexpectancies/datasets/nationallifetablesenglandandwalesreferencetables (accessed 10 Oct 2017).

31 Dekker FW, de Mutsert R, van Dijk PC, *et al.* Survival analysis: time-dependent effects and time-varying risk factors. *Kidney International* 2008;**74**:994–7. doi:10.1038/ki.2008.328

32 Mantel N. Evaluation of survival data and two new rank order statistics arising in its consideration. *Cancer chemotherapy reports* 1966;**50**:163–70.http://www.ncbi.nlm.nih.gov/pubmed/5910392 (accessed 16 Oct 2017).

33 Peto R, Peto J. Asymptotically Efficient Rank Invariant Test Procedures. *Journal of the Royal Statistical Society Series A (General)* 1972;**135**:185. doi:10.2307/2344317

34 Stare J, Henderson R, Pohar M. An individual measure of relative survival. *Journal of the Royal Statistical Society: Series C (Applied Statistics)* 2005;**54**:115–26. doi:10.1111/j.1467-9876.2005.00473.x

35 R Development Core Team. R: a language and environment for statistical computing. [2.12.2]. 2011.http://www.r-project.org/.

For peer review only

36 Wickham H. Easily Install and Load ‘Tidyverse’ Packages [R package tidyverse version 1.1.1]. https://cran.r-project.org/web/packages/tidyverse/index.html (accessed 21 Mar 2017).

37 Perme MP. relsurv: Relative Survival. 2017.https://cran.r-project.org/package=relsurv

38 Pohar M, Stare J. Making relative survival analysis relatively easy. *Computers in Biology and Medicine* 2007;**37**:1741–9. doi:10.1016/j.compbiomed.2007.04.010

39 Pohar M, Stare J. Relative survival analysis in R. *Computer Methods and Programs in Biomedicine* 2006;**81**:272–8. doi:10.1016/j.cmpb.2006.01.004

40 Hulme W. BCIS-PCI-relative-survival GitHub repository. https://github.com/wjchulme/BCIS-PCI-relative-survival (accessed 4 May 2018).

41 Heer T, Hochadel M, Schmidt K, *et al.* Sex Differences in Percutaneous Coronary Intervention-Insights From the Coronary Angiography and PCI Registry of the German Society of Cardiology. *Journal of the American Heart Association* 2017;**6**:e004972. doi:10.1161/JAHA.116.004972

42 Raphael CE, Singh M, Bell M, *et al.* Sex Differences in Long-Term Cause-Specific Mortality After Percutaneous Coronary Intervention: Temporal Trends and Mechanisms. *Circulation Cardiovascular interventions* 2018;**11**:e006062. doi:10.1161/CIRCINTERVENTIONS.117.006062

43 Kunadian V, Qiu W, Lagerqvist B, *et al.* Gender Differences in Outcomes and Predictors of All-Cause Mortality After Percutaneous Coronary Intervention (Data from United Kingdom and Sweden). *American Journal of Cardiology* 2017;**119**:210–6. doi:10.1016/j.amjcard.2016.09.052

44 Birkemeyer RG, Schneider H, Rillig A, *et al.* Do gender differences in primary PCI mortality represent a different use of guideline adherent therapy? *European Heart Journal* 2013;**34**:P2227–P2227. doi:10.1093/eurheartj/eht308.P2227

45 Ludman P. BCIS Audit Report for 2014 Activity. 2015.https://www.bcis.org.uk/wp-content/uploads/2017/01/BCIS-audit-2014.pdf

46 Pokhrel A, Hakulinen T. How to interpret the relative survival ratios of cancer patients. *European Journal of Cancer* 2008;**44**:2661–7. doi:10.1016/j.ejca.2008.08.016

For peer review only

47 Rutherford MJ, Dickman PW, Lambert PC. Comparison of methods for calculating relative survival in population-based studies. *Cancer Epidemiology* 2012;**36**:16–21. doi:10.1016/j.canep.2011.05.010

For peer review only

Author Statement WH and MM conceived the study. WH, GM, MS, and EK participated in the design of the study. WH performed the statistical analysis and drafted the manuscript. WH, GM, MS, EK, NC, PL, and MM interpreted the data and results, made important contributions and revisions to the work, and read and approved the final manuscript.

Data sharing Analysis scripts and a synthetic test dataset are available on GitHub: https://zenodo.org/badge/latestdoi/131696652.

Funding This work was supported by MRC grant number MR/K006665/1.

Disclosures None.

For peer review only

Figure legends

Figure 1 Observed survival rates by patient indication, and year of procedure. P-values are from the log-rank test.

Figure 2 Relative survival estimates by patient indication, and year of procedure. P-values are from a log-rank-type test for relative survival curves.

Figure 3 Observed-to-expected one-year mortality ratio (log-scale) by patient age. Binomial General Additive Models with 7-knot splines are used for smoothing the observed one-year mortality, then divided by the expected mortality rate. 95% confidence limits are removed for clarity.

For peer review only

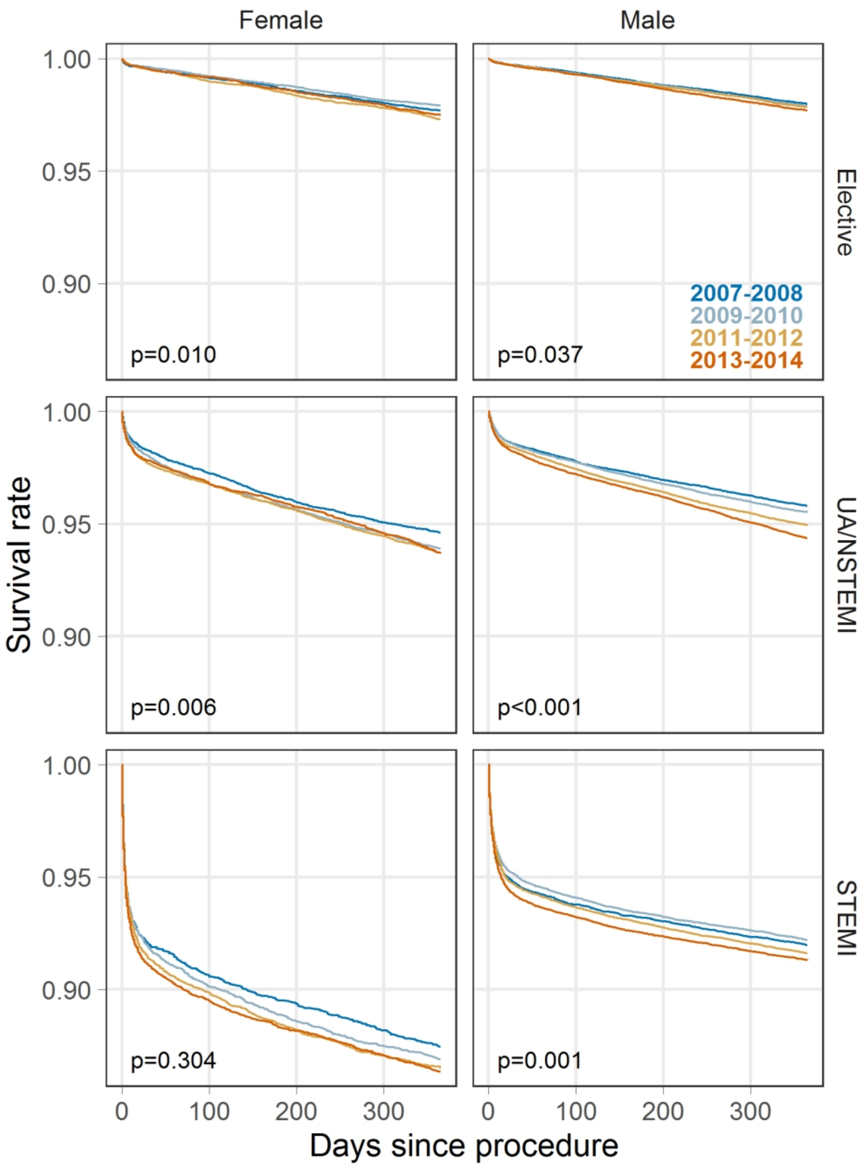

Observed survival rates by patient indication, and year of procedure. P-values are from the log-rank test.

149x199mm (300 x 300 DPI)

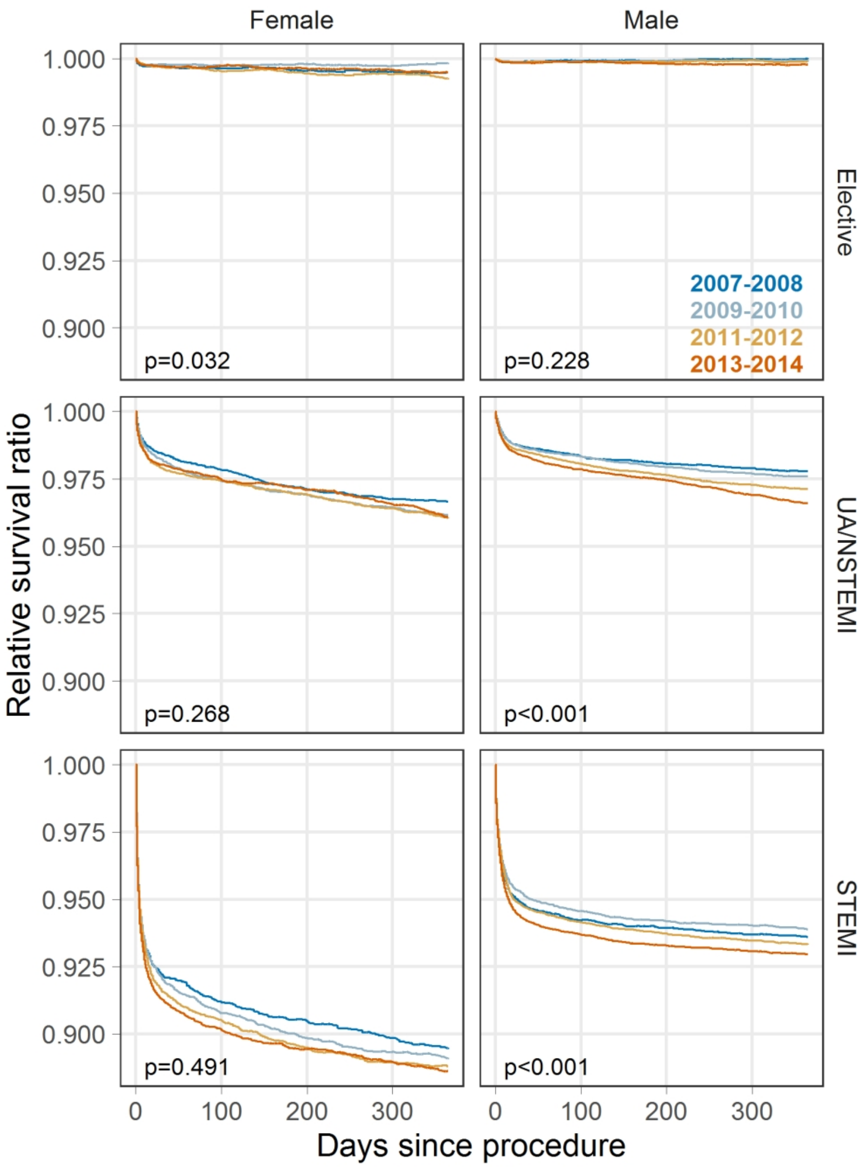

Relative survival estimates by patient indication, and year of procedure. P-values are from a log-rank-type test for relative survival curves.

149x199mm (300 x 300 DPI)

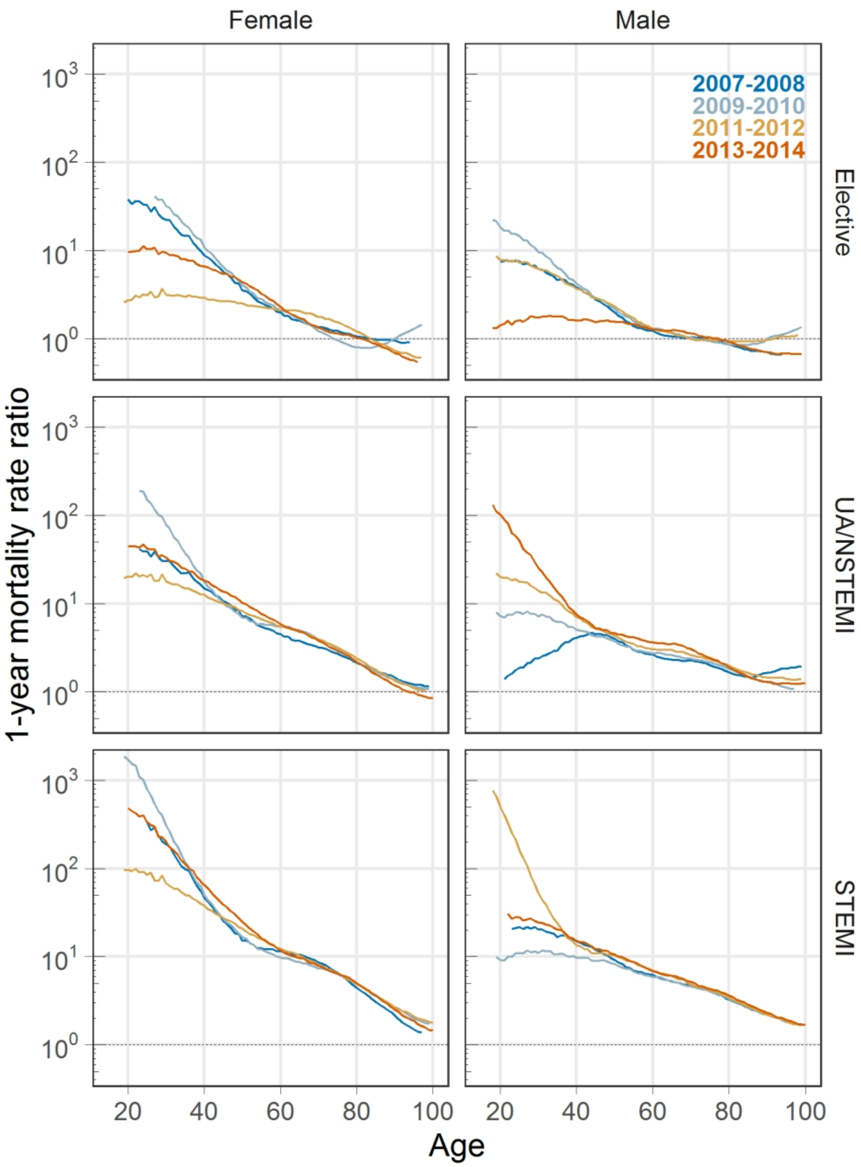

Observed-to-expected one-year mortality ratio (log-scale) by patient age.

149x199mm (300 x 300 DPI)

Supplementary Materials

Table A1 Procedure selection

	Procedures excluded		Procedures remaining	
	**N**	**Cumul. N**	**N**	**%**
Interventional procedures in England and Wales NHS, 2007 – 2014	-	-	575203	100.0
Exclude missing age and age<18 and age>100	439	439	574764	99.9
Exclude missing sex	984	1423	573780	99.8
Exclude missing indication and rescue/bail-out PCI	3887	5310	569893	99.1
Exclude missing mortality	20588	25898	549305	95.5

For peer review only

Table A2a Baseline patient characteristics for Elective procedures

		Female				Male			
		2007-2008	2009-2010	2011-2012	2013-2014	2007-2008	2009-2010	2011-2012	2013-2014
**Number of procedures**		13847	13392	12603	12093	40246	40284	39715	39228
**Mean age, years (SD)**		67.1 (10.0)	67.8 (10.3)	68.3 (10.5)	68.5 (10.6)	63.7 (10.2)	64.2 (10.5)	64.6 (10.6)	64.9 (10.8)
**Variable, N (%)**									
Age	<50	796 (5.7)	738 (5.5)	636 (5.0)	624 (5.2)	3723 (9.3)	3702 (9.2)	3451 (8.7)	3325 (8.5)
	50s	2229 (16.1)	2034 (15.2)	1940 (15.4)	1854 (15.3)	9940 (24.7)	9289 (23.1)	9021 (22.7)	8961 (22.8)
	60s	4641 (33.5)	4193 (31.3)	3871 (30.7)	3563 (29.5)	14270 (35.5)	14097 (35.0)	13734 (34.6)	13249 (33.8)
	70s	4942 (35.7)	4820 (36.0)	4364 (34.6)	4235 (35.0)	10197 (25.3)	10486 (26.0)	10354 (26.1)	10191 (26.0)
	80+	1239 (8.9)	1607 (12.0)	1792 (14.2)	1817 (15.0)	2116 (5.3)	2710 (6.7)	3155 (7.9)	3502 (8.9)
Ethnicity	Asian	482 (5.1)	628 (6.0)	614 (6.2)	614 (6.4)	1777 (6.6)	2127 (6.8)	2329 (7.4)	2256 (7.3)
	Black	80 (0.9)	90 (0.9)	108 (1.1)	100 (1.0)	153 (0.6)	201 (0.6)	190 (0.6)	228 (0.7)
	White	7881 (84.2)	9216 (87.3)	8463 (85.2)	8045 (83.7)	22368 (82.5)	27231 (86.5)	26526 (84.3)	25584 (82.7)
	Other	918 (9.8)	619 (5.9)	748 (7.5)	851 (8.9)	2817 (10.4)	1908 (6.1)	2431 (7.7)	2863 (9.3)
Smoking	Never	5744 (49.5)	6037 (50.2)	6028 (53.1)	5722 (52.6)	11344 (33.5)	12089 (33.6)	12872 (36.2)	12941 (36.6)
	Ex-smoker	4368 (37.7)	4477 (37.2)	3944 (34.8)	3933 (36.1)	17927 (53.0)	19089 (53.0)	18131 (51.0)	18051 (51.1)
	Current	1487 (12.8)	1517 (12.6)	1376 (12.1)	1231 (11.3)	4547 (13.4)	4828 (13.4)	4538 (12.8)	4360 (12.3)
Dialysis		101 (0.7)	82 (0.6)	85 (0.7)	119 (1.0)	299 (0.7)	294 (0.7)	289 (0.7)	403 (1.0)
Diabetes		2736 (20.8)	2839 (21.7)	2809 (23.3)	2739 (23.5)	7466 (19.5)	8258 (21.1)	8361 (22.1)	8847 (23.5)
Previous PCI		2933 (22.7)	3600 (27.6)	3760 (30.6)	4043 (34.1)	10412 (27.8)	13404 (34.2)	14590 (37.7)	15981 (41.5)
Previous CABG		1336 (15.7)	850 (8.7)	810 (8.4)	766 (8.2)	5169 (20.5)	4116 (14.1)	4216 (13.6)	4161 (13.6)
Previous MI		3174 (27.1)	3640 (30.0)	3324 (28.4)	3267 (28.5)	12216 (35.9)	13370 (36.9)	13270 (36.2)	13861 (37.3)
Previous CVA		463 (3.5)	558 (4.3)	531 (4.4)	461 (4.0)	1149 (3.0)	1445 (3.7)	1403 (3.7)	1377 (3.7)
High cholesterol		8050 (61.6)	8780 (68.0)	7889 (65.4)	7464 (64.9)	22880 (60.3)	26255 (67.5)	24512 (64.8)	24058 (64.6)
Hypertension		7759 (59.4)	8633 (66.8)	8190 (67.9)	7803 (67.8)	19934 (52.6)	23528 (60.5)	23209 (61.4)	23498 (63.1)
Peripheral Vascular Disease		589 (4.5)	670 (5.2)	677 (5.6)	583 (5.1)	1739 (4.6)	2208 (5.7)	2103 (5.6)	1947 (5.2)
Valvular Heart Disease		163 (1.2)	236 (1.8)	324 (2.7)	330 (2.9)	318 (0.8)	534 (1.4)	660 (1.7)	776 (2.1)
LVEF	Good (>50%)	6106 (84.2)	6684 (83.3)	6158 (84.9)	6043 (84.3)	16388 (78.8)	18962 (78.2)	18424 (79.7)	18687 (79.4)
	Fair (30-50%)	871 (12.0)	1109 (13.8)	938 (12.9)	942 (13.1)	3338 (16.1)	4268 (17.6)	3851 (16.7)	3985 (16.9)
	Poor (<30%)	277 (3.8)	228 (2.8)	161 (2.2)	182 (2.5)	1069 (5.1)	1007 (4.2)	838 (3.6)	866 (3.7)
Chronic total occlusions		1396 (10.9)	1348 (11.2)	1203 (10.5)	1168 (10.5)	5159 (13.8)	5438 (14.8)	5239 (14.4)	5266 (14.4)
Access	Femoral only	9531 (74.2)	8080 (61.7)	6070 (48.9)	4348 (36.5)	26587 (71.0)	22678 (57.4)	16738 (42.8)	11676 (30.2)
	Radial only	3146 (24.5)	4724 (36.1)	5959 (48.0)	7073 (59.3)	10475 (28.0)	15995 (40.5)	21129 (54.0)	25454 (65.7)
	Multiple	118 (0.9)	252 (1.9)	321 (2.6)	456 (3.8)	298 (0.8)	650 (1.6)	1093 (2.8)	1456 (3.8)
	Other	48 (0.4)	48 (0.4)	55 (0.4)	46 (0.4)	80 (0.2)	154 (0.4)	142 (0.4)	137 (0.4)
LMS vessel PCI		345 (2.5)	439 (3.3)	462 (3.7)	597 (4.9)	1186 (2.9)	1371 (3.4)	1588 (4.0)	2164 (5.5)
Multivessel PCI		2080 (15.0)	2053 (15.3)	1832 (14.5)	1763 (14.6)	6966 (17.3)	6821 (16.9)	6751 (17.0)	6360 (16.2)

For peer review only

Table A2b Baseline patient characteristics for UA/NSTEMI procedures

		Female				Male			
		2007-2008	2009-2010	2011-2012	2013-2014	2007-2008	2009-2010	2011-2012	2013-2014
**Number of procedures**		12756	13858	15139	15594	33797	37049	39379	40749
**Mean age, years (SD)**		67.3 (11.5)	68.4 (11.9)	68.9 (12.2)	69.1 (12.2)	62.3 (11.8)	63.2 (11.9)	64.0 (12.2)	64.5 (12.3)
**Variable, N (%)**									
Age	<50	1072 (8.4)	1054 (7.6)	1193 (7.9)	1163 (7.5)	5161 (15.3)	5181 (14.0)	5069 (12.9)	4941 (12.1)
	50s	2009 (15.7)	2149 (15.5)	2199 (14.5)	2282 (14.6)	8787 (26.0)	9093 (24.5)	9418 (23.9)	9644 (23.7)
	60s	3665 (28.7)	3546 (25.6)	3767 (24.9)	3931 (25.2)	9925 (29.4)	10792 (29.1)	11207 (28.5)	11524 (28.3)
	70s	4228 (33.1)	4546 (32.8)	4764 (31.5)	4825 (30.9)	7452 (22.0)	8602 (23.2)	9195 (23.4)	9555 (23.4)
	80+	1782 (14.0)	2563 (18.5)	3216 (21.2)	3393 (21.8)	2472 (7.3)	3381 (9.1)	4490 (11.4)	5085 (12.5)
Ethnicity	Asian	510 (5.7)	565 (5.1)	617 (5.1)	792 (6.5)	1513 (6.4)	1848 (6.3)	2055 (6.6)	2317 (7.3)
	Black	65 (0.7)	102 (0.9)	136 (1.1)	139 (1.1)	163 (0.7)	240 (0.8)	242 (0.8)	280 (0.9)
	White	7764 (86.1)	9829 (88.2)	10607 (87.4)	10345 (84.9)	20211 (85.2)	25772 (87.3)	26894 (85.8)	26859 (84.3)
	Other	676 (7.5)	645 (5.8)	778 (6.4)	910 (7.5)	1847 (7.8)	1647 (5.6)	2158 (6.9)	2388 (7.5)
Smoking	Never	4403 (41.0)	5405 (43.9)	6332 (46.1)	6709 (47.0)	7547 (26.2)	9068 (27.2)	10987 (30.6)	11925 (31.7)
	Ex-smoker	3563 (33.2)	3855 (31.3)	4240 (30.9)	4459 (31.2)	12572 (43.7)	14408 (43.2)	15068 (41.9)	16149 (42.9)
	Current	2773 (25.8)	3058 (24.8)	3161 (23.0)	3116 (21.8)	8653 (30.1)	9860 (29.6)	9865 (27.5)	9603 (25.5)
Dialysis		140 (1.1)	139 (1.0)	165 (1.1)	215 (1.4)	365 (1.1)	364 (1.0)	452 (1.1)	523 (1.3)
Diabetes		2358 (19.5)	2858 (21.3)	3438 (23.6)	3777 (25.1)	5305 (16.6)	6561 (18.2)	7868 (20.8)	9027 (22.9)
Previous PCI		1741 (14.7)	2180 (16.2)	2555 (17.3)	3008 (19.7)	5000 (15.9)	6356 (17.7)	8101 (21.1)	8959 (22.4)
Previous CABG		891 (11.3)	748 (7.5)	778 (6.7)	816 (6.9)	3161 (14.8)	2781 (10.4)	3450 (11.3)	3461 (11.1)
Previous MI		3080 (28.9)	3298 (26.8)	3639 (25.8)	3833 (26.1)	8909 (31.6)	9730 (29.4)	11017 (30.1)	11336 (29.6)
Previous CVA		507 (4.2)	750 (5.6)	827 (5.7)	822 (5.5)	1074 (3.3)	1616 (4.5)	1777 (4.7)	1916 (4.9)
High cholesterol		7164 (58.9)	8344 (61.9)	8586 (58.9)	8454 (56.7)	18157 (56.5)	21576 (60.0)	21564 (56.9)	21501 (55.1)
Hypertension		6982 (57.4)	8447 (62.7)	9129 (62.7)	9540 (64.0)	15573 (48.5)	19115 (53.2)	20669 (54.5)	21892 (56.1)
Peripheral Vascular Disease		599 (4.9)	775 (5.8)	858 (5.9)	895 (6.0)	1486 (4.6)	2027 (5.6)	2232 (5.9)	2472 (6.3)
Valvular Heart Disease		118 (1.0)	207 (1.5)	301 (2.1)	335 (2.2)	223 (0.7)	328 (0.9)	542 (1.4)	684 (1.8)
LVEF	Good (>50%)	4523 (69.1)	5000 (67.4)	5340 (69.1)	5569 (68.0)	11530 (66.7)	12756 (64.8)	13090 (67.0)	13985 (65.9)
	Fair (30-50%)	1638 (25.0)	1982 (26.7)	1907 (24.7)	2102 (25.7)	4557 (26.4)	5540 (28.2)	5047 (25.8)	5570 (26.3)
	Poor (<30%)	382 (5.8)	440 (5.9)	478 (6.2)	520 (6.3)	1190 (6.9)	1378 (7.0)	1408 (7.2)	1652 (7.8)
Chronic total occlusions		647 (5.5)	578 (4.6)	623 (4.5)	572 (3.9)	1886 (6.1)	1836 (5.4)	1867 (5.2)	2007 (5.3)
Shock		135 (1.1)	175 (1.3)	205 (1.4)	217 (1.4)	319 (1.0)	417 (1.1)	475 (1.2)	605 (1.5)
Ventilated		95 (0.9)	104 (0.9)	168 (1.2)	163 (1.1)	218 (0.8)	327 (1.0)	449 (1.2)	530 (1.4)
IABP		177 (1.6)	192 (1.5)	211 (1.4)	160 (1.1)	398 (1.3)	443 (1.3)	471 (1.2)	395 (1.0)
Cardio-pulmonary support		4 (0.0)	9 (0.1)	10 (0.1)	7 (0.0)	11 (0.0)	21 (0.1)	17 (0.0)	19 (0.0)
Inotropes		47 (0.4)	71 (0.6)	77 (0.5)	77 (0.5)	113 (0.4)	149 (0.4)	163 (0.4)	231 (0.6)
Access	Femoral only	8724 (72.1)	7538 (55.4)	6202 (41.5)	4681 (30.4)	21963 (68.5)	18419 (50.6)	13905 (35.8)	10146 (25.2)
	Radial only	3209 (26.5)	5740 (42.2)	8236 (55.1)	10106 (65.7)	9759 (30.4)	17346 (47.7)	24030 (61.9)	28948 (72.0)
	Multiple	134 (1.1)	302 (2.2)	456 (3.1)	546 (3.6)	294 (0.9)	563 (1.5)	801 (2.1)	1019 (2.5)
	Other	39 (0.3)	34 (0.2)	48 (0.3)	46 (0.3)	57 (0.2)	74 (0.2)	90 (0.2)	86 (0.2)
LMS vessel PCI		434 (3.4)	594 (4.3)	749 (4.9)	807 (5.2)	971 (2.9)	1192 (3.2)	1607 (4.1)	2187 (5.4)
Multivessel PCI		2110 (16.5)	2159 (15.6)	2307 (15.2)	2394 (15.4)	5825 (17.2)	5951 (16.1)	6248 (15.9)	6772 (16.6)

For peer review only

Table A2c Baseline patient characteristics for STEMI procedures

		Female				Male			
		2007-2008	2009-2010	2011-2012	2013-2014	2007-2008	2009-2010	2011-2012	2013-2014
**Number of procedures**		4043	7608	10480	10908	12921	22306	30244	31066
**Mean age, years (SD)**		67.1 (12.7)	68.4 (12.9)	68.7 (13.2)	68.6 (13.3)	60.5 (12.3)	61.2 (12.4)	61.6 (12.6)	61.8 (12.4)
**Variable, N (%)**									
Age	<50	422 (10.4)	699 (9.2)	958 (9.1)	1035 (9.5)	2586 (20.0)	4178 (18.7)	5429 (18.0)	5350 (17.2)
	50s	678 (16.8)	1207 (15.9)	1653 (15.8)	1754 (16.1)	3545 (27.4)	6091 (27.3)	8176 (27.0)	8532 (27.5)
	60s	1042 (25.8)	1866 (24.5)	2539 (24.2)	2629 (24.1)	3564 (27.6)	6079 (27.3)	8360 (27.6)	8736 (28.1)
	70s	1202 (29.7)	2179 (28.6)	2898 (27.7)	2879 (26.4)	2364 (18.3)	4157 (18.6)	5509 (18.2)	5610 (18.1)
	80+	699 (17.3)	1657 (21.8)	2432 (23.2)	2611 (23.9)	862 (6.7)	1801 (8.1)	2770 (9.2)	2838 (9.1)
Ethnicity	Asian	164 (5.4)	269 (4.3)	397 (4.4)	389 (4.3)	802 (8.3)	1296 (7.2)	1845 (7.1)	1801 (7.0)
	Black	27 (0.9)	41 (0.7)	67 (0.7)	74 (0.8)	102 (1.1)	144 (0.8)	193 (0.7)	226 (0.9)
	White	2674 (88.0)	5705 (91.6)	8150 (90.8)	8221 (90.8)	8110 (84.4)	15982 (88.2)	22654 (87.5)	22384 (87.1)
	Other	173 (5.7)	212 (3.4)	363 (4.0)	368 (4.1)	591 (6.2)	696 (3.8)	1185 (4.6)	1279 (5.0)
Smoking	Never	1359 (39.7)	2603 (38.6)	3991 (43.1)	4167 (43.2)	2910 (26.1)	5470 (27.4)	8538 (31.4)	8956 (32.0)
	Ex-smoker	790 (23.1)	1542 (22.9)	1904 (20.6)	2155 (22.3)	3530 (31.7)	5900 (29.6)	7607 (28.0)	8085 (28.8)
	Current	1278 (37.3)	2593 (38.5)	3357 (36.3)	3325 (34.5)	4690 (42.1)	8575 (43.0)	11042 (40.6)	10989 (39.2)
Dialysis		32 (0.8)	40 (0.5)	38 (0.4)	46 (0.4)	79 (0.6)	85 (0.4)	125 (0.4)	127 (0.4)
Diabetes		639 (16.7)	1091 (14.8)	1557 (15.6)	1744 (16.9)	1609 (13.1)	2681 (12.4)	3877 (13.5)	4320 (14.6)
Previous PCI		314 (8.3)	551 (7.4)	751 (7.4)	798 (7.5)	1287 (10.7)	2233 (10.3)	2938 (10.0)	3401 (11.2)
Previous CABG		195 (8.7)	154 (2.9)	256 (3.3)	269 (3.4)	707 (9.8)	727 (4.7)	959 (4.2)	1033 (4.6)
Previous MI		584 (16.3)	840 (12.1)	1037 (10.7)	1074 (10.6)	2150 (18.8)	2976 (14.7)	3761 (13.4)	4021 (13.9)
Previous CVA		157 (4.1)	362 (5.0)	461 (4.6)	487 (4.7)	375 (3.1)	699 (3.3)	984 (3.4)	975 (3.3)
High cholesterol		1695 (44.5)	3142 (43.0)	3997 (40.0)	3987 (38.9)	5091 (41.8)	9063 (42.3)	11286 (39.2)	11200 (38.3)
Hypertension		1838 (48.3)	3503 (47.9)	4776 (47.8)	5013 (48.9)	4649 (38.2)	8427 (39.4)	11215 (39.0)	11724 (40.1)
Peripheral Vascular Disease		140 (3.7)	253 (3.5)	374 (3.7)	395 (3.9)	420 (3.4)	657 (3.1)	953 (3.3)	952 (3.3)
Valvular Heart Disease		27 (0.7)	47 (0.6)	131 (1.3)	107 (1.0)	44 (0.4)	80 (0.4)	198 (0.7)	172 (0.6)
LVEF	Good (>50%)	648 (47.3)	1136 (47.9)	1669 (53.1)	1572 (47.9)	2183 (49.7)	3452 (48.0)	4926 (53.9)	4749 (49.1)
	Fair (30-50%)	556 (40.6)	957 (40.4)	1183 (37.7)	1338 (40.7)	1560 (35.5)	2883 (40.0)	3380 (37.0)	3842 (39.8)
	Poor (<30%)	166 (12.1)	277 (11.7)	290 (9.2)	375 (11.4)	646 (14.7)	864 (12.0)	830 (9.1)	1073 (11.1)
Chronic total occlusions		172 (4.6)	269 (3.9)	218 (2.3)	239 (2.4)	662 (5.5)	855 (4.2)	705 (2.6)	769 (2.7)
Shock		325 (8.2)	526 (7.0)	851 (8.2)	1055 (9.8)	811 (6.4)	1364 (6.2)	1973 (6.6)	2482 (8.1)
Ventilated		113 (3.3)	198 (3.0)	371 (4.0)	458 (4.7)	318 (2.9)	622 (3.2)	1201 (4.5)	1477 (5.3)
IABP		272 (7.3)	391 (5.7)	478 (4.8)	381 (3.6)	718 (6.0)	1117 (5.5)	1334 (4.7)	1090 (3.6)
Cardio-pulmonary support		10 (0.3)	23 (0.3)	44 (0.4)	28 (0.3)	30 (0.3)	57 (0.3)	99 (0.3)	82 (0.3)
Inotropes		123 (3.3)	178 (2.6)	268 (2.7)	323 (3.1)	272 (2.3)	428 (2.1)	579 (2.0)	798 (2.7)
Access	Femoral only	2942 (75.9)	4441 (59.5)	4573 (44.3)	3406 (31.7)	8928 (71.9)	11990 (54.6)	11216 (37.7)	7580 (24.8)
	Radial only	868 (22.4)	2776 (37.2)	5385 (52.1)	6881 (63.9)	3314 (26.7)	9483 (43.2)	17852 (60.0)	22168 (72.4)
	Multiple	60 (1.5)	237 (3.2)	367 (3.6)	449 (4.2)	147 (1.2)	439 (2.0)	658 (2.2)	806 (2.6)
	Other	6 (0.2)	11 (0.1)	9 (0.1)	24 (0.2)	22 (0.2)	32 (0.1)	45 (0.2)	44 (0.1)
LMS vessel PCI		81 (2.0)	145 (1.9)	219 (2.1)	330 (3.0)	249 (1.9)	423 (1.9)	638 (2.1)	1097 (3.5)
Multivessel PCI		327 (8.1)	458 (6.0)	576 (5.5)	619 (5.7)	1066 (8.3)	1432 (6.4)	1831 (6.1)	2031 (6.5)

For peer review only

Table A3 Crude and relative survival estimates with 95% confidence limits

Year	30-days				365-days			
	Crude survival	Crude survival 95% CI	Relative survival	Relative survival 95% CI	Crude survival	Crude survival 95% CI	Relative survival	Relative survival 95% CI
**Female, Elective**								
**2007-2008**	0.996	(0.994, 0.997)	0.997	(0.996, 0.998)	0.977	(0.974, 0.979)	0.995	(0.992, 0.997)
**2009-2010**	0.996	(0.995, 0.997)	0.998	(0.997, 0.999)	0.979	(0.977, 0.981)	0.998	(0.996, 1.001)
**2011-2012**	0.995	(0.994, 0.996)	0.997	(0.995, 0.998)	0.973	(0.970, 0.976)	0.993	(0.990, 0.996)
**2013-2014**	0.995	(0.994, 0.997)	0.997	(0.996, 0.998)	0.975	(0.972, 0.978)	0.995	(0.992, 0.998)
**Female, UA/NSTEMI**								
**2007-2008**	0.983	(0.980, 0.985)	0.985	(0.982, 0.987)	0.946	(0.942, 0.950)	0.967	(0.963, 0.971)
**2009-2010**	0.980	(0.978, 0.983)	0.982	(0.980, 0.985)	0.939	(0.935, 0.943)	0.962	(0.957, 0.966)
**2011-2012**	0.977	(0.974, 0.979)	0.979	(0.977, 0.981)	0.937	(0.933, 0.941)	0.960	(0.956, 0.964)
**2013-2014**	0.978	(0.976, 0.980)	0.980	(0.978, 0.982)	0.937	(0.933, 0.941)	0.961	(0.957, 0.965)
**Female, STEMI**								
**2007-2008**	0.920	(0.912, 0.928)	0.922	(0.913, 0.930)	0.875	(0.864, 0.884)	0.895	(0.884, 0.905)
**2009-2010**	0.919	(0.913, 0.925)	0.921	(0.915, 0.927)	0.869	(0.861, 0.876)	0.891	(0.883, 0.899)
**2011-2012**	0.914	(0.908, 0.919)	0.916	(0.910, 0.921)	0.865	(0.858, 0.871)	0.888	(0.881, 0.895)
**2013-2014**	0.911	(0.905, 0.916)	0.913	(0.907, 0.918)	0.864	(0.857, 0.870)	0.886	(0.879, 0.893)
**Male, Elective**								
**2007-2008**	0.997	(0.997, 0.998)	0.999	(0.998, 0.999)	0.980	(0.978, 0.981)	1.000	(0.998, 1.001)
**2009-2010**	0.997	(0.996, 0.998)	0.999	(0.998, 0.999)	0.979	(0.977, 0.980)	0.999	(0.998, 1.001)
**2011-2012**	0.997	(0.996, 0.997)	0.999	(0.998, 0.999)	0.978	(0.977, 0.980)	0.999	(0.997, 1.000)
**2013-2014**	0.997	(0.996, 0.997)	0.998	(0.998, 0.999)	0.977	(0.975, 0.978)	0.998	(0.996, 0.999)
**Male, UA/NSTEMI**								
**2007-2008**	0.985	(0.984, 0.987)	0.987	(0.986, 0.988)	0.958	(0.956, 0.960)	0.978	(0.976, 0.980)
**2009-2010**	0.985	(0.984, 0.986)	0.987	(0.986, 0.988)	0.955	(0.953, 0.957)	0.976	(0.974, 0.978)
**2011-2012**	0.984	(0.982, 0.985)	0.986	(0.984, 0.987)	0.949	(0.947, 0.952)	0.971	(0.969, 0.973)
**2013-2014**	0.982	(0.981, 0.983)	0.984	(0.982, 0.985)	0.944	(0.941, 0.946)	0.966	(0.963, 0.968)
**Male, STEMI**								
**2007-2008**	0.947	(0.943, 0.951)	0.949	(0.945, 0.953)	0.920	(0.915, 0.924)	0.936	(0.931, 0.941)
**2009-2010**	0.951	(0.948, 0.954)	0.953	(0.950, 0.955)	0.922	(0.918, 0.925)	0.939	(0.935, 0.942)
**2011-2012**	0.947	(0.944, 0.949)	0.948	(0.945, 0.951)	0.916	(0.913, 0.919)	0.933	(0.930, 0.936)
**2013-2014**	0.942	(0.939, 0.944)	0.943	(0.941, 0.946)	0.913	(0.910, 0.916)	0.930	(0.926, 0.933)

For peer review only

Figure A1 Observed survival rates by patient age, indication, and year of procedure, with 95% confidence limits. P-values from the log-rank test.

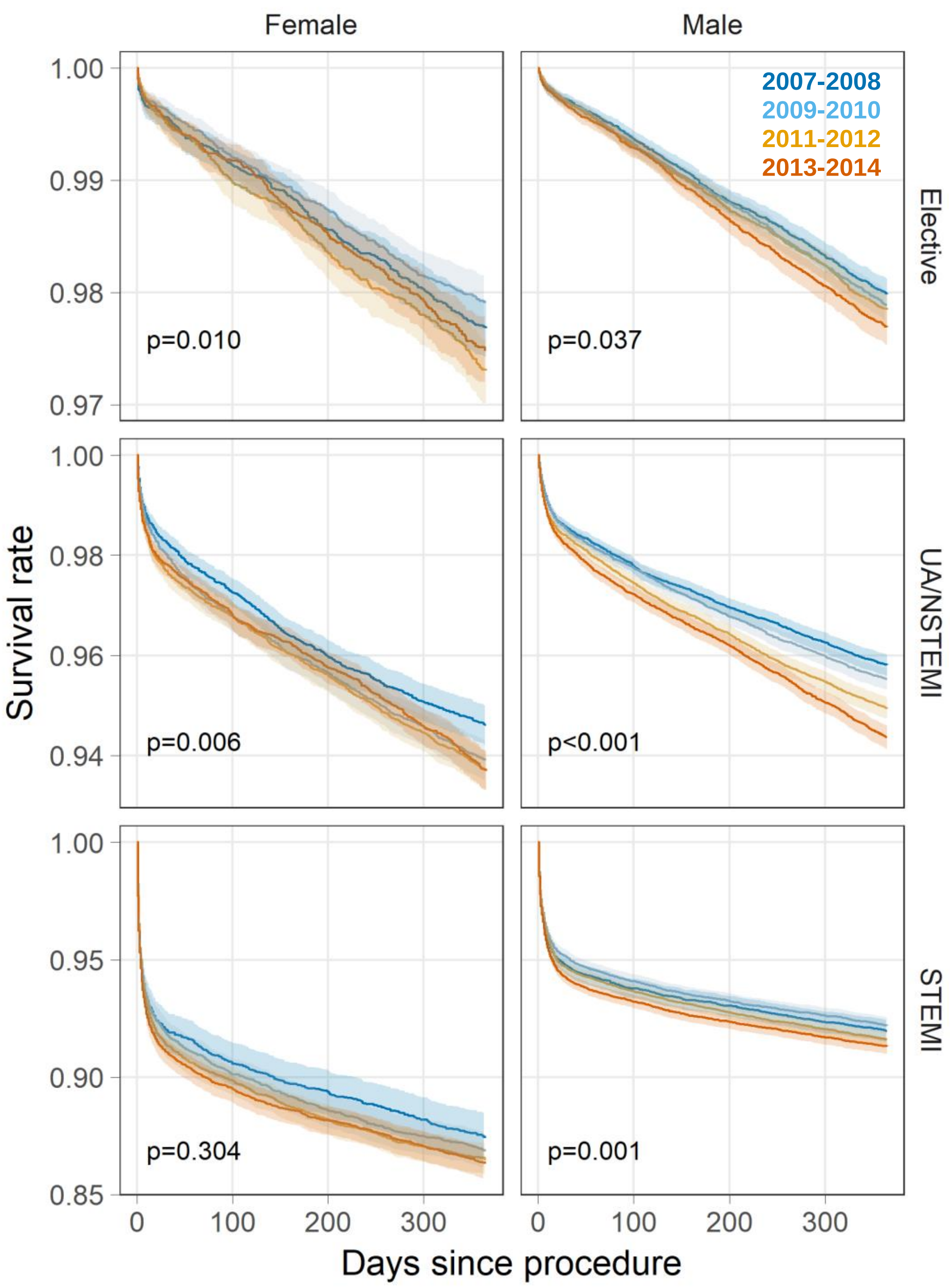

Figure A2 Relative survival estimates by patient age, indication, and year of procedure, with 95% confidence limits. P-values from a log-rank-type test for relative survival curves.

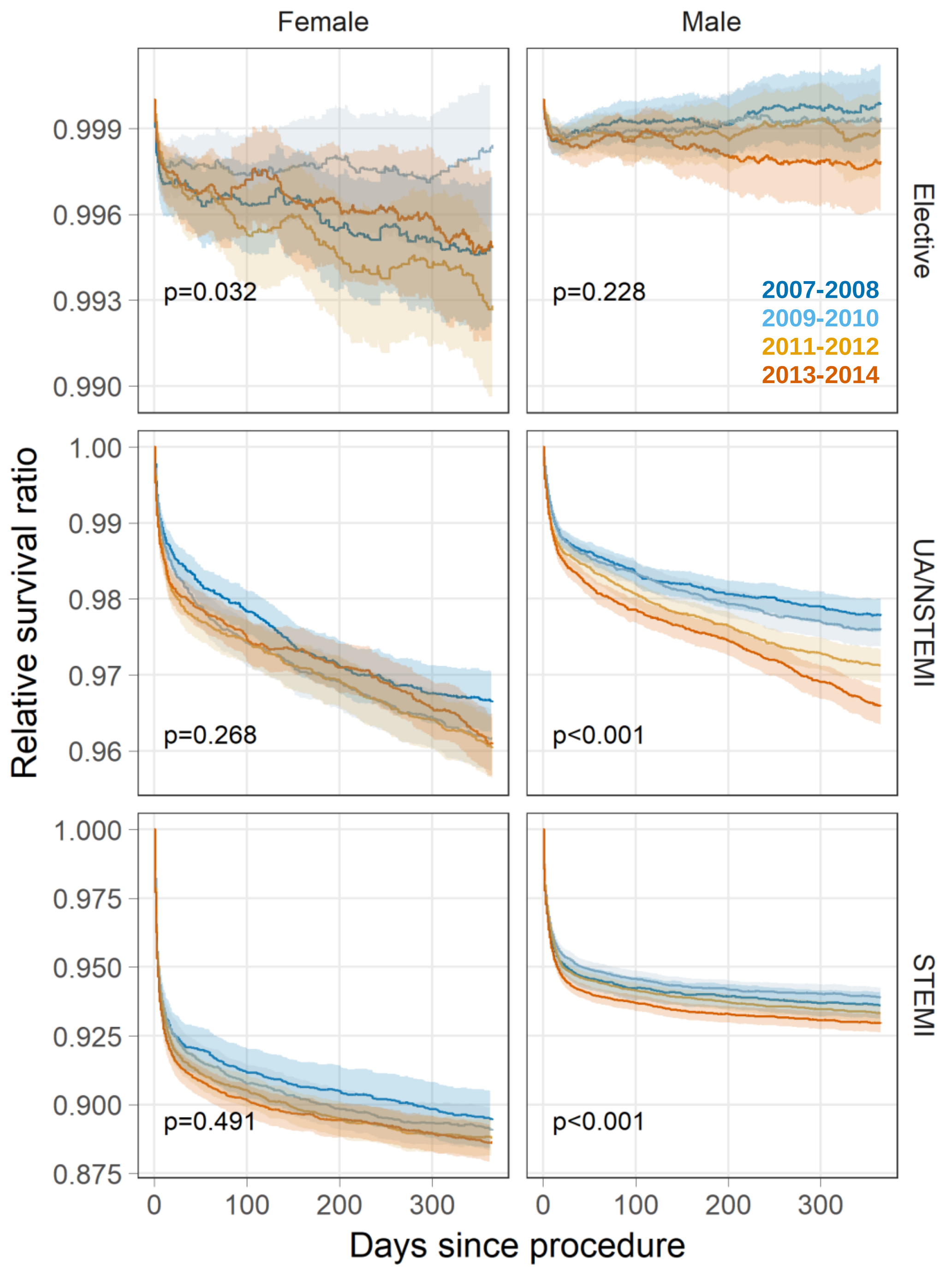

Figure A3 Observed-to-expected one-year mortality ratio (log-scale) by patient age, with 95% confidence limits.

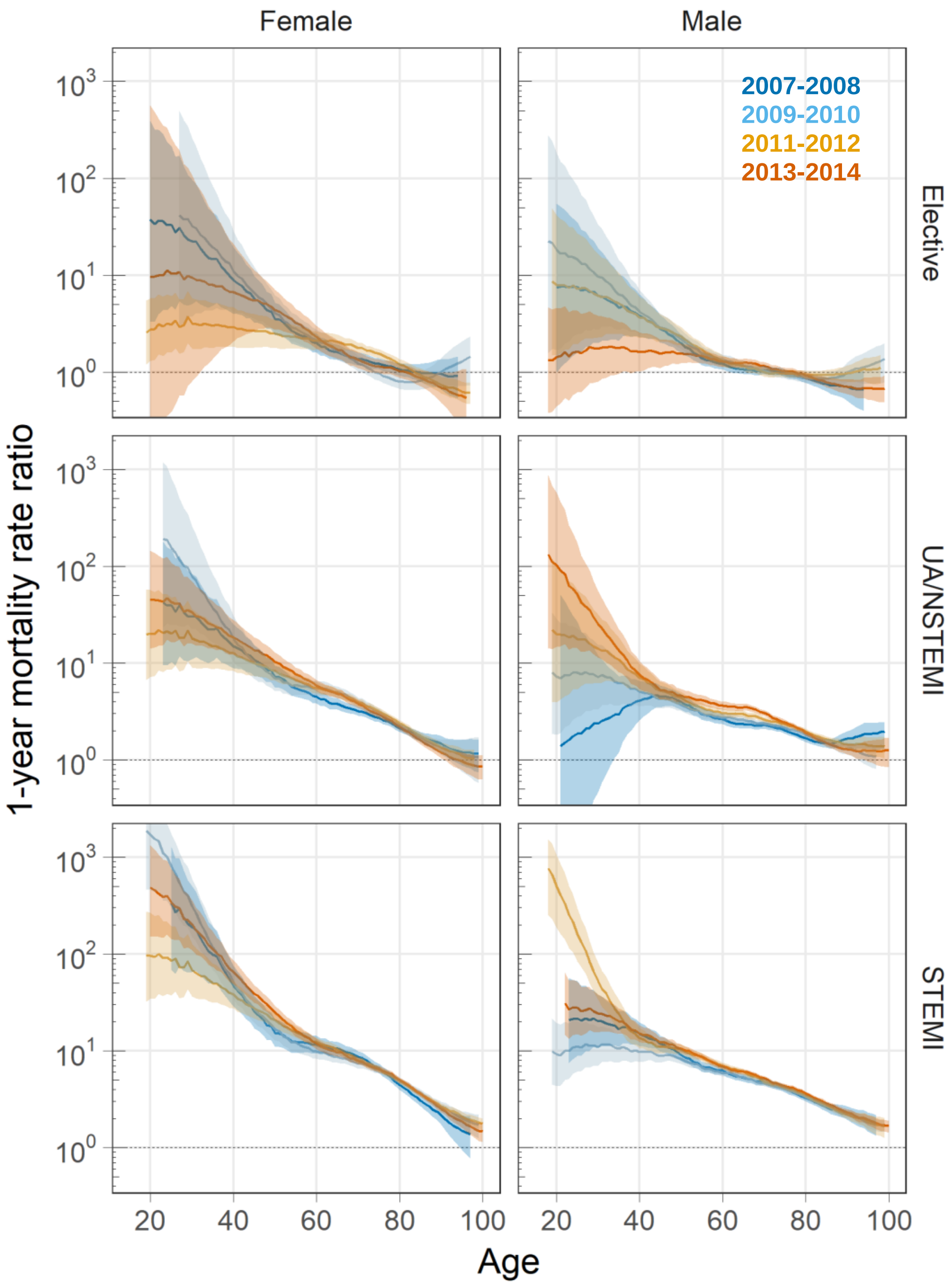

Binomial General Additive Models with 7-knot splines are used for smoothing the observed one-year mortality, then divided by the expected mortality rate. 95% confidence limits are removed for clarity.

Figure A4 Cumulative excess hazard rates by patient age, indication, and year of procedure, with 95% confidence limits.

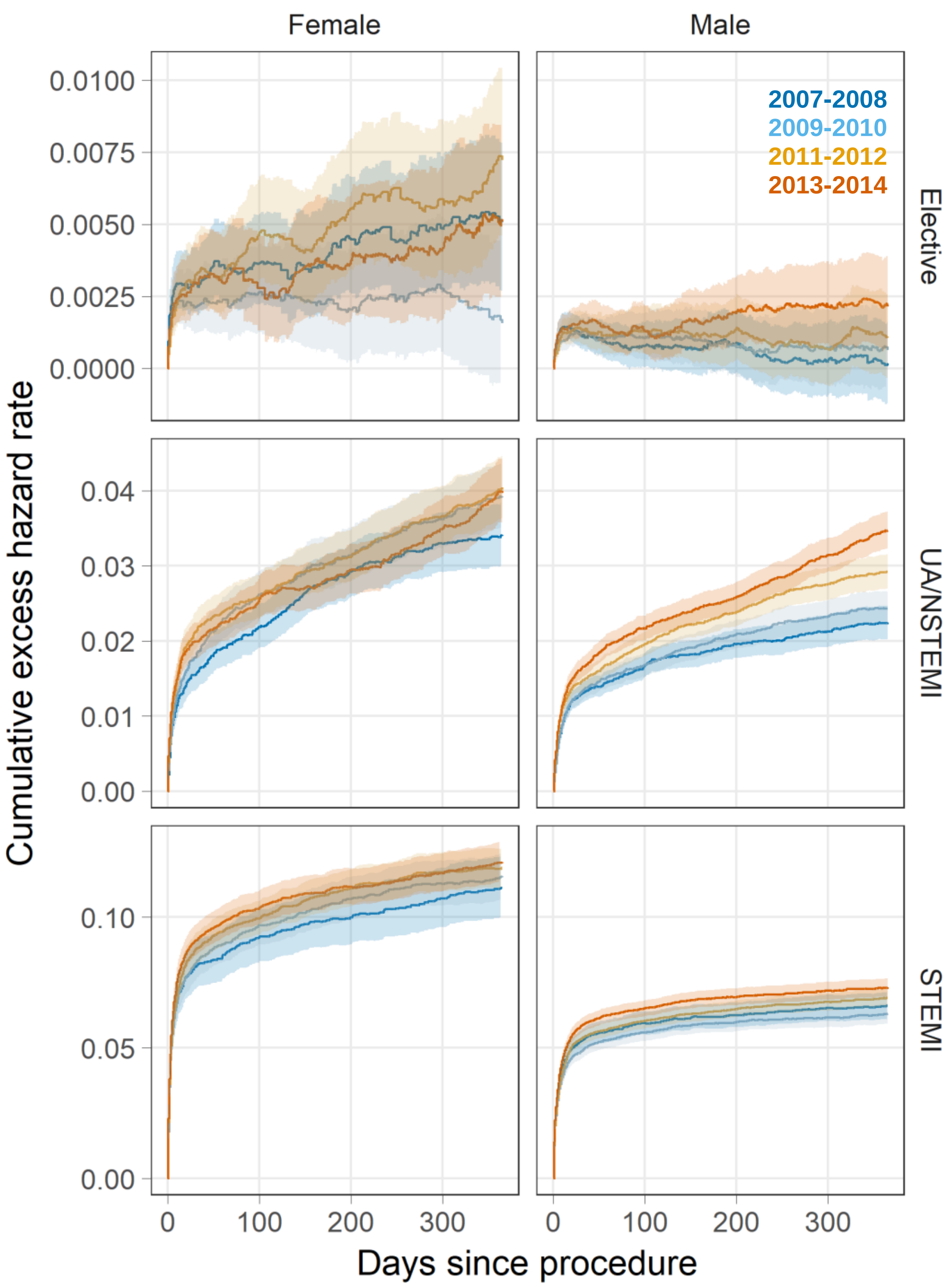

The RECORD statement – checklist of items, extended from the STROBE statement, that should be reported in observational studies using routinely collected health data.

	Item No.	STROBE items	Location in manuscript where items are reported	RECORD items	Location in manuscript where items are reported
**Title and abstract**					
	1	(a) Indicate the study's design with a commonly used term in the title or the abstract (b) Provide in the abstract an informative and balanced summary of what was done and what was found	Title page	RECORD 1.1: The type of data used should be specified in the title or abstract. When possible, the name of the databases used should be included.<br><br>RECORD 1.2: If applicable, the geographic region and timeframe within which the study took place should be reported in the title or abstract.<br><br>RECORD 1.3: If linkage between databases was conducted for the study, this should be clearly stated in the title or abstract.	1.1 Abstract: data<br><br>1.2 Abstract: setting<br><br>1.3 No individual patient record linkage within this study
**Introduction**					
Background rationale	2	Explain the scientific background and rationale for the investigation being reported	Page 3 Intro: para 1-3		
Objectives	3	State specific objectives, including any prespecified hypotheses	Page 3 Intro: para 4		
**Methods**					
Study Design	4	Present key elements of study design early in the paper	Page 4-5. Methods: statistical analysis		
Setting	5	Describe the setting, locations, and relevant dates, including periods of recruitment, exposure, follow-up, and data collection	Page 4-5. Methods		
Participants	6	*(a) Cohort study* - Give the eligibility criteria, and the	(a) Page 4. Methods: PCI procedure data	RECORD 6.1: The methods of study population selection (such as codes or	6.1 Page 4. Methods: PCI

		sources and methods of selection of participants. Describe methods of follow-up<br>*Case-control study* - Give the eligibility criteria, and the sources and methods of case ascertainment and control selection. Give the rationale for the choice of cases and controls<br>*Cross-sectional study* - Give the eligibility criteria, and the sources and methods of selection of participants<br><br>*(b) Cohort study* - For matched studies, give matching criteria and number of exposed and unexposed<br>*Case-control study* - For matched studies, give matching criteria and the number of controls per case	(b) Page 4. Methods: ONS life-table data	algorithms used to identify subjects) should be listed in detail. If this is not possible, an explanation should be provided.<br><br>RECORD 6.2: Any validation studies of the codes or algorithms used to select the population should be referenced. If validation was conducted for this study and not published elsewhere, detailed methods and results should be provided.<br><br>RECORD 6.3: If the study involved linkage of databases, consider use of a flow diagram or other graphical display to demonstrate the data linkage process, including the number of individuals with linked data at each stage.	procedure data<br><br>6.2 N/A No validation – exclusions only due to missing data<br><br>6.3 N/A No individual patient record linkage
Variables	7	Clearly define all outcomes, exposures, predictors, potential confounders, and effect modifiers. Give diagnostic criteria, if applicable.	Page 4. Methods. Mortality only outcome of interest	RECORD 7.1: A complete list of codes and algorithms used to classify exposures, outcomes, confounders, and effect modifiers should be provided. If these cannot be reported, an explanation should be provided.	7.1 N/A
Data sources/ measurement	8	For each variable of interest, give sources of data and details of methods of assessment (measurement).<br>Describe comparability of assessment methods if there is more than one group	Page 4. Methods. Only variables are age, sex, indication, procedure year.		
Bias	9	Describe any efforts to address potential sources of bias			
Study size	10	Explain how the study size was	N/A all available		

		arrived at	procedures included		
Quantitative variables	11	Explain how quantitative variables were handled in the analyses. If applicable, describe which groupings were chosen, and why	Page 4-5. Methods: statistical analysis		
Statistical methods	12	(a) Describe all statistical methods, including those used to control for confounding<br>(b) Describe any methods used to examine subgroups and interactions<br>(c) Explain how missing data were addressed<br>(d) *Cohort study* - If applicable, explain how loss to follow-up was addressed<br>*Case-control study* - If applicable, explain how matching of cases and controls was addressed<br>*Cross-sectional study* - If applicable, describe analytical methods taking account of sampling strategy<br>(e) Describe any sensitivity analyses	Page 4-5. Methods: statistical analysis		
Data access and cleaning methods		..	Page 5. Methods final para.	RECORD 12.1: Authors should describe the extent to which the investigators had access to the database population used to create the study population.<br><br>RECORD 12.2: Authors should provide information on the data cleaning methods used in the study.	Page 5. Methods: statistical analysis final para (+ GitHub page)
Linkage		..		RECORD 12.3: State whether the study included person-level, institutional-	N/A

For peer review only

				level, or other data linkage across two or more databases. The methods of linkage and methods of linkage quality evaluation should be provided.	
**Results**					
Participants	13	(a) Report the numbers of individuals at each stage of the study (*e.g.*, numbers potentially eligible, examined for eligibility, confirmed eligible, included in the study, completing follow-up, and analysed)<br>(b) Give reasons for non-participation at each stage.<br>(c) Consider use of a flow diagram	Page 7. Table 1<br><br>Supplementary Table A1	RECORD 13.1: Describe in detail the selection of the persons included in the study (*i.e.,* study population selection) including filtering based on data quality, data availability and linkage. The selection of included persons can be described in the text and/or by means of the study flow diagram.	Page 4. Methods: PCI procedure data<br><br>Supplementary Table A1
Descriptive data	14	(a) Give characteristics of study participants (*e.g.*, demographic, clinical, social) and information on exposures and potential confounders<br>(b) Indicate the number of participants with missing data for each variable of interest<br>(c) *Cohort study* - summarise follow-up time (*e.g.*, average and total amount)	(a, b) Page 8-10 Tables 2a, 2b, 2c<br><br>(c) Page 5. Results para 1		
Outcome data	15	*Cohort study* - Report numbers of outcome events or summary measures over time<br>*Case-control study* - Report numbers in each exposure category, or summary measures of exposure<br>*Cross-sectional study* - Report numbers of outcome events or summary measures	Figure 1<br><br>Supplementary Table A2		
Main results	16	(a) Give unadjusted estimates	(a) Figure 1		

For peer review only - http://bmjopen.bmj.com/site/about/guidelines.xhtml

		and, if applicable, confounder-adjusted estimates and their precision (e.g., 95% confidence interval). Make clear which confounders were adjusted for and why they were included<br>(b) Report category boundaries when continuous variables were categorized<br>(c) If relevant, consider translating estimates of relative risk into absolute risk for a meaningful time period	Tables 2a, 2b, 2c<br>Table A2<br><br>(b) N/A<br>(c) N/A		
Other analyses	17	Report other analyses done—e.g., analyses of subgroups and interactions, and sensitivity analyses	N/A		
**Discussion**					
Key results	18	Summarise key results with reference to study objectives	Page 11. Discussion para 2, 4		
Limitations	19	Discuss limitations of the study, taking into account sources of potential bias or imprecision. Discuss both direction and magnitude of any potential bias	Page 13. Discussion: limitations	RECORD 19.1: Discuss the implications of using data that were not created or collected to answer the specific research question(s). Include discussion of misclassification bias, unmeasured confounding, missing data, and changing eligibility over time, as they pertain to the study being reported.	
Interpretation	20	Give a cautious overall interpretation of results considering objectives, limitations, multiplicity of analyses, results from similar studies, and other relevant evidence	Page 11-12. Discussion para 2-8		
Generalisability	21	Discuss the generalisability (external validity) of the study results	Page 12. Discussion para 9		

For peer review only

**Other Information**					
Funding	22	Give the source of funding and the role of the funders for the present study and, if applicable, for the original study on which the present article is based	Provided in journal submission		
Accessibility of protocol, raw data, and programming code		..		RECORD 22.1: Authors should provide information on how to access any supplemental information such as the study protocol, raw data, or programming code.	GitHub page provided

*Reference: Benchimol EI, Smeeth L, Guttmann A, Harron K, Moher D, Petersen I, Sørensen HT, von Elm E, Langan SM, the RECORD Working Committee. The REporting of studies Conducted using Observational Routinely-collected health Data (RECORD) Statement. *PLoS Medicine* 2015; in press.

*Checklist is protected under Creative Commons Attribution (CC BY) license.

For peer review only
